# Regio- and stereoselective synthesis of new ensembles of diversely functionalized 1,3-thiaselenol-2-ylmethyl selenides by a double rearrangement reaction

**DOI:** 10.3762/bjoc.16.47

**Published:** 2020-03-27

**Authors:** Svetlana V Amosova, Andrey A Filippov, Nataliya A Makhaeva, Alexander I Albanov, Vladimir A Potapov

**Affiliations:** 1 A. E. Favorsky Irkutsk Institute of Chemistry, SD RAS, 1 Favorsky Str., 664033 Irkutsk, Russian Federation

**Keywords:** 2-(bromomethyl)-1,3-thiaselenole, nucleophilic addition, nucleophilic substitution, rearrangement, seleniranium intermediate

## Abstract

The reaction of 2-(bromomethyl)-1,3-thiaselenole with potassium selenocyanate proceeded via a rearrangement with ring expansion, leading to a six-membered 2,3-dihydro-1,4-thiaselenin-2-yl selenocyanate (kinetic product) which in turn underwent rearrangement with ring contraction to a 1,3-thiaselenol-2-ylmethyl selenocyanate (thermodynamic product). These rearrangements occurred by a nucleophilic attack of the selenocyanate anion at two different carbon atoms of the seleniranium intermediate. The efficient regioselective synthesis of alkyl, allyl, 2-propynyl, benzyl, 4-fluorobenzyl, and 2-pyridinylmethyl 1,3-thiaselenol-2-ylmethyl selenides was developed based on the generation of sodium 1,3-thiaselenol-2-ylmethylselenolate from 1,3-thiaselenol-2-ylmethyl selenocyanate or bis(1,3-thiaselenol-2-ylmethyl) diselenide followed by nucleophilic substitution reactions. Sodium 1,3-thiaselenol-2-ylmethylselenolate underwent nucleophilic addition to alkyl propiolates in a regio- and stereoselective manner affording 1,3-thiaselenol-2-ylmethyl vinyl selenides in high yields predominantly with *Z*-configuration. Not a single representative of the 1,3-thiaselenol-2-ylmethyl selenide scaffold has been previously described in the literature.

## Introduction

The regio- and stereoselective synthesis of organoselenium compounds based on selenium-centered electrophilic reagents has been one of the most important and effective directions in organoselenium chemistry for half a century [1–12].

The important trend in the field of organoselenium chemistry within the last 15 years was the involvement of selenium dihalides in the synthesis of organoselenium compounds [13–14]. The first synthesis of organoselenium compound from selenium dihalides was the preparation of 1,4-selenasilafulvenes by cyclization reaction with diethynyldimethylsilane [15–16]. The creation of new methodologies for the synthesis of new classes of organoselenium compounds and especially selenium heterocycles, with promising biological activity based on affordable and environmentally friendly materials is another important trend in organoselenium chemistry [17]. Reviews on the biological activity of organoselenium compounds reported examples exhibiting high antitumor, antiviral, antimicrobial, and neuroprotective activities [12,18–19]. A number of organoselenium compounds including selenoglutathion [20], *trans*-3,4-dihydroxyselenolane [21], and ascorbyl selenoesters [22] exhibit high glutathione peroxidase-like activity [12,18–19].

A series of novel approaches to functionalized organoselenium compounds was developed recently [23–29]. Among these is an elegant, highly effective approach that led to β-hydroxy, β-mercapto, and β-amino-substituted diorganyl diselenides and selenides. This was accomplished by the opening of three-membered oxygen-, sulfur- and nitrogen-containing heterocycles under the action of bis(trimethylsilyl)selenide [23]. The methodology based on bis(trimethylsilyl)selenide was also successfully applied to the synthesis of functionalized asymmetric alkyl- and vinyl selenides, including cyclic disubstituted 1,3-thiaselenolane and trisubstituted thiaselenane [26–27]. Vinyl selenides represent a very interesting class of compounds with a wide range of synthetic applications [28–29].

The anchimeric assistance effect, also known as the participation of neighbouring groups, is usually considered mainly as a factor accelerating the rate of nucleophilic substitution reactions. At present, we are observing a new manifestation of this effect, consisting in the promotion of new rearrangements leading to the selective formation of various selenium-containing unsaturated linear and heterocyclic compounds. This effect works in 2-(bromomethyl)-1,3-thiaselenole (**1**) – a unique reagent, which exhibits unusual properties in nucleophilic reactions. This reagent was obtained [30–32] in high yield and with high purity by a one-pot synthesis from divinyl sulfide [33–35] and selenium dibromide.

**Scheme 1:**
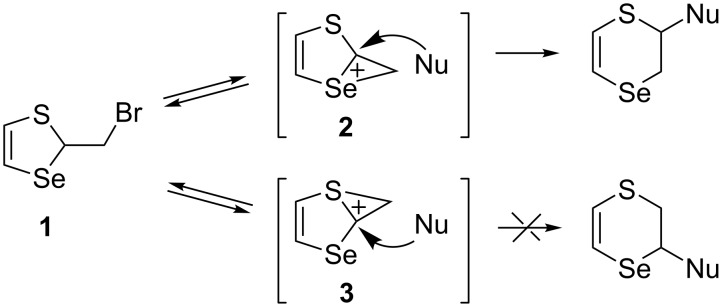
Possible formation of reaction products starting from **1** via seleniranium **2** or thiiranium cations **3**.

The structure of compound **1** suggests the possibility of formation of both seleniranium **2** and thiiranium **3** cations, whose participation in the nucleophilic substitution reaction will lead to two different reaction products (Scheme 1).

However, particularly, the reaction proceeds via an intermediate seleniranium cation **2**. This is explained by the high anchimeric assistance effect of the selenium atom which is more than one order of magnitude greater than the effect of the sulfur atom. This was established based on the determination of the absolute and relative rates of nucleophilic substitution of chlorine in 2,6-dichloro-9-selenabicyclo[3.3.1]nonane and 2,6-dichloro-9-thiabicyclo[3.3.1]nonane [36].

**Scheme 2:**
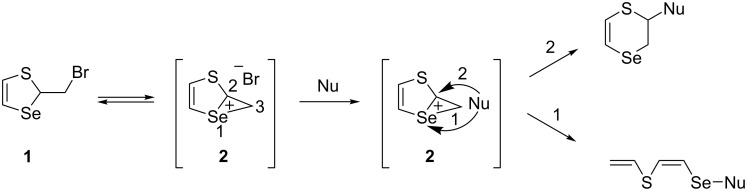
Unusual regio- and stereoselective nucleophilic reactions of thiaselenole **1** at two centers of the seleniranium cation **2**.

The nucleophilic substitution reactions in thiaselenole **1** with O- and S-centered nucleophiles proceeded regioselectively in an unusual manner at two centers of the seleniranium cation **2**: the selenium atom and the carbon atom C2. The classical nucleophilic substitution reaction at the carbon atom C3 did not occur (Scheme 2).

The high anchimeric assistance effect of the selenium atom in thiaselenole **1** is the driving force for the generation of the seleniranium intermediate **2**. A fundamental approach to the regio- and stereoselective synthesis of unsaturated functionalized organoselenium compounds was developed during the last ten years based on new directions for nucleophilic substitution reactions proceeding via intermediate seleniranium cations **2** generated from thiaselenole **1**.

**Scheme 3:**
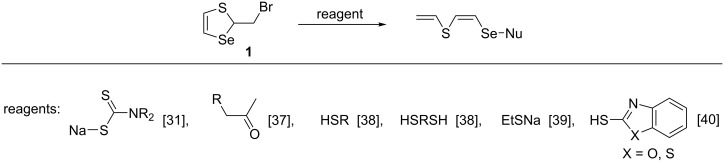
Reactions of thiaselenole **1** with С- and S-centered nucleophiles affording new families of linear unsaturated (*Z*)-2-[(organylsulfanyl)selanyl]ethenyl vinyl sulfides [31,37–40].

We have carried out new regioselective reactions of thiaselenole **1** with dithiocarbamates [31], ketones [37], thiols [38–39], dithiols [38], and mercapto benzazoles [40]. These are the first examples of a nucleophilic attack at the selenium atom of the seleniranium cation **2** with the generation of a new Se–S bond, yielding new families of linear unsaturated (*Z*)-2-[(organylsulfanyl)selanyl]ethenyl vinyl sulfides (Scheme 3).

**Scheme 4:**
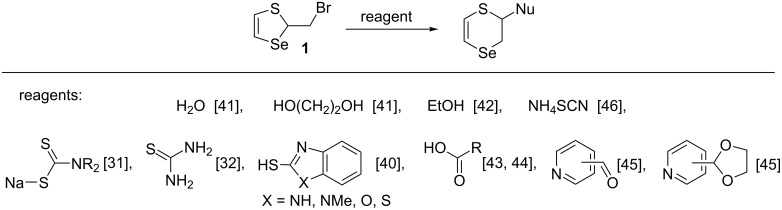
The reactions of thiaselenole **1** with the formation of polyfunctionalized 2,3-dihydro-1,4-thiaselenines.

Cascade regio- and stereoselective reactions of thiaselenole **1** with water and ethylene glycol resulted in the formation of the first representatives of the new family of polyfunctional 2,3-dihydro-1,4-thiaselenines [41]. The regioselective nucleophilic substitution reactions in thiaselenole **1** proceeding at the C2 carbon atom of the seleniranium cation **2** with dithiocarbomates [31], thiourea [32], alcohols [42], functionalized organic acids [43–44], functionalized pyridines [45], ammonium thiocyanate [46], and mercapto benzazoles [40] were developed (Scheme 4).

**Scheme 5:**
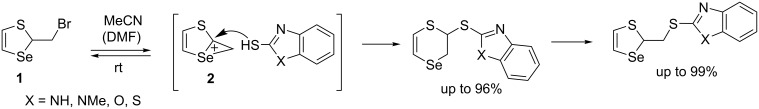
The synthesis of new 1,3-thiaselenole ensembles by reactions of thiaselenole **1** with mercapto benzazoles.

The new methodology of a regioselective nucleophilic substitution at three different centers of a seleniranium intermediate **2** in reactions of thiaselenole **1** with mercapto benzazoles containing various combinations of heteroatoms (N, O and S) was developed (Scheme 5). The reaction proceeded with the formation of a new family of 2,3-dihydro-1,4-thiaselenines (up to 96% yields) – products of rearrangement with ring expansion, which in turn underwent rearrangement with ring contraction, forming a new family of 1,3-thiaselenoles in up to 99% yield. The article was included in the RSC themed web collection "The chemistry of Selenium & Tellurium at the beginning of the 3rd millennium" [40].

Our short preliminary publication reported the formation of 1,3-thiaselenol-2-ylmethyl selenocyanate (**4**) in the reaction thiaselenole **1** with selenocyanate [47].

## Results and Discussion

In this work the nucleophilic substitution reaction of thiaselenole **1** with potassium selenocyanate was studied in detail. We found that the reaction of thiaselenole **1** with KSeCN at room temperature afforded a five-membered heterocycle, selenocyanate **4**, in a quantitative yield. This result was unusual since previously studied substitution reactions of thiaselenole **1** with various nucleophiles (Scheme 4) were accompanied by ring expansion leading to six-membered functionalized dihydrothiaselenines. Furthermore, the reaction of thiaselenole **1** with ammonium thiocyanate also gave the six-membered 2,3-dihydro-1,4-thiaselenin-2-yl thiocyanate (Scheme 4) [46].

**Scheme 6:**
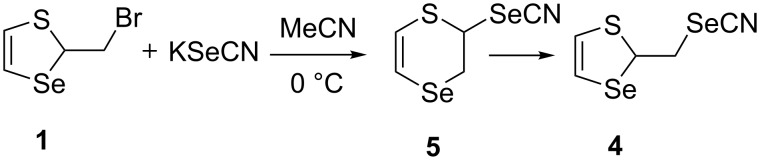
The formation of product **4** via compound **5** by the reaction of thiaselenole **1** with potassium selenocyanate.

It was suggested that the reaction proceeds via an intermediate six-membered selenocyanate **5**, which could be observed at a lower temperature. Indeed, monitoring the reaction by ^1^H NMR spectroscopy using the same conditions as in the synthesis of the selenocyanate **4**, but at 0 °C confirmed this assumption (Scheme 6).

**Figure 1:**
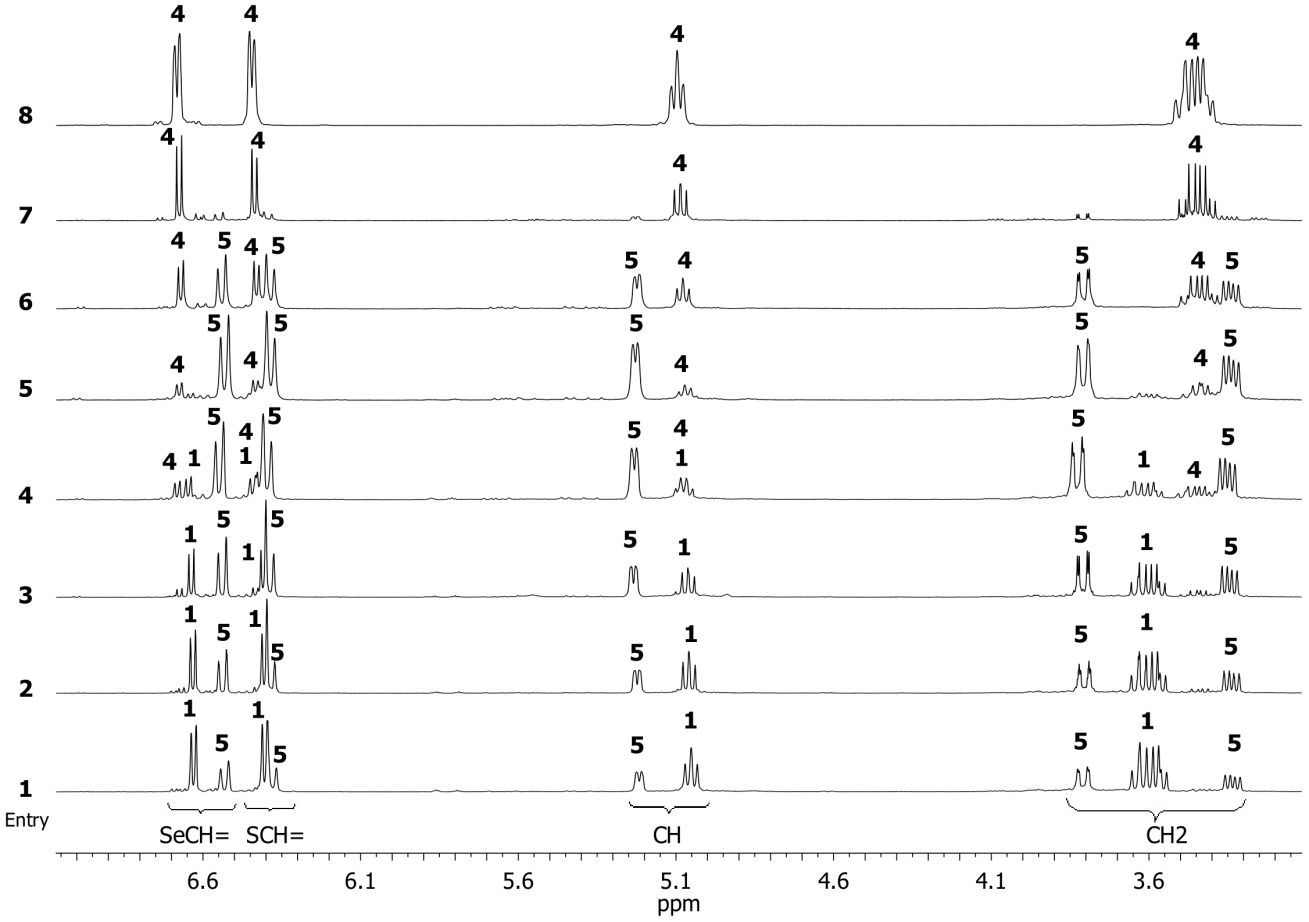
Monitoring the reaction of thiaselenole **1** with KSeCN by ^1^H NMR spectroscopy (in accordance with the data of Table 1). Reaction conditions: compound **1** (0.5 mmol), KSeCN (0.5 mmol), MeCN (2.5 mL), 0 °C.

The monitoring results are presented in Figure 1 and Table 1. The reaction was studied in the time interval from 0.25 to 6 h. Having assigned the ^1^H NMR data for compounds **1** and **4**, we found signals of an unknown compound, which was easily identified after 0.5 h (at 46% conversion of thiaselenole **1**) as the six-membered selenocyanate **5** (Table 1, entry 2). The vinyl proton signals of the SeCH= groups (6.63 ppm, ^3^*J*_Н,H_ = 6.3 Hz, compound **1**; 6.67 ppm, ^3^*J*_Н,H_ = 6.3 Hz, compound **4**; 6.53 ppm, ^3^*J*_Н,H_ = 9.9 Hz, compound **5**) were used to estimate the compounds ratios. The signals of the two protons of the SeCH_2_ group in the six-membered ring **5** are not equivalent. As consequence they are observed in the ^1^H NMR spectrum as two doublets of doublets with geminal and vicinal coupling constants with the SCH-proton in the same ring (3.79 ppm, ^2^*J*_Н,Н_ = 12.4 Hz, ^3^*J*_Н,Н_ = 2.1 Hz; 3.33 ppm, ^2^*J*_Н,Н_ = 12.4 Hz, ^3^*J*_Н,Н_ = 6.6 Hz). The maximum content of compound **5** (80%) was reached after 4 h at a molar ratio of **1**:**5**:**4** = 6:80:14, Table 1, entry 5). Afterwards, the content of compound **5** dropped sharply with an increase of the content of compound **4** from 14 to 41% and the complete conversion of thiaselenole **1** at molar ratio of **1**:**5**:**4** = 0:59:41, Table 1, entry 6). The comparison of entries 5 and 6 (Table 1) clearly indicates that compound **4** was formed due to a rearrangement of selenocyanate **5** rather than by the “classical” nucleophilic substitution of bromine in thiaselenole **1**. The rearrangement of selenocyanate **5** to compound **4** proceeded already at 0 °C, and therefore we were not able to isolate compound **5** under these conditions.

**Table 1:** Results the reaction of thiaselenole **1** with KSeCN based on ^1^H NMR spectroscopy monitoring (Figure 1).^a^

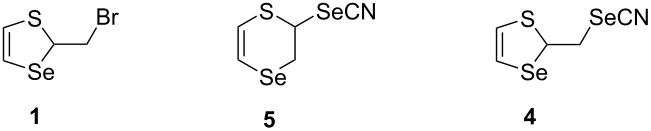					

entry	time, h	molar ratio (%)			conversion of **1** (%)
		**1**	**5**	**4**	

1	0.25	63	37	0	37
2	0.5	54	46	0	46
3	1	33	61	6	67
4	2	16	71	13	84
5	4	6	80	14	94
6	6	0	59	41	100
7^b^	4 + 1	0	14	86	100
8^с^	6 + 1	0	0	100	100

^a^Reaction conditions: compound **1** (0.5 mmol), KSeCN (0.5 mmol), MeCN (2.5 mL), 0 °C. ^b^4 h at 0 °C and 1 h without cooling. ^с^6 h at 0 °C and 1 h without cooling.

The formation of the six-membered heterocycle of 2,3-dihydro-1,4-thiaselenin-2-yl selenocyanate **5** was confirmed, inter alia, by the observation of two doublets corresponding to the olefinic protons of the SCH=CHSe group with ^3^*J* = 9.9 Hz in the ^1^H NMR spectrum (Table 1, entry 2). In the case of five-membered compounds **1** and **4**, the coupling constant of the olefinic protons of the SCH=CHSe group would be ^3^*J*_H,H_ = 6.3 Hz [32,40]. The SCHSe group of the heterocycle **5** was characterized by a doublet of doublets at 5.21 ppm (^3^*J* = 2.1 and ^3^*J* = 6.6 Hz). This was the result of coupling with the nonequivalent protons of the CH_2_Se group. Similar spectral characteristics were observed for other six-membered 2,3-dihydro-1,4-thiaselenin-2-yl derivatives [31–32,38,40].

**Scheme 7:**
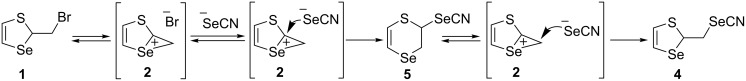
The reaction pathway for the formation of compounds **4** and **5**.

Thus, the reaction of thiaselenole **1** with potassium selenocyanate led to the six-membered thiaselenine **5** (kinetic product), which underwent a rearrangement to a five-membered thiaselenole selenocyanate **4** (thermodynamic product). These rearrangements proceeded by a nucleophilic attack of the selenocyanate anion on two different carbon atoms of the seleniranium intermediate **2** (Scheme 7).

The formation of thiaselenole **4** was the result of two rearrangements, rather than a “classical” nucleophilic substitution of the bromine atom in thiaselenole **1**.

Similar rearrangements were found and studied in the reactions of thiaselenole **1** with mercaptobenzazoles [40].

Having established the synthesis of heterocycle **4** this unsaturated five-membered S,Se-containing compound was used as a starting material for the synthesis of novel ensembles of selenium heterocycles with promising biological activity.

**Scheme 8:**
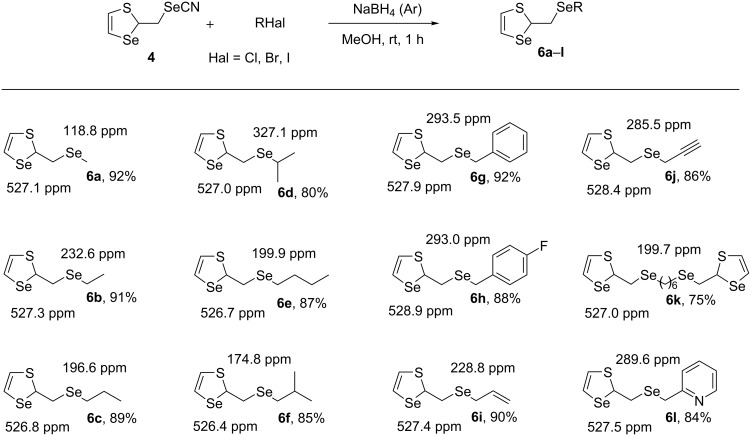
Synthesis of new ensembles of 1,3-thiaselenol-2-ylmethyl selenides **6a**–**l** (^77^Se NMR data are included).

The treatment of **4** with organyl halides resulted in the efficient, regioselective synthesis of hitherto unknown organyl 1,3-thiaselenol-2-ylmethyl selenides **6a–l** in high yields (Scheme 8). The synthesis was based on the generation of sodium 1,3-thiaselenol-2-ylmethylselenolate by the reaction of NaBH_4_ with compound **4** in methanol followed by nucleophilic substitution reactions with organyl halides. Various substrates were involved in the reaction, providing the introduction of alkyl, benzyl, allyl, and 2-propynyl moieties as well as pharmacophores as 2-pyridylmethyl and 4-fluorobenzyl. A possibility for the synthesis of compounds containing two 1,3-thiaselenol-2-ylmethylselanyl heterocycles connected by a carbon bridge was demonstrated (e.g., compound **6k** containing two 1,3-thiaselenol-2-ylmethylselanyl heterocycles connected by a six-carbon bridge).

**Scheme 9:**
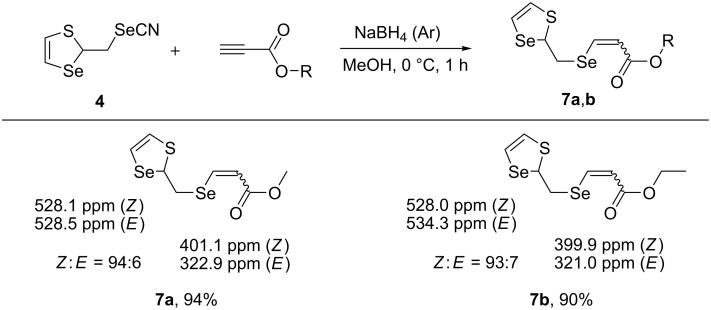
The synthesis of vinyl selenides **7a**,**b** through nucleophilic addition of 1,3-thiaselenol-2-ylmethylselenolate anion to alkyl propiolates (^77^Se NMR data are included).

The 1,3-thiaselenol-2-ylmethylselenolate anion generated from selenocyanate **4** was also subjected to a nucleophilic addition reaction with activated acetylenes (Scheme 9). The reaction of the 1,3-thiaselenol-2-ylmethylselenolate anion with alkyl propiolates proceeded in a regio- and stereoselective manner affording alkyl 3-[(1,3-thiaselenol-2-ylmethyl)selanyl]-2-propenoates **7a** (*Z*/*E* = 94:6) and **7b** (*Z*/*E* = 93:7) in 94% and 90% yields, respectively.

**Scheme 10:**
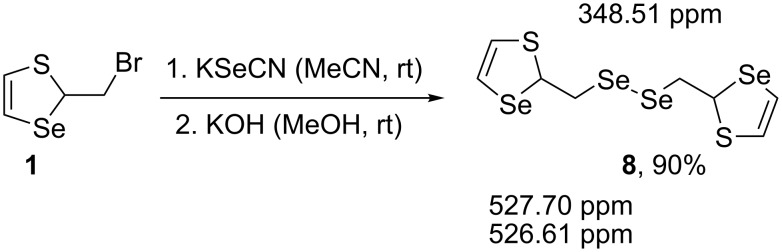
One-pot synthesis of diselenide **8** from thiaselenole **1** (^77^Se NMR data are included).

The one-pot synthesis of hitherto unknown bis(1,3-thiaselenol-2-ylmethyl) diselenide (**8**) in 90% yield from thiaselenole **1** was developed (Scheme 10). The reaction proceeded via the formation of thiaselenole selenocyanate **4**, which was in situ converted into diselenide **8**. This compound consisted of two diastereomers, and two signals corresponding to each of them were observed in the ^77^Se NMR spectrum.

**Scheme 11:**
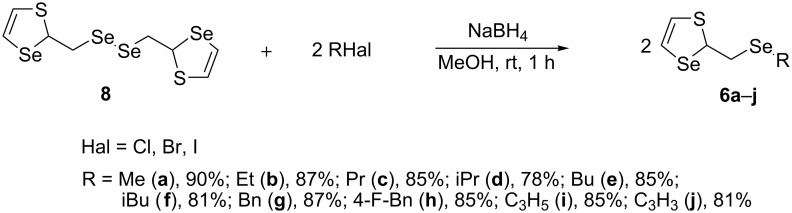
Synthesis of compounds **6a**–**j** from diselenide **8**.

Diselenide **8** was used as a starting material for the synthesis of 1,3-thiaselenol-2-ylmethylselanyl derivatives. Compounds **6a**–**j** were prepared in 78–90% yields via the generation of sodium 1,3-thiaselenol-2-ylmethylselenolate through reduction of the Se–Se bond with NaBH_4_ followed by nucleophilic substitution with alkyl halides (Scheme 11).

Thus, two efficient methods for the preparation of novel 1,3-thiaselenol-2-ylmethylselanyl derivatives **6a**–**l** from selenocyanate **4** and diselenides **8** were developed. Selenocyanate **4** and diselenide **8** were shown to be the synthons of the 1,3-thiaselenol-2-ylmethylselenolate anion, which was involved in the nucleophilic substitution reactions and regio- and stereoselective nucleophilic additions to activated acetylenes. However, the method to generate the 1,3-thiaselenol-2-ylmethylselenolate anion from selenocyanate **4** seemed to be more efficient, and the yields of the thiaselenol-2-ylmethylselanyl derivatives **6a**–**l** from selenocyanate **4** were slightly higher compared to that of the method based on diselenide **8**.

The structures of compounds **6a**–**l**, **7a**,**b** and **8** were proved by ^1^H, ^13^C, ^15^N, ^19^F and ^77^Se NMR and mass spectrometry. The composition of the products was confirmed by elemental analysis. Molecular ions of the synthesized compounds were observed in their mass spectra.

Two doublets of the olefinic protons of the SCH=CHSe group with ^3^*J* = 6.3–6.6 Hz were observed in the ^1^H NMR spectra of heterocycles **4**, **6a**–**l**, **7a**,**b** and **8**. These values are typical for five-membered 1,3-thiaselenoles [31–32]. The structure of 1,3-thiaselenol-2-ylmethyl derivatives **4**, **6a**–**l**, **7a**,**b** and **8** was confirmed, inter alia, by the spin–spin coupling constants between the selenium atom and the carbon atom of the SCHSe group in the ring. The observed values (66.1–68.2 Hz) were characteristic for direct coupling constants (^1^*J*_Se-C_).

The signals of the selenium atoms in the ^77^Se NMR spectra of the synthesized compounds **6a**–**l**, **7a**,**b**, and **8** were seen in the region of 526.41–534.48 ppm (for the ring) and 148.82–401.09 ppm (in the side chain).

## Conclusion

The reaction of 2-(bromomethyl)-1,3-thiaselenole (**1**) with potassium selenocyanate was found to occur with a rearrangement and ring expansion leading to six-membered 2,3-dihydro-1,4-thiaselenin-2-yl selenocyanate **5**, which at room temperature underwent rearrangement and ring contraction to 1,3-thiaselenol-2-ylmethyl selenocyanate **4**. These rearrangements proceeded via a seleniranium cation **2** that is attacked by the nucleophilic selenocyanate anion at two different carbon atoms of the three-membered ring.

The regio- and stereoselective synthesis of the novel ensembles of diverse 1,3-thiaselenol-2-ylmethyl selenides **6a**–**l, 7a**,**b** and diselenide **8** in high yields was developed based on the generation of a 1,3-thiaselenol-2-ylmethylselenolate anion, which was involved in three different reactions: nucleophilic substitution with a wide range of organic halides, regio- and stereoselective nucleophilic addition to activated acetylenes and oxidation. Noteworthy, no other example for the synthesis of 1,3-thiaselenol-2-ylmethyl selenide derivatives was hitherto described.

A high selectivity, mild reaction conditions and very simple work-up procedures are important features of this approach. The obtained compounds represent novel ensembles of functionalized unsaturated five-membered heterocycles containing one sulfur atom and two selenium atoms of different nature (Schemes 8–11), which are valuable scaffolds for organic synthesis and medicinal chemistry.

## Supporting Information

File 1 Experimental section and ^1^H, ^13^C, ^15^N, ^19^F and ^77^Se NMR spectra of all synthesized compounds.
