# Supplementary material for: Regio- and stereoselective synthesis of new ensembles of diversely functionalized 1,3-thiaselenol-2-ylmethyl selenides by a double rearrangement reaction
Source: Beilstein J Org Chem. 2020 Mar 27;16:515–23. doi: 10.3762/bjoc.16.47 (PMC7113545; doi:10.3762/bjoc.16.47)
Supplement: File 1 — Experimental section and 1H, 13C, 15N, 19F and 77Se NMR spectra of all synthesized compounds. [file Beilstein_J_Org_Chem-16-515-s001.pdf]

BEILSTEIN JOURNAL OF ORGANIC CHEMISTRY

Supporting Information

for

Regio- and stereoselective synthesis of new ensembles of diversely functionalized 1,3-thiaselenol-2-ylmethyl selenides by a double rearrangement reaction

Svetlana V. Amosova, Andrey A. Filippov, Nataliya A. Makhaeva, Alexander I. Albanov and Vladimir A. Potapov

Beilstein J. Org. Chem. **2020,** *16,* 515–523. doi:10.3762/bjoc.16.47

Experimental section and ^{1}H, ^{13}C, ^{15}N, ^{19}F and ^{77}Se NMR spectra of all synthesized compounds

License and Terms: This is a supporting information file under the terms of the Creative Commons Attribution License (http://creativecommons.org/licenses/by/4.0). Please note that the reuse, redistribution and reproduction in particular requires that the authors and source are credited.
The license is subject to the *Beilstein Journal of Organic Chemistry* terms and conditions: (https://www.beilstein-journals.org/bjoc)

Experimental

General. ^{1}H NMR (400.1 MHz), ^{13}C NMR (100.6 MHz), ^{15}N NMR (40.56 MHz), ^{19}F NMR (376 MHz) and ^{77}Se NMR (76.3 MHz) spectra were recorded on a Bruker DPX-400 spectrometer as 5–10% solutions in $CDCl_3$ referenced to residual chloroform (7.27 ppm, ^{1}H; 77.00 ppm, ^{13}C), $MeNO_2$ (^{15}N NMR, external), $CFCl_3$ (^{19}F NMR, external) and Me_2Se (^{77}Se NMR, external). Mass spectra were recorded on an Agilent 5975 with electron impact (EI) ionization at 70 eV. The C, H, N and S elemental analyses were performed on a Thermo flash 2000 analyzer. Analytical determination of bromine and selenium was made by known volumetric methods. Alfa Aesar silica gel (70–230 mesh) was used for column chromatography.

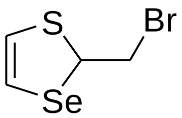

2-(Bromomethyl)-1,3-thiaselenole (1): Thiaselenole **1** was prepared from $SeBr_2$ and divinyl sulfide according to the previously described procedure [1].

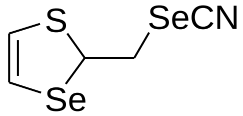

1,3-Thiaselenol-2-ylmethyl selenocyanate (4): Selenocyanate **4** was prepared in a similar manner as described before [2] in 99% yield. ^{1}H NMR (400 MHz, $CDCl_3$), δ (ppm): 6.67 (d, $^{3}J_{H,H}$ = 6.3 Hz, $^{2}J_{Se,H}$ = 48.5 Hz, 1H, SeCH=), 6.43 (d, $^{3}J_{H,H}$ = 6.3 Hz, 2H, SCH=), 5.08 (dd, $^{3}J_{H,H}$ = 7.1 Hz, $^{3}J_{H,H}$ = 8.2Hz, $^{2}J_{Se,H}$ = 20.0 Hz, 1H, SCHSe), 3.48 (dd, $^{3}J_{H,H}$ = 7.1 Hz, $^{2}J_{H,H}$ = 12.2 Hz, 1H, CH_2SeCN), 3.41 (dd, $^{2}J_{H,H}$ = 12.2 Hz, $^{3}J_{H,H}$ = 8.2 Hz, 1H, CH_2SeCN). $^{13}C\{^{1}H\}$ NMR (100 MHz, $CDCl_3$), δ (ppm): 119.26 (SCH=), 113.75 ($^{1}J_{Se,C}$ = 106 Hz, SeCH=), 101.07 (SeCN), 46.48 ($^{1}J_{Se,C}$ = 70 Hz, SCHSe), 37.97 ($^{1}J_{Se,C}$ = 52 Hz, $\underline{C}H_2SeCN$). $^{77}Se\{^{1}H\}$ NMR (76.3 MHz, $CDCl_3$), δ (ppm): 539.61(SCHSe), 228.41 (CH_2SeCN). Anal. Calcd for $C_5H_5NSSe_2$: C 22.32; H 1.87; N 5.21; S 11.92: Se, 58.69. Found: C 21.92; H 1.83; N 4.87; S 12.30: Se, 59.10.

---

1. Amosova, S. V.; Novokshonova, I. A.; Penzik, M. V.; Filippov, A. S.; Albanov, A. I.; Potapov, V. A. *Tetrahedron Lett.* **2017**, *58*, 4381-4383. doi:10.1016/j.tetlet.2017.10.011
2. Potapov, V. A.; Filippov, A. S.; Amosova, S. V. *Russ. J. Org. Chem.* **2018**, *54*, 957-958; *Zhurn. Org. Khim.* **2018**, *54*, 949-950. doi:10.1134/S1070428018060246

Monitoring the reaction of thiaselenole 1 with KSeCN by ^{1}H NMR spectroscopy: Potassium selenocyanate (576 mg, 4 mmol) was completely dissolved in MeCN (20 mL) and this solution was divided into 8 equal parts. Eight identical samples were prepared by adding a cooled (0 °C) solution of KSeCN (72 mg, 0.5 mmol) in MeCN (2.5 mL) to thiaselenole **1** (122 mg, 0.50 mmol) with stirring. Each samples was stirred in the ice bath at 0 °C from 0.25 h to 6 h (entries 1–6, Table 1). Entry 7 (Table 1): the sample was stirred for 4 h at 0 °C and 1 h without cooling. Entry 8 (Table 1): the sample was stirred for 6 h at 0 °C and for 1 h without cooling. Then the solvent was removed in vacuum giving a light yellow oil, which was analyzed by ^{1}H NMR spectroscopy (Figure 1).

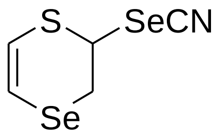

2,3-Dihydro-1,4-thiaselenin-2-yl selenocyanate (5): A cooled (0 °C) solution of potassium selenocyanate (72 mg, 0.50 mmol) in MeCN (0.25 mL) was added to a cooled (0 °C) solution of thiaselenole **1** (122 mg, 0.50 mmol) in MeCN (0.25 mL) with stirring. The mixture was stirred in an ice bath at ≈ 0 °C for 0.5 h and the solvent was removed in vacuum giving a light yellow oil, which was analyzed by ^{1}H and ^{13}C NMR spectroscopy. The mixture contained thiaselenole **1** and selenocyanate **5** (54:46 molar ratio according to ^{1}H NMR data). The following spectral characteristics of selenocyanate **5** were detected. ^{1}H NMR (400 MHz, $CDCl_3$), δ (ppm): 6.53 (dd, $^3J_{H,H}$ = 9.9 Hz, 1H, SeCH=), 6.37 (d, $^3J_{H,H}$ = 9.9 Hz, 1H, SCH=), 5.21 (dd, $^3J_{H,H}$ = 2.1 Hz, $^3J_{H,H}$ = 6.6 Hz, 1H, SCHSe), 3.79 (dd, $^2J_{H,H}$ = 12.4 Hz, $^3J_{H,H}$ = 2.1 Hz, 1H, =CHSeC$\underline{H}_2$), 3.33 (dd, $^2J_{H,H}$ = 12.4 Hz, $^3J_{H,H}$ = 6.6 Hz, 1H, =CHSeC$\underline{H}_2$). ^{13}C{^{1}H} NMR (100 MHz, $CDCl_3$), δ (ppm): 116.76 (SCH=), 110.66 (SeCH=), 101.88 (SeCN), 43.07 (SCHSe), 26.07 ($SeCH_2$).

General procedure for the synthesis of organyl 1,3-thiaselenol-2-ylmethyl selenides (6a–l) from selenocyanate 4: A solution of the organic halide (1.1 mmol) in MeOH (1 mL) was added to the solution of selenocyanate **4** (269 mg, 1 mmol) in MeOH (1 mL) under argon. Then $NaBH_4$

(87 mg, 2.3 mmol) was added portionwise to the stirred mixture, followed by stirring for 1 h at room temperature under argon. The mixture was diluted with cold degassed water (15 mL) and extracted with $CHCl_3$ (3 × 10 mL). The organic phase was dried over $CaCl_2$ and the solvent was removed by rotary evaporation. The products **6a–l** were purified by column chromatography (silica gel, eluent: hexane $\rightarrow$ hexane/$CHCl_3$ 4:1).

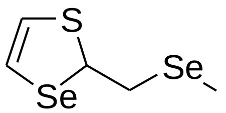

Methyl 1,3-thiaselenol-2-ylmethyl selenide (6a): Compound **6a** was obtained as light yellow oil using iodomethane (156 mg, 1.1 mmol). Yield: 237 mg (92%) (from selenocyanate **4**) and 232 mg (90%) (from diselenide **8**). ^{1}H NMR (400 MHz, $CDCl_3$), δ (ppm): 6.61 (d, $^{3}J_{H,H}$ = 6.6 Hz, $^{2}J_{Se,H}$ = 48.2 Hz, 1H, SeCH=), 6.41 (d, $^{3}J_{H,H}$ = 6.6 Hz, 1H, SCH=), 5.06 (t, $^{3}J_{H,H}$ = 7.8 Hz, 1H, SCHSe), 3.05 (dd, $^{2}J_{HH}$ = 12.9 Hz, $^{3}J_{H,H}$ = 7.8 Hz, 1H, CH_2Se), 2.94 (dd, $^{2}J_{HH}$ = 12.9 Hz, $^{3}J_{H,H}$ = 7.8 Hz, 1H, CH_2Se), 2.09 (s, $^{2}J_{Se,H}$ = 10.6 Hz, 3H, CH_3). $^{13}C\{^{1}H\}$ NMR (100 MHz, $CDCl_3$), δ (ppm): 119.54 (SCH=), 113.20 ($^{1}J_{Se,C}$ = 106.8 Hz, SeCH=), 48.11 ($^{1}J_{Se,C}$ = 66.1 Hz, SCHSe), 35.36 ($^{1}J_{Se,C}$ = 66.2 Hz, $^{2}J_{Se,C}$ = 7.0 Hz, CH_2Se), 5.30 ($^{1}J_{Se,C}$ = 62.7 Hz, CH_3). $^{77}Se\{^{1}H\}$ NMR (76.3 MHz, $CDCl_3$), δ (ppm): 527.11 (SCHSe), 118.82 ($SeCH_3$). MS (EI): *m/z* (%) = 260 (23, M^+), 165 (11), 151 (100), 107 (7), 85 (36), 84 (34), 59 (22), 58 (32). Anal. Calcd for $C_5H_8SSe_2$: C, 23.27; H, 3.12; S, 12.42; Se, 61.18. Found: C, 23.28; H, 3.17; S, 12.11; Se, 61.36.

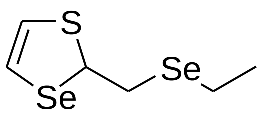

Ethyl 1,3-thiaselenol-2-ylmethyl selenide (6b): Compound **6b** was obtained as light yellow oil using 1-iodoethane (172 mg, 1.1 mmol). Yield: 248 mg (91%) (from selenocyanate **4**) and 237 mg (87%) (from diselenide **8**). ^{1}H NMR (400 MHz, $CDCl_3$), δ (ppm): 6.61 (d, $^{3}J_{H,H}$ = 6.3 Hz, $^{2}J_{Se,H}$ = 48.1 Hz, 1H, SeCH=), 6.41 (d, $^{3}J_{H,H}$ = 6.3 Hz, 1H, SCH=), 5.04 (t, $^{3}J_{H,H}$ = 7.8 Hz, 1H, SCHSe), 3.08 (dd, $^{2}J_{H,H}$ = 12.8 Hz, $^{3}J_{H,H}$ = 7.8 Hz, 1H, $CHC\underline{H}_2Se$), 2.97 (dd, $^{2}J_{H,H}$ = 12.8 Hz, $^{3}J_{H,H}$ = 7.8 Hz, 1H, $CHC\underline{H}_2Se$), 2.68 (q, $^{2}J_{H,H}$ = 7.5 Hz, 2H, $C\underline{H}_2CH_3$), 1.41 (t, $^{2}J_{H,H}$ 7.5 Hz, 3H, CH_3). $^{13}C\{^{1}H\}$ NMR (100 MHz, $CDCl_3$), δ (ppm): 119.51 (SCH=), 113.18 ($^{1}J_{Se,C}$ = 106.7 Hz, SeCH=), 48.56 ($^{1}J_{Se,C}$ =

67.6 Hz, SCHSe), 33.30 ($^{1}J_{Se,C}$ = 67.9 Hz, $^{2}J_{Se,C}$ = 6.7 Hz, $CH\underline{C}H_2Se$), 18.55 ($^{1}J_{Se,C}$ = 59.2 Hz, $\underline{C}H_2CH_3$), 15.90 ($^{2}J_{Se,C}$ = 9.4 Hz, CH_3). ^{77}Se{^{1}H} NMR (76.3 MHz, $CDCl_3$), δ (ppm): 527.25 (SCHSe), 232.55 ($SeCH_2CH_3$). MS (EI): *m/z* (%) = 272 (20, M^+), 165 (15), 151 (100), 107 (9), 85 (42), 84 (34), 59 (25), 58 (35), 45 (25). Anal. Calcd for $C_6H_{10}SSe_2$: C, 26.48; H, 3.70; S, 11.78; Se, 58.03. Found: %: C 26.78; H 3.62; S 11.58; Se, 57.88.

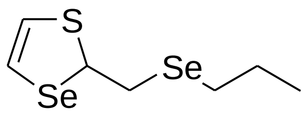

Propyl 1,3-thiaselenol-2-ylmethyl selenide (6c): Compound **6c** was obtained as light yellow oil using 1-bromopropane (135 mg, 1.1 mmol). Yield: 255 mg (89%) (from selenocyanate **4**) and 243 mg (85%) (from diselenide **8**). ^{1}H NMR (400 MHz, $CDCl_3$), δ (ppm): 6.61 (d, $^{3}J_{H,H}$ = 6.3 Hz, $^{2}J_{Se,H}$ = 47.9 Hz, 1H, SeCH=), 6.41 (d, $^{3}J_{H,H}$ = 6.3 Hz, 1H, SCH=), 5.03 (t, $^{3}J_{H,H}$ = 7.9 Hz, 1H, SCHSe), 3.06 (dd, $^{2}J_{H,H}$ = 12.8 Hz, $^{3}J_{H,H}$ = 7.9 Hz, 1H, $CHC\underline{H}_2Se$), 2.95 (dd, $^{2}J_{H,H}$ = 12.8 Hz, $^{3}J_{H,H}$ = 7.9 Hz, 1H, $CHC\underline{H}_2Se$), 2.65 (t, $^{3}J_{H,H}$ 7.2 = Hz, 2H, $SeC\underline{H}_2CH_2CH_3$), 1.69 (m, 2H, $C\underline{H}_2CH_3$), 0.99 (t, $^{3}J_{H,H}$ = 7.2 Hz, 3H, CH_3). ^{13}C{^{1}H} NMR (100 MHz, $CDCl_3$), δ (ppm): 119.56 (SCH=), 113.19 ($^{1}J_{Se,C}$ = 106.7 Hz, SeCH=), 48.59 ($^{1}J_{Se,C}$ = 66.9 Hz, $^{2}J_{Se,C}$ = 5.4 Hz, SCHSe), 33.73 ($^{1}J_{Se,C}$ = 67.7 Hz, $^{2}J_{Se,C}$ = 6.9 Hz, $CH\underline{C}H_2Se$), 27.50 ($^{1}J_{Se,C}$ = 60.9 Hz, $Se\underline{C}H_2CH_2CH_3$), 24.00 ($\underline{C}H_2CH_3$, $^{2}J_{Se,H}$ = 8.2 Hz), 14.45 (CH_3). ^{77}Se{^{1}H} NMR (76.3 MHz, $CDCl_3$), δ (ppm): 526.82 (SCHSe), 196.58 ($SeCH_2CH_2CH_3$). MS (EI): *m/z* (%) = 288 (14, M^+), 165 (8), 151 (100), 107 (6), 85 (32), 84 (24), 59 (18), 58 (26), 41 (42). Anal. Calcd for $C_7H_{12}SSe_2$: C, 29.38; H, 4.23; S, 11.21; Se, 55.19. Found: C, 29.71; H, 4.21; S, 11.41; Se, 55.45.

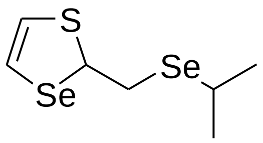

Isopropyl 1,3-thiaselenol-2-ylmethyl selenide (6d): Compound **6d** was obtained as light yellow oil using 2-iodopropane (187 mg, 1.1 mmol). Yield: 229 mg (80%) (from selenocyanate **4**) and 223 mg (78%) (from diselenide **8**). ^{1}H NMR (400 MHz, $CDCl_3$), δ (ppm): 6.63 (d, $^{3}J_{H,H}$ = 6.4 Hz, $^{2}J_{Se,H}$ = 48.1 Hz, 1H, SeCH=), 6.42 (d, $^{3}J_{H,H}$ = 6.4 Hz, 1H, SCH=), 5.05 (t, $^{3}J_{H,H}$ = 7.8 Hz, 1H, SCHSe), 3.26 (septet, $^{3}J_{H,H}$ = 6.8 Hz, 1H, $(C\underline{H}(CH_3)_2)$), 3.12 (dd, $^{2}J_{H,H}$ = 12.9 Hz, $^{3}J_{H,H}$ = 7.8 Hz, 1H,

SeCH(S)C$\underline{H}_2$Se), 3.01 (dd, $^2J_{H,H}$ = 12.9 Hz, $^3J_{H,H}$ = 7.8 Hz, 1H, SeCH(S)C$\underline{H}_2$Se), 1.43 (d, $^3J_{H,H}$ = 6.9 Hz, 6H, CH_3). $^{13}C\{^1H\}$ NMR (100 MHz, $CDCl_3$), δ (ppm): 119.54 (SCH=), 113.20 (SeCH=), 48.76 ($^1J_{Se,C}$ = 66.7 Hz, SCHSe), 32.67 (SeCH(S)$\underline{C}H_2$Se), 30.38 ($\underline{C}H(CH_3)_2$), 24.68 (CH_3). HMBC (^1H-^{77}Se) NMR (76.3 MHz, $CDCl_3$), δ (ppm): 327.08 ($SeCH_2(CH_3)_2$), 527.04 (SCHSe). MS (EI): *m/z* (%) = 288 (18, $[M]^+$), 165 (12), 151 (100), 107 (9), 85 (27), 84 (18), 59 (20), 58 (24), 41 (45). Anal. Calcd for $C_7H_{12}SSe_2$: C, 29.38; H, 4.23; S, 11.21; Se, 55.19. Found: C, 29.41; H, 4.03; S, 11.24; Se, 55.02.

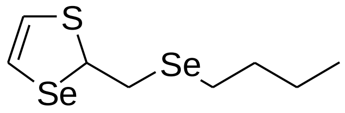

Butyl 1,3-thiaselenol-2-ylmethyl selenide (6e): Compound **6e** was obtained as light yellow oil using 1-bromobutane (151 mg, 1.1 mmol). Yield: 261 mg (87%) (from selenocyanate **4**) and 252 mg (85%) (from diselenide **8**). ^1H NMR (400 MHz, $CDCl_3$), δ (ppm): 6.64 (d, $^3J_{H,H}$ = 6.3 Hz, $^2J_{Se,H}$ = 48.3 Hz, 1H, SeCH=), 6.41 (d, $^3J_{H,H}$ = 6.3 Hz, 1H, SCH=), 5.04 (t, $^3J_{H,H}$ = 7.9 Hz, 1H, SCHSe), 3.07 (dd, $^2J_{H,H}$ = 12.9 Hz, $^3J_{H,H}$ = 7.9 Hz, 1H, CHC$\underline{H}_2$Se), 2.96 (dd, $^2J_{H,H}$ = 12.9 Hz, $^3J_{H,H}$ = 7.9 Hz, 1H, CHC$\underline{H}_2$Se), 2.67 (t, $^3J_{H,H}$ = 7.4 Hz, 2H, SeC$\underline{H}_2CH_2CH_2CH_3$), 1.65 (m, 2H, C$\underline{H}_2CH_2CH_3$), 1.41 (m, 2H, $CH_2C\underline{H}_2CH_3$), 0.92 (t, $^3J_{H,H}$ = 7.4 Hz, 3H, CH_3). $^{13}C\{^1H\}$ NMR (100 MHz, $CDCl_3$), δ (ppm): 119.55 (SCH=), 113.21 ($^1J_{Se,C}$ = 106.7 Hz, SeCH=), 48.57 ($^1J_{Se,C}$ = 66.9 Hz, SCHSe), 33.73 ($^1J_{Se,C}$ = 67.3 Hz, CH$\underline{C}H_2$Se), 32.76 ($\underline{C}H_2CH_2CH_3$), 25.04 ($^1J_{Se,C}$ = 60.4 Hz, Se$\underline{C}H_2CH_2CH_2CH_3$), 22.94 ($CH_2\underline{C}H_2CH_3$), 13.56 ($CH_3$). $^{77}Se\{^1H\}$ NMR (76.3 MHz, $CDCl_3$), δ (ppm): 526.72 (SCHSe), 199.85 ($SeCH_2CH_2CH_2CH_3$). MS (EI): *m/z* (%) = 302 (14, M^+), 165 (10), 151 (100), 107 (6), 85 (30), 84 (20), 71 (4), 59 (16), 58 (18), 45 (16). Anal. Calcd for $C_8H_{14}SSe_2$: C, 32.01; H, 4.70; S, 10.68; Se, 52.61. Found: C, 32.32; H, 4.58; S, 10.45; Se, 52.39.

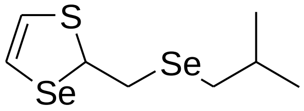

Isobutyl 1,3-thiaselenol-2-ylmethyl selenide (6f): Compound **6f** was obtained as light yellow oil using 1-bromo-2-methylpropane (151 mg, 1.1 mmol). Yield: 255 mg (85%) (from selenocyanate **4**) and 243 mg (81%) (from diselenide

8). ^{1}H NMR (400 MHz, $CDCl_3$), δ (ppm): 6.61 (d, $^{3}J_{H,H}$ = 6.3 Hz, $^{2}J_{Se,H}$ = 48.2 Hz, 1H, SeCH=), 6.41 (d, $^{3}J_{H,H}$ = 6.3 Hz, 1H, SCH=), 5.03 (t, $^{3}J_{H,H}$ = 7.9 Hz, 1H, SCHSe), 3.05 (dd, $^{2}J_{H,H}$ = 12.8 Hz, $^{3}J_{H,H}$ = 7.9 Hz, 1H, SeCH(S)C$\underline{H}_2$Se), 2.95 (dd, $^{2}J_{H,H}$ = 12.8 Hz, $^{3}J_{H,H}$ = 7.9 Hz, 1H, SeCH(S)C$\underline{H}_2$Se), 2.59 (d, $^{3}J_{H,H}$ = 6.8 Hz, 2H, C$\underline{H}_2$CH(CH_3)$_2$), 1.83 (tseptet, $^{3}J_{H,H}$ (H_3CCH) = 6.6 Hz, $^{3}J_{H,H}$ (H_2CCH) = 6.8 Hz, 1H, C$\underline{H}$(CH_3)$_2$), 1.00 (d, $^{3}J_{H,H}$ = 6.6 Hz, 6H, CH_3). ^{13}C{^{1}H} NMR (100 MHz, $CDCl_3$), δ (ppm): 119.54 (SCH=), 113.19 ($^{1}J_{Se,C}$ = 106.9 Hz, SeCH=), 48.53 ($^{1}J_{Se,C}$ = 66.7 Hz, SCHSe), 35.36 ($^{1}J_{Se,C}$ = 62.7 Hz, Se$\underline{C}H_2$CH(CH_3)$_2$), 34.34 ($^{1}J_{Se,C}$ = 67.3 Hz, SeCH(S)$\underline{C}H_2$Se), 29.42 ($\underline{C}$H(CH_3)$_2$), 22.58 (CH_3). ^{77}Se{^{1}H} NMR (76.3 MHz, $CDCl_3$), δ (ppm): 526.41 (SCHSe), 174.82 (SeCH_2CH(CH_3)$_2$). MS (EI): *m/z* (%) = 302 (12, M^+), 165 (8), 151 (100), 107 (6), 85 (29), 84 (25), 59 (16), 58 (30), 45 (16), 41 (50). Anal. Calcd for $C_8H_{14}SSe_2$: C 32.01; H 4.70; S 10.68; Se, 52.61. Found: C, 32.23; H, 4.68; S, 10.20; Se, 52.77.

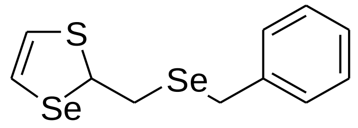

Benzyl 1,3-thiaselenol-2-ylmethyl selenide (6g): Compound **6g** was obtained as light yellow oil using 1-(bromomethyl)benzene (188 mg, 1.1 mmol). Yield: 307 mg (92%) (from selenocyanate **4**) and 291 mg (87%) (from diselenide **8**). ^{1}H NMR (400 MHz, $CDCl_3$), δ (ppm): 7.26-7.30 (m, 4H, H-2,3,5,6), 7.20-7.23 (m, 1H, H-4), 6.57 (d, $^{3}J_{H,H}$ = 6.4 Hz, $^{2}J_{Se,H}$ = 48.1 Hz, 1H, SeCH=), 6.36 (d, $^{3}J_{H,H}$ = 6.4 Hz, 1H, SCH=), 4.77 (t, $^{3}J_{H,H}$ = 7.8 Hz, 1H, SCHSe), 3.88 (s, $^{2}J_{Se,H}$ = 13.6 Hz, 2H, C$\underline{H}_2$Ph), 2.99 (dd, $^{2}J_{H,H}$ = 13.0 Hz, $^{3}J_{H,H}$ = 7.8 Hz, 1 H, CHC$\underline{H}_2$Se), 2.88 (dd, $^{2}J_{H,H}$ = 13.0 Hz, $^{3}J_{H,H}$ = 7.8 Hz, 1H, CHC$\underline{H}_2$Se). ^{13}C{^{1}H} NMR (100 MHz, $CDCl_3$), δ (ppm): 138.94 (C-1), 128.91 (C-3,5), 128.64 (C-2,6), 127.00 (C-4), 119.48 (SCH=), 113.19 ($^{1}J_{Se,C}$ = 106.6 Hz, SeCH=), 48.25 ($^{1}J_{Se,C}$ = 67.2 Hz, $^{2}J_{Se,C}$ = 5.5 Hz, SCHSe), 33.54 ($^{1}J_{Se,C}$ = 69.3 Hz, $^{2}J_{Se,C}$ = 7.0 Hz, CH$\underline{C}H_2$Se), 28.27 ($^{1}J_{Se,C}$ = 60.2 Hz, *C*H_2Ph). ^{77}Se{^{1}H} NMR (76.3 MHz, $CDCl_3$), δ (ppm): 527.86 (SCHSe), 293.46 (SeCH_2Ph). MS (EI): *m/z* (%) = 336 (12, M^+), 165 (6), 151 (59), 107 (3), 91 (100), 85 (12), 84 (9), 59 (5), 58 (4). Anal. Calcd for $C_{11}H_{12}SSe_2$: C, 39.53; H, 3.62; S, 9.59; Se 47.25. Found: C 39.19; H 3.52; S 9.57; Se 47.93.

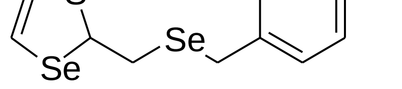

4-Fluorobenzyl 1,3-thiaselenol-2-ylmethyl selenide (6h): Compound **6h** was obtained as light yellow oil using 1-(chloromethyl)-4-fluorobenzene (159 mg, 1.1 mmol). Yield: 310 mg (88%) (from selenocyanate **4**) and 299 mg (85%) (from diselenide **8**). ^{1}H NMR (400 MHz, $CDCl_3$), δ (ppm): 7.23 (dd, $^{3}J_{H,H}$ = 8.7 Hz, $^{4}J_{F,H}$ = 5.4 Hz, 2H, H-2,6), 6.97 (t, $^{3}J_{H,H}$ = 8.7 Hz, $^{3}J_{F,H}$ = 8.7 Hz, 2H, H-3,5), 6.58 (d, $^{3}J_{H,H}$ = 6.3 Hz, $^{2}J_{Se,H}$ = 48.2 Hz, 1H, SeCH=), 6.37 (d, $^{3}J_{H,H}$ = 6.3 Hz, 1H, SCH=), 4.84 (dd, $^{3}J_{H,H}$ = 7.6 Hz, $^{3}J_{H,H}$ = 7.9 Hz, 1H, SCHSe), 3.86 (s, $^{2}J_{Se,H}$ = 13.2 Hz, 2H, $C\underline{H}_2Ar$), 2.97 (dd, $^{2}J_{H,H}$ = 13.0 Hz, $^{3}J_{H,H}$ = 7.9 Hz, 1H, $CHC\underline{H}_2Se$), 2.86 (dd, $^{2}J_{H,H}$ = 13.0 Hz, $^{3}J_{H,H}$ = 7.6 Hz, 1H, $CHC\underline{H}_2Se$). ^{13}C{^{1}H} NMR (100 MHz, $CDCl_3$), δ (ppm): 161.72 ($^{1}J_{F,C}$ = 246.5 Hz, C-4), 134.63 ($^{4}J_{F,C}$ = 3.4 Hz, C-1), 130.39 ($^{3}J_{F,C}$ = 8.1 Hz, C-2,6), 119.45 (SCH=), 115.47 ($^{2}J_{F,C}$ = 21.5 Hz, C-3,5), 113.23 ($^{1}J_{Se,C}$ = 106.5 Hz, SeCH=), 48.25 ($^{1}J_{Se,C}$ = 67.4 Hz, $^{2}J_{Se,C}$ = 5.5 Hz, SCHSe), 33.49 ($^{1}J_{Se,C}$ = 69.3 Hz, $^{2}J_{Se,C}$ = 7.1 Hz, $CH\underline{C}H_2Se$), 27.48 ($^{1}J_{Se,C}$ = 58.8 Hz, $\underline{C}H_2Ar$). ^{19}F NMR (376 MHz, $CDCl_3$), δ (ppm): - 114.95 (tt, $^{3}J_{F,H}$ = 8.7 Hz, $^{4}J_{F,H}$ = 5.4 Hz). ^{77}Se{^{1}H} NMR (76.3 MHz, $CDCl_3$), δ (ppm): 528.85 (SCHSe), 293.03 ($SeCH_2Ar$). MS (EI): *m/z* (%) = 354 (18, M^+), 245 (8), 165 (22), 151 (100), 107 (11), 85 (20), 84 (115), 59 (21), 58 (20). Anal. Calcd for $C_{11}H_{11}FSSe_2$: C, 37.51; H, 3.15; S, 9.10; Se, 44.84. Found: C, 37.69; H, 3.15; S, 8.70; Se, 44.50.

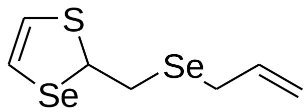

Allyl 1,3-thiaselenol-2-ylmethyl selenide (6i): Compound **6i** was obtained as light yellow oil using 3-bromo-1-propene (133 mg, 1.1 mmol). Yield: 256 mg (90%) (from selenocyanate **4**) and 242 mg (85%) (from diselenide **8**). ^{1}H NMR (400 MHz, $CDCl_3$), δ (ppm): 6.61 (d, $^{3}J_{H,H}$ = 6.3 Hz, $^{2}J_{Se,H}$ = 47.9 Hz, 1H, SeCH=), 6.40 (d, $^{3}J_{H,H}$ = 6.3 Hz, 1H, SCH=), 5.86 (ddt, 1H, CH_2CH=, $^{3}J_{cis}$ = 9.6 Hz, $^{3}J_{trans}$ = 17.1 Hz, $^{3}J_{H,H}$ = 7.6 Hz), 5.06 (d, $^{3}J_{trans}$ = 17.1 Hz, 1H, =CH_2), 5.04 (d, $^{3}J_{cis}$ = 9.6 Hz, 1H, =CH_2), 5.01 (dd, $^{3}J_{H,H}$ = 7.6 Hz, $^{3}J_{H,H}$ = 8.1 Hz, 1H, SCHSe), 3.28 (d, $^{3}J_{H,H}$ = 7.6 Hz, 2H, $SeC\underline{H}_2CH$=), 3.02 (dd, $^{2}J_{H,H}$ = 13.0 Hz, $^{3}J_{H,H}$ = 7.6 Hz, 1H, $SeCH(S)C\underline{H}_2Se$), 2.91 (dd, $^{2}J_{H,H}$ = 13.0 Hz, $^{3}J_{H,H}$ = 7.6 Hz, 1H, $SeCH(S)C\underline{H}_2Se$). ^{13}C{^{1}H} NMR (100 MHz, $CDCl_3$), δ (ppm): 134,74 ($CH_2\underline{C}H$=),119.41 ($^{2}J_{Se,C}$ = 35.0 Hz, SCH=), 116.74 (=CH_2), 113.18 ($^{1}J_{Se,C}$ = 107.3 Hz, SeCH=),

48.27 ($^{1}J_{Se,C}$ = 67.7 Hz, SCHSe), 32.82 ($^{1}J_{Se,C}$ = 69.6 Hz, SeCH(S)$\underline{C}H_2$Se), 27.02 ($^{1}J_{Se,C}$ = 57.8 Hz, $\underline{C}H_2CH=CH_2$). ^{77}Se{^{1}H} NMR (76.3 MHz, $CDCl_3$), δ (ppm): 527.40 (SCHSe), 228.82 ($SeCH_2CH=CH_2$). MS (EI): *m/z* (%) = 286 (15, M^+), 245 (17), 165 (36), 151 (100), 107 (9), 85 (73), 84 (40), 59 (32), 58 (34), 41 (48). Anal. Calcd for $C_7H_{10}SSe_2$: C, 29.59; H, 3.55; S, 11.29; Se, 55.58. Found: C, 29.36; H, 3.76; S, 11.07; Se, 55.61.

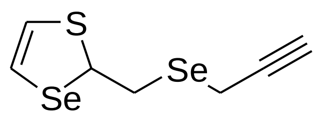

2-Propynyl 1,3-thiaselenol-2-ylmethyl selenide (6j): Compound **6j** was obtained as light yellow oil using 3-bromo-1-propyne (131 mg, 1.1 mmol). Yield: 243 mg (86%) (from selenocyanate **4**) and 229 mg (81%) (from diselenide **8**). ^{1}H NMR (400 MHz, $CDCl_3$), δ (ppm): 6.62 (d, $^{3}J_{H,H}$ = 6.3 Hz, $^{2}J_{Se,H}$ = 48.2 Hz, 1H, SeCH=), 6.41 (d, $^{3}J_{H,H}$ = 6.3 Hz, 1H, SCH=), 5.13 (t, $^{3}J_{H,H}$ = 7.8 Hz, 1H, SCHSe), 3.30 (d, $^{4}J_{H,H}$ = 2.7 Hz, 2H, $CH_2C≡$),3.28 (dd, $^{2}J_{H,H}$ = 13.1 Hz, $^{3}J_{H,H}$ = 7.8 Hz, 1H, SeCH(S)$C\underline{H}_2$Se), 3.17 (dd, $^{2}J_{H,H}$ = 13.1 Hz, $^{3}J_{H,H}$ = 7.8 Hz, 1H, SeCH(S)$C\underline{H}_2$Se), 2.29 (t, $^{4}J_{H,H}$ = 2.7 Hz, 1H, ≡CH). ^{13}C{^{1}H} NMR (100 MHz, $CDCl_3$), δ (ppm): 119.43 (SCH=), 113.26 ($^{1}J_{Se,C}$ = 106.3 Hz, SeCH=), 80.67 ($CH_2\underline{C}≡$),71.81 (≡CH), 47.93 ($^{1}J_{Se,C}$ = 67.4 Hz, SCHSe), 36.23 ($^{1}J_{Se,C}$ = 68.0 Hz, SeCH(S)$\underline{C}H_2$Se), 7.95 ($^{1}J_{Se,C}$ = 62.0 Hz, $\underline{C}H_2C≡$). ^{77}Se{^{1}H} NMR (76.3 MHz, $CDCl_3$), δ (ppm): 528.38 (SCHSe), 285.49 ($SeCH_2C≡CH$). MS (EI): *m/z* (%) = 284 (8, M^+), 243 (8), 165 (8), 151 (100), 107 (9), 85 (33), 84 (21), 59 (25), 58 (31), 45 (39). Anal. Calcd for $C_7H_8SSe_2$: C, 29.80; H, 2.86; S 11.37; Se, 55.96: Found: C 29.52; H 2.78; S 11.40; Se, 55.87.

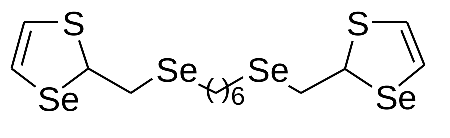

1,3-Thiaselenol-2-ylmethyl 6-[(1,3-thiaselenol-2-ylmethyl)selanyl]hexyl selenide (6k): A solution of 1,6-dibromohexane (268 mg, 1.1 mmol) in MeOH (1 mL) was added to a solution of selenocyanate **4** (538 mg, 2 mmol) in MeOH (1 mL) under argon. Then $NaBH_4$ (174 mg, 4.6 mmol) was added portionwise to the stirred resulting mixture. The mixture was stirred for 1 h at room temperature under argon. The mixture was diluted with cold degassed water (15 mL) and extracted with $CHCl_3$ (3 × 10 mL). The organic phase was dried

over $CaCl_2$ and the solvent was removed by rotary evaporation. Product **6k** was isolated by column chromatography (silica gel, eluent: hexane → hexane/$CHCl_3$ 3:1) as light yellow oil. Yield: 428 mg (75%). ^{1}H NMR (400 MHz, $CDCl_3$), δ (ppm): 6.62 (d, $^3J_{H,H}$ = 6.3 Hz, $^2J_{Se,H}$ = 48.2 Hz, 1H, SeCH=), 6.41 (d, $^3J_{H,H}$ = 6.3 Hz, 1H, SCH=), 5.04 (t, $^3J_{H,H}$ = 7.8 Hz, 1H, SCHSe), 3.07 (dd, $^2J_{H,H}$ = 12.8 Hz, $^3J_{H,H}$ = 7.8 Hz, 1H, $SeCH(S)C\underline{H}_2Se$), 2.96 (dd, $^2J_{H,H}$ = 12.8 Hz, $^3J_{H,H}$ = 7.8 Hz, 1H, $SeCH(S)C\underline{H}_2Se$), 2.67 (t, $^3J_{H,H}$ = 7.4 Hz, 4H, CH_2-3), 1.67 (m, 4H, CH_2-2), 1.41 (m, 4H, CH_2-1). $^{13}C\{^1H\}$ NMR (100 MHz, $CDCl_3$), δ (ppm): 119.58 (SCH=), 113.23 ($^1J_{Se,C}$ = 106.7 Hz, SeCH=), 48.58 ($^1J_{Se,C}$ = 67.2 Hz, SCHSe), 33.81 ($^1J_{Se,C}$ = 67.9 Hz, $SeCH(S)\underline{C}H_2Se$), 30.47 ($CH_2$-2), 29.22 ($CH_2$-3), 25.24 ($^1J_{Se,C}$ = 60.6 Hz, CH_2-1). $^{77}Se\{^1H\}$ NMR (76.3 MHz, $CDCl_3$), δ (ppm): 527.02 (SCHSe), 199.74 ($Se(CH_2)_6Se$). MS (EI): *m/z* (%) = 570 (3, M^+), 245 (8), 165 (10), 151 (100), 107 (2), 85 (21), 84 (10), 59 (10), 58 (24), 41 (45). Anal. Calcd for $C_{14}H_{22}S_2Se_4$: C, 29.48; H, 3.89; S 11.25; Se, 55.38. Found: C 29.40; H 3.87; S 11.40; Se, 55.71.

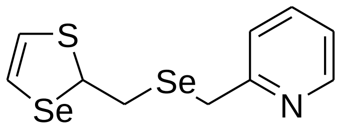

2-Pyridinylmethyl 1,3-thiaselenol-2-ylmethyl selenide (6l): A solution of 85% NaOH (47 mg, 1 mmol) in MeOH (1 mL) was added to a solution of 2-(chloromethyl)pyridinium chloride (164 mg, 1 mmol) in MeOH (1 mL) and mixture was stirred for 10 min at room temperature. A solution of selenocyanate **4** (269 mg, 1 mmol) in MeOH (1 mL) was added followed by portionwise addition of $NaBH_4$ (87 mg, 2.3 mmol) under argon. The mixture was stirred for 1 h at room temperature under argon. The mixture was diluted with cold degassed water (15 mL) and extracted with $CHCl_3$ (3 × 10 mL). The organic phase was dried over $CaCl_2$ and the solvent was removed by rotary evaporation. Product **6l** was isolated by column chromatography (silica gel, eluent: hexane → hexane/$CHCl_3$ 1:1) as light yellow oil. Yield: 267 mg (84%). ^{1}H NMR (400 MHz, $CDCl_3$), δ (ppm): 8.48 (ddd, $^3J_{H-5,H-6}$ = 4.8 Hz, $^4J_{H-4,H-6}$ = 1.9 Hz, $^5J_{H-3,H-6}$ = 1.1 Hz, 1H, H-6), 7.61 (td, $^3J_{H-3,H-4}$ = 7.5 Hz, $^3J_{H-4,H-5}$ = 7.5 Hz, $^4J_{H-4,H-6}$ = 1.9 Hz, 1H, H-4), 7.28 (ddd, $^3J_{H-3,H-4}$ = 7.5 Hz, $^4J_{H-3,H-5}$ = 1.3 Hz, $^5J_{H-3,H-6}$ = 1.1 Hz, 1H, H-3), 7.12 (ddd, $^3J_{H-5,H-6}$ = 4.8 Hz, $^3J_{H-4,H-5}$ = 7.5 Hz, $^4J_{H-3,H-5}$ = 1.3 Hz, 1H, H-5), 6.56

(d, $^3J_{H,H}$ = 6.3 Hz, $^2J_{Se,H}$ = 48.2 Hz, 1H, SeCH=), 6.35 (d, $^3J_{H,H}$ = 6.3 Hz, 1H, SCH=), 4.91 (dd, $^3J_{H,H}$ = 7.6 Hz, $^3J_{H,H}$ = 7.9 Hz, 1H, SCHSe), 3.95 (s, $^2J_{Se,H}$ = 14.4 Hz, 2H, pyCH_2), 3.09 (dd, $^2J_{H,H}$ = 13.0 Hz, $^3J_{H,H}$ = 7.9 Hz, 1H, $SeCH(S)C\underline{H}_2Se$), 2.99 (dd, $^2J_{H,H}$ = 13.0 Hz, $^3J_{H,H}$ = 7.6 Hz, 1H, $SeCH(S)C\underline{H}_2Se$). $^{13}C\{^1H\}$ NMR (100 MHz, $CDCl_3$), δ (ppm): 159.47 (C-2), 149.16 (C-6), 136.68 (C-4), 122.91 (C-3), 121.65 (C-5), 119.38 (SCH=), 113.13 ($^1J_{Se,C}$ = 106.6 Hz, SeCH=), 48.02 ($^1J_{Se,C}$ = 67.1 Hz, $^2J_{Se,C}$ = 4.9 Hz, SCHSe), 33.76 ($^1J_{Se,C}$ = 67.9 Hz, $^2J_{Se,C}$ = 4.9 Hz, $SeCH(S)\underline{C}H_2Se$), 29.28 ($^1J_{Se,C}$ = 62.3 Hz, pyCH_2). HMBC (^1H-^{15}N) NMR (40 MHz, $CDCl_3$), δ (ppm): -70.40. $^{77}Se\{^1H\}$ NMR (76.3 MHz, $CDCl_3$), δ (ppm): 527.54 (SCHSe), 289.61 ($SeCH_2$py). MS (EI): *m/z* (%) = 337 (11, M^+), 165 (20), 151 (100), 107 (15), 85 (24), 84 (11), 59 (22), 58 (14). Anal. Calcd for $C_{10}H_{11}NSSe_2$: C, 35.83; H, 3.31; N, 4.18; S 9.57; Se, 47.11. Found: C 35.93; H 3.44; N, 4.11; S 9.28; Se, 47.15.

General procedure for the synthesis of organyl (*Z*)/(*E*)-3-[(1,3-thiaselenol-2-ylmethyl)selanyl]-2-propenoate (7a,b): A solution of alkyl propiolate (1 mmol) in MeOH (2 mL) was added to a solution of selenocyanate **4** (1 mmol) in MeOH (3 mL) and the mixture was cooled to ≈ 0 °C in an ice bath. Then $NaBH_4$ (2.3 mmol) was added portionwise to the stirred resulting mixture under argon and the mixture was stirred for 1 h at ≈ 0 °C under argon and allowed to warm to room temperature. The mixture was diluted with cold degassed water (15 mL) and extracted with $CHCl_3$ (3 × 15 mL). The organic phase was dried over $CaCl_2$ and the solvent was removed in vacuum giving compounds **7a** or **7b** as light yellow oils.

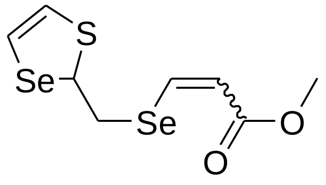

Methyl 3-[(1,3-thiaselenol-2-ylmethyl)selanyl]-2-propenoate (7a): Compound **7a** (*Z*/*E* = 94:6) was obtained as light yellow oil using selenocyanate **4** (269 mg, 1 mmol), methyl propiolate (84 mg, 1 mmol) and $NaBH_4$ (87 mg, 2.3 mmol). Yield: 308 mg (94%). Anal. Calcd for $C_8H_{10}O_2SSe_2$: C, 29.28; H, 3.07; Se, 48.12. Found: C, 29.57; H, 2.70; Se, 48.28.

(Z)-Methyl 3-[(1,3-thiaselenol-2-ylmethyl)selanyl]-2-propenoate. ^{1}H NMR (400 MHz, $CDCl_3$), δ (ppm): 7.65 (d, $^3J_{cis}$ = 9.6 Hz, 1H, SeCH=C<u>H</u>C(O)), 6.61 (d, $^3J_{H,H}$ = 6.3 Hz, $^2J_{Se,H}$ = 48.4 Hz, 1H, SeCH=), 6.39 (d, $^3J_{H,H}$ = 6.3 Hz, 1 H, SCH=), 6.31 (d, $^3J_{cis}$ = 9.6 Hz, $^2J_{Se,H}$ = 9.5 Hz, 1H, SeC<u>H</u>=CHC=O), 5.01 (dd, $^3J_{H,H}$ = 7.5 Hz, $^3J_{H,H}$ = 7.8 Hz, 1H, SCHSe), 3.72 (s, 3 H, OCH_3), 3.19 (dd, $^2J_{HH}$ = 13.0 Hz, $^3J_{H,H}$ = 7.8 Hz, 1H, CH_2Se), 3.10 (dd, $^2J_{HH}$ = 13.0 Hz, $^3J_{H,H}$ = 7.5 Hz, 1H, CH_2Se). ^{13}C{^{1}H} NMR (100 MHz, $CDCl_3$), δ (ppm): 167.41 (C=O), 148.08 ($^1J_{Se,C}$ = 137.5 Hz, Se<u>C</u>H=CHC=O), 119.36 (SCH=), 116.75 (SeCH=<u>C</u>HC(O)), 113.38 ($^1J_{Se,C}$ = 106.6 Hz, SeCH=), 47.98 ($^1J_{Se,C}$ = 68.2 Hz, SCHSe), 51.39 (OCH_3), 38.41 ($^1J_{Se,C}$ = 63.4 Hz, $^2J_{Se,C}$ = 7.8 Hz, CH_2Se). ^{77}Se{^{1}H} NMR (76.3 MHz, $CDCl_3$), δ (ppm): 528.12 (SCHSe), 401.09 (SeCH=CHC=O).

(E)-Methyl 3-[(1,3-thiaselenol-2-ylmethyl)selanyl]-2-propenoate. ^{1}H NMR (400 MHz, $CDCl_3$), δ (ppm): 8.02 (d, $^3J_{trans}$ = 15.8 Hz, 1H, SeCH=C<u>H</u>C(O)), 6.05 (d, $^3J_{trans}$ = 15.8 Hz 1H, SeC<u>H</u>=CHC(O)), 5.11 (dd, $^3J_{H,H}$ = 7.5 Hz, $^3J_{H,H}$ = 7.8 Hz, 1H, SCHSe), 3.70 (s, 3H, OCH_3), (the remaining signals are masked by the signals of the major Z-isomer). ^{13}C{^{1}H} NMR (100 MHz, $CDCl_3$), δ (ppm): 164.78 (C=O), 142.67 (Se<u>C</u>H=CHC=O), 119.57 (SCH=), 116.75 (SeCH=<u>C</u>HC=O), 113.43 (SeCH=), 51.44 (OCH_3), 46.88 (SCHSe), 35.70 (CH_2Se). ^{77}Se{^{1}H} NMR (76.3 MHz, $CDCl_3$), δ (ppm): 534.48 (SCHSe), 322.91 (SeCH=CHC=O).

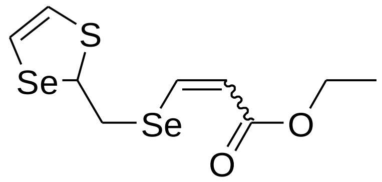

Ethyl 3-[(1,3-thiaselenol-2-ylmethyl)selanyl]-2-propenoate (7b): Compound **7b** (Z/E = 93:7) was obtained as light yellow oil using selenocyanate **4** (269 mg, 1 mmol), ethyl propiolate (98 mg, 1 mmol) and $NaBH_4$ (87 mg, 2.3 mmol). Yield: 308 mg (90%). Anal. Calcd for $C_9H_{12}O_2SSe_2$: C, 31.59; H, 3.53; Se, 46.15. Found: C, 31.82; H, 3.44; Se, 46.43.

(Z)-Ethyl 3-[(1,3-thiaselenol-2-ylmethyl)selanyl]-2-propenoate. ^{1}H NMR (400 MHz, $CDCl_3$), δ (ppm): 7.64 (d, $^3J_{cis}$ = 9.7 Hz, 1H, SeCH=C<u>H</u>C=O), 6.62 (d, $^3J_{H,H}$ = 6.5 Hz, $^2J_{Se,H}$ = 48.4 Hz, 1H, SeCH=), 6.41 (d, $^3J_{H,H}$ = 6.5 Hz, 1H, SCH=), 6.32 (d, $^3J_{cis}$ = 9.7 Hz, $^2J_{Se,H}$ = 9.7 Hz, 1H, SeC<u>H</u>=CHC=O), 5.03 (dd, $^3J_{H,H}$ = 7.5 Hz, $^3J_{H,H}$ = 7.9 Hz, 1H, SCHSe), 4.21 (q, $^3J_{H,H}$ = 7.1 Hz,

2H, OC$\underline{H}_2$CH$_3$), 3.21 (dd, $^2J_{HH}$ = 13.0 Hz, $^3J_{H,H}$ = 7.9 Hz, 1H, CH$_2$Se), 3.11 (dd, $^2J_{HH}$ = 13.0 Hz, $^3J_{H,H}$ = 7.5 Hz, 1H, CH$_2$Se), 1.29 (t, $^3J_{H,H}$ = 7.1 Hz, 3H, OCH$_2$C$\underline{H}_3$). ^{13}C{^{1}H} NMR (100 MHz, CDCl$_3$), δ (ppm): 167.15 (C=O), 147.66 ($^1J_{Se,C}$ = 138.7 Hz, Se$\underline{C}$H=CHC=O), 119.44 (SCH=), 117.33 (SeCH=$\underline{C}$HC=O), 113.40 ($^1J_{Se,C}$ = 105.7 Hz, SeCH=), 60.39 (O$\underline{C}$H$_2$CH$_3$), 48.06 ($^1J_{Se,C}$ = 67.4 Hz, SCHSe), 38.50 ($^1J_{Se,C}$ = 63.1 Hz, $^2J_{Se,C}$ = 6.4 Hz, CH$_2$Se), 14.24 (OCH$_2\underline{C}$H$_3$). ^{77}Se{^{1}H} NMR (76.3 MHz, CDCl$_3$), δ (ppm): 527.96 (SCHSe), 399.88 (SeCH=CHC=O).

(*E*)-Ethyl 3-[(1,3-thiaselenol-2-ylmethyl)selanyl]-2-propenoate. ^{1}H NMR (400 MHz, CDCl$_3$), δ (ppm): 8.02 (d, $^3J_{trans}$ = 15.6 Hz, 1H, SeCH=C$\underline{H}$C=O), 6.05 (d, $^3J_{trans}$ = 15.6 Hz 1H, SeC$\underline{H}$=CHC=O), 4.13 (q, $^3J_{H,H}$ = 7.1 Hz, 2H, OC$\underline{H}_2$CH$_3$), 1.28 (t, $^3J_{H,H}$ = 7.1 Hz, 3H, OCH$_2$C$\underline{H}_3$) (the remaining signals are masked by the signals of the major *Z*-isomer). ^{13}C{^{1}H} NMR (100 MHz, CDCl$_3$), δ (ppm): 164.48 (C=O), 142.15 (Se$\underline{C}$H=CHC=O), 119.38 (SCH=), 117.19 (SeCH=$\underline{C}$HC=O), 113.44 (SeCH=), 59.97 (O$\underline{C}$H$_2$CH$_3$), 46.95 (SCHSe), 35.68 (CH$_2$Se), 14.14 (OCH$_2\underline{C}$H$_3$). ^{77}Se{^{1}H} NMR (76.3 MHz, CDCl$_3$), δ (ppm): 534.32 (SCHSe), 321.02 (SeCH=CHC=O).

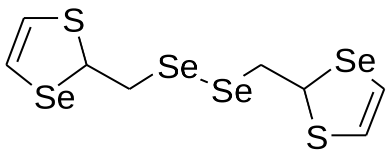

Bis(1,3-thiaselenol-2-ylmethyl) diselenide (8): Powdered selenium (158 mg, 2 mmol) was added to a solution of KCN (130 mg, 2 mmol) in MeOH (10 mL). The mixture was stirred at room temperature until the solid disappeared (usually ≈ 0.5 h). The solvent was removed by rotary evaporation and a solution of thiaselenole **1** (488 mg, 2.00 mmol) in MeCN (10 mL) was added to the residue and the mixture was stirred at room temperature for 1 h and filtered. The solvent was removed by rotary evaporation and the residue (compound **2**) was dissolved in MeOH (10 mL). A solution of 85% KOH (264 g, 4 mmol) in MeOH (10 mL) was added and the mixture was stirred overnight. The solvent was removed by rotary evaporation and the residue was extracted by chloroform (3 × 10 mL). The solvent was removed from the organic phase by rotary evaporation and the residue was dried in vacuum giving diselenide **8** as dark yellow oil. Yield: 875 mg (90%). ^{1}H NMR (400 MHz, CDCl$_3$), δ (ppm): 6.64 (d, $^3J_{H,H}$ = 6.3 Hz, $^2J_{Se,H}$ =

48.3 Hz, 1H, SeCH=), 6.43 (d, $^3J_{H,H}$ = 6.3 Hz, $^3J_{Se,H}$ = 9.9 Hz, 1H, SCH=), 5.15 (dd, $^3J_{H,H}$ = 7.3 Hz, $^3J_{H,H}$ = 7.9 Hz, 1H, SCHSe), 3.44 (dd, $^2J_{H,H}$ = 12.5 Hz, $^3J_{H,H}$ = 7.9 Hz, 1H, SeCH(S)C$\underline{H}_2$Se), 3.35 (dd, $^2J_{H,H}$ = 12.5 Hz, $^3J_{H,H}$ = 7.3 Hz, 1H, SeCH(S)C$\underline{H}_2$Se). $^{13}C\{^1H\}$ NMR (100 MHz, $CDCl_3$), δ (ppm): 119.68, 119.67 (d, SCH=), 113.53, 113.52 (d, $^1J_{Se,C}$ = 105.6 Hz, SeCH=), 47.97 ($^1J_{Se,C}$ = 67.2 Hz, SCHSe), 40.07 ($^1J_{Se,C}$ = 77.1 Hz, $^2J_{Se,C}$ = 6.3 Hz, SeCH(S)$\underline{C}H_2$Se). $^{77}Se\{^1H\}$ NMR (76.3 MHz, $CDCl_3$), δ (ppm): 527.70, 526.61 (SCHSe), 348.51 (SeSe). MS (EI): *m/z* (%) = 488 (10, M^+), 243 (14), 165 (100), 151 (26), 107 (9), 85 (96), 59 (31), 58 (41), 45 (26). Anal. Calcd for $C_8H_{10}S_2Se_4$: C, 19.77; H, 2.07; S 13.19; Se, 64.97. Found: C 20.02; H 2.09; S 13.06; Se, 64.65.

General procedure for the synthesis of organyl 1,3-thiaselenol-2-ylmethyl selenides 6a–j from diselenide 8: A solution of the organic halide (1.1 mmol) in MeOH (1 mL) was added to a solution of diselenide **8** (244 mg, 0.5 mmol) in MeOH (2 mL). Then $NaBH_4$ (49 mg, 1.3 mmol) was added portionwise to the stirred resulting mixture under argon. The mixture was stirred for 1 h at room temperature under argon, diluted with cold degassed water (15 mL) and extracted with $CHCl_3$ (3 × 10 mL). The organic phase was dried over $CaCl_2$ and the solvent was removed by rotary evaporation. The products **6a–j** were purified by column chromatography (silica gel, eluent: hexane → hexane/$CHCl_3$ 4:1).

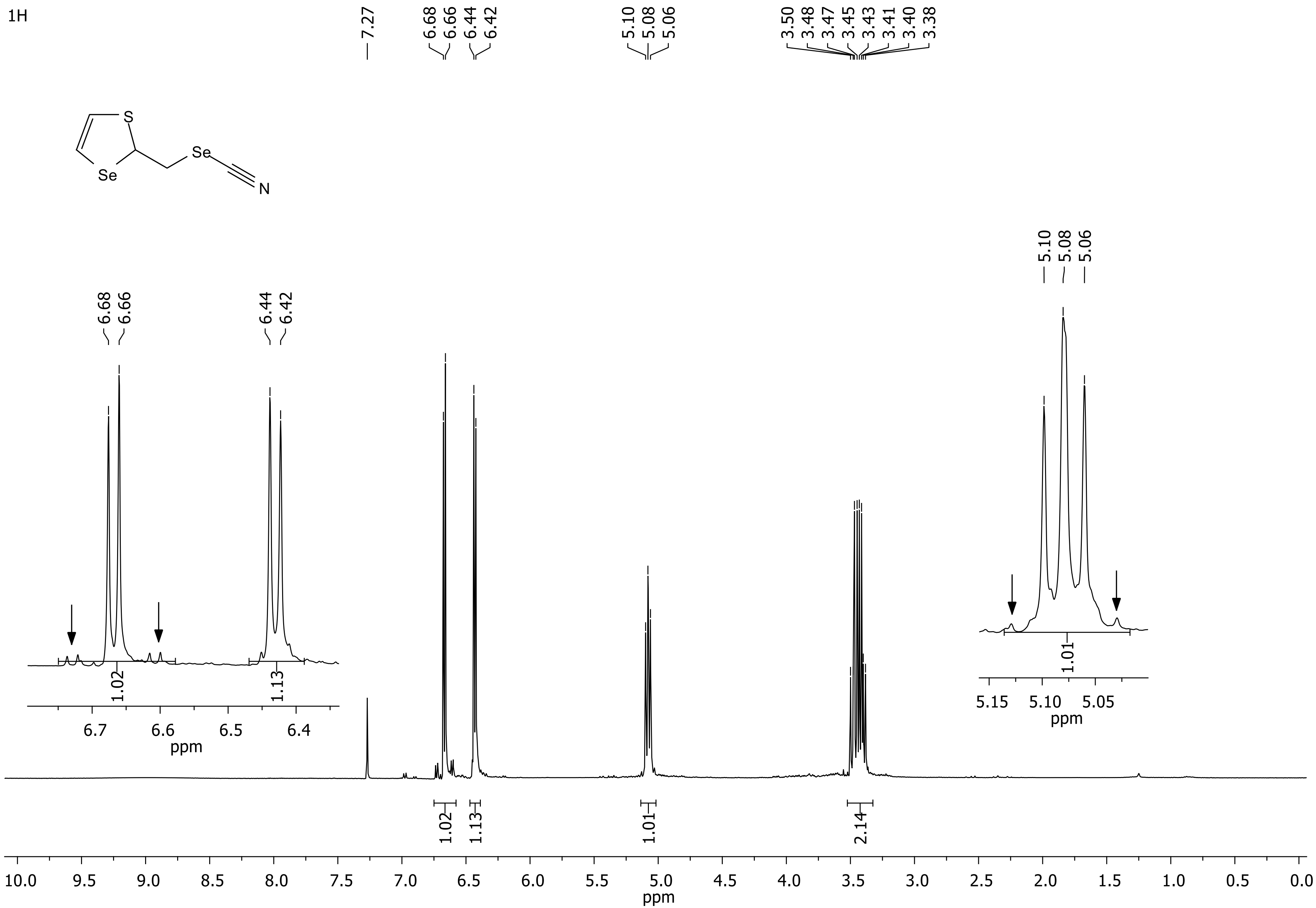

^{1}H NMR spectrum of 1,3-thiaselenol-2-ylmethyl selenocyanate (4)

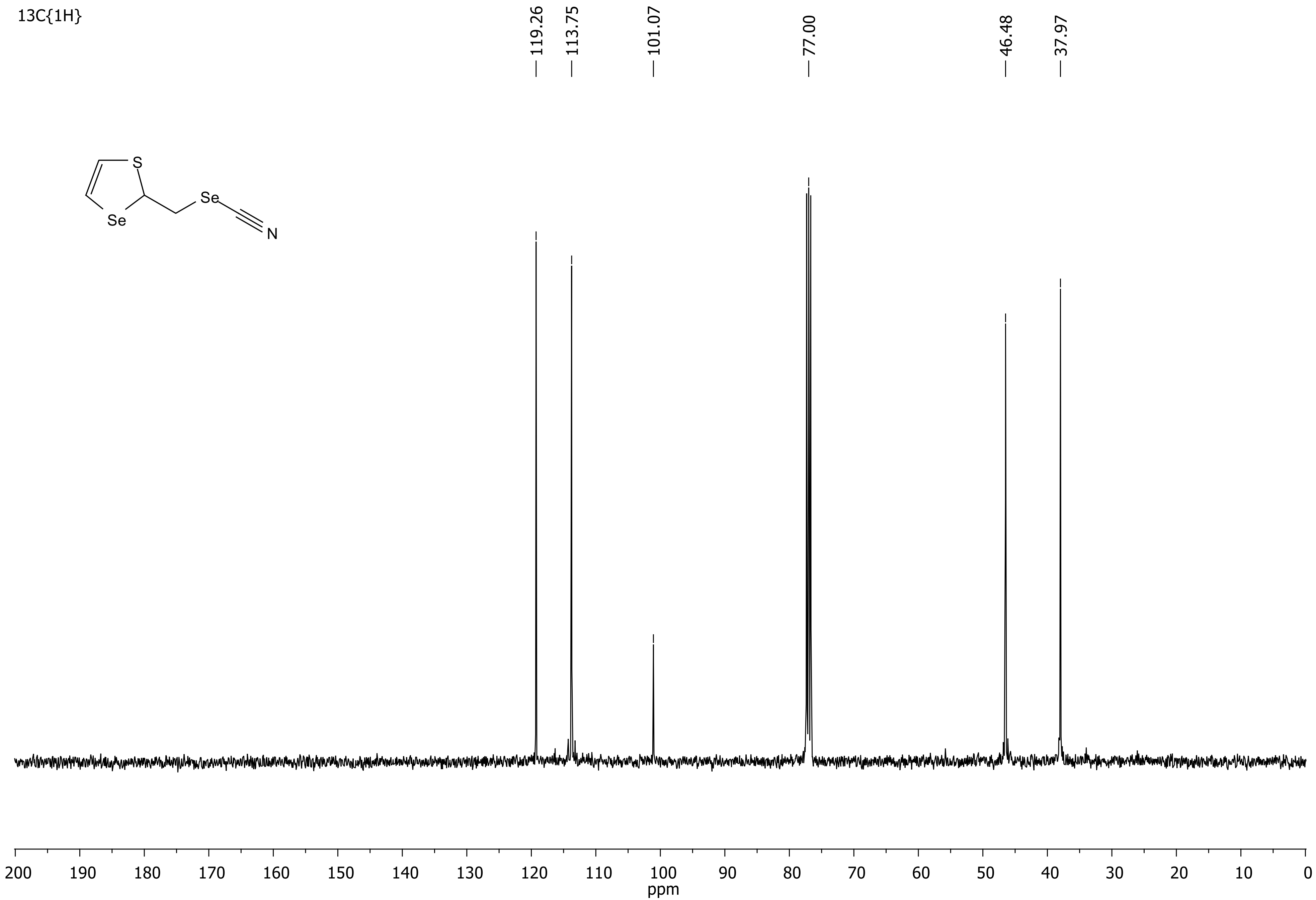

$^{13}C\{^{1}H\}$ NMR spectrum of 1,3-thiaselenol-2-ylmethyl selenocyanate (4)

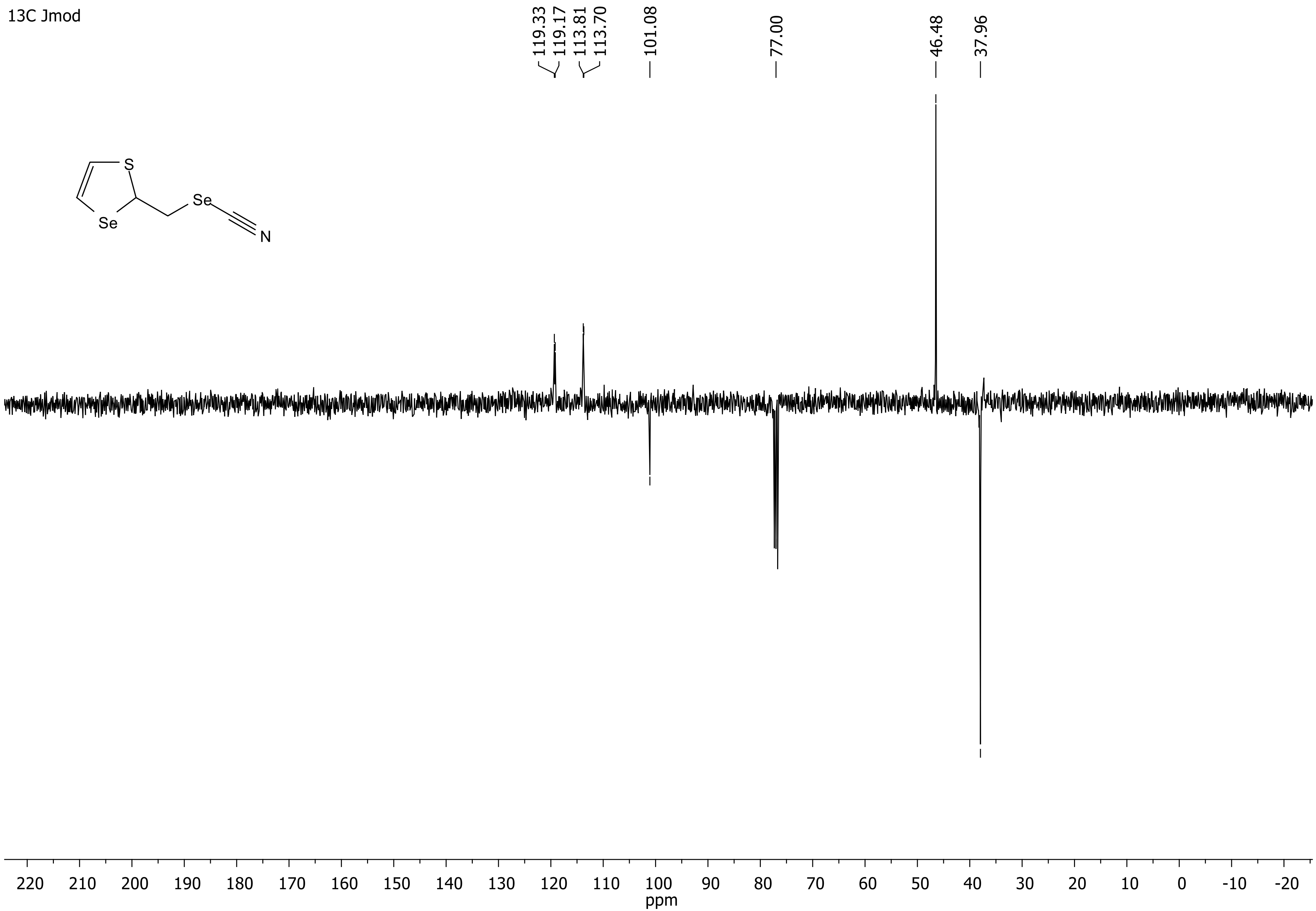

^{13}C NMR J$_{mod}$ spectrum of 1,3-thiaselenol-2-ylmethyl selenocyanate (4)

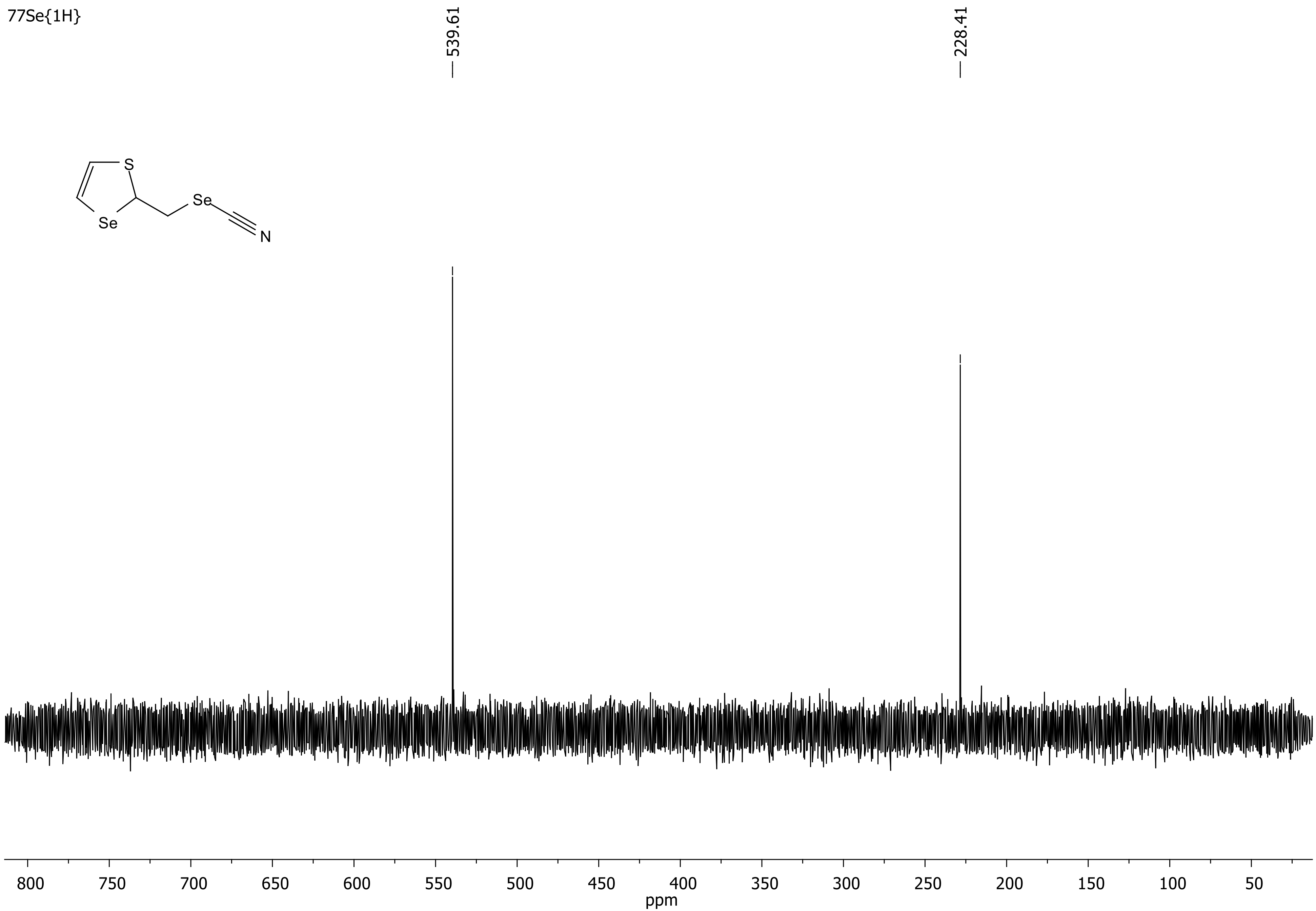

^{77}Se{^{1}H} NMR spectrum of 1,3-thiaselenol-2-ylmethyl selenocyanate (4)

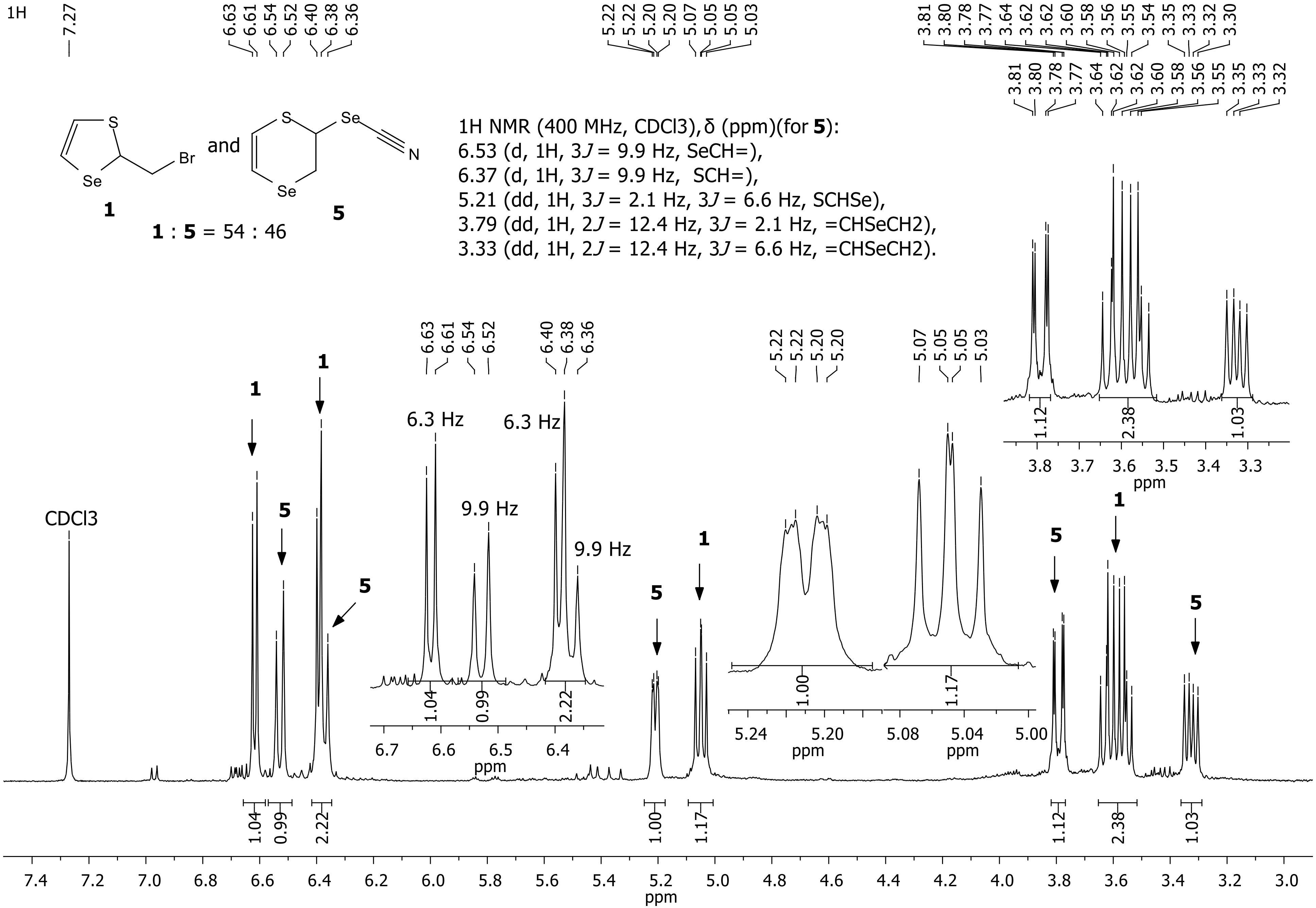

^{1}H NMR spectrum of reaction mixture after 0.5 h (reaction of 1 with KSeCN, 0 °C, acetonitrile)

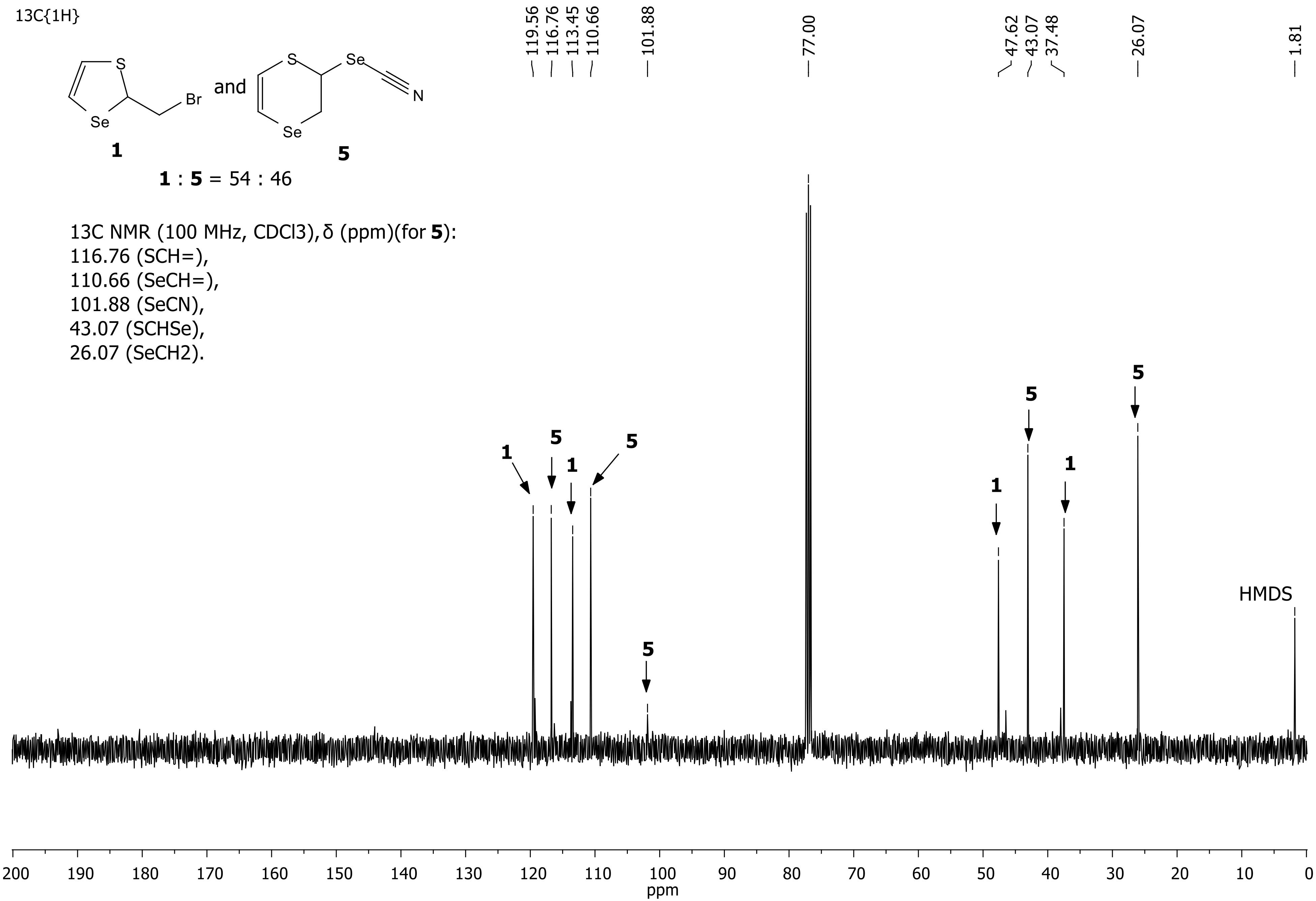

$^{13}C\{^{1}H\}$ NMR spectrum of reaction mixture after 0.5 h (reaction of 1 with KSeCN, 0 ºC, acetonitrile)

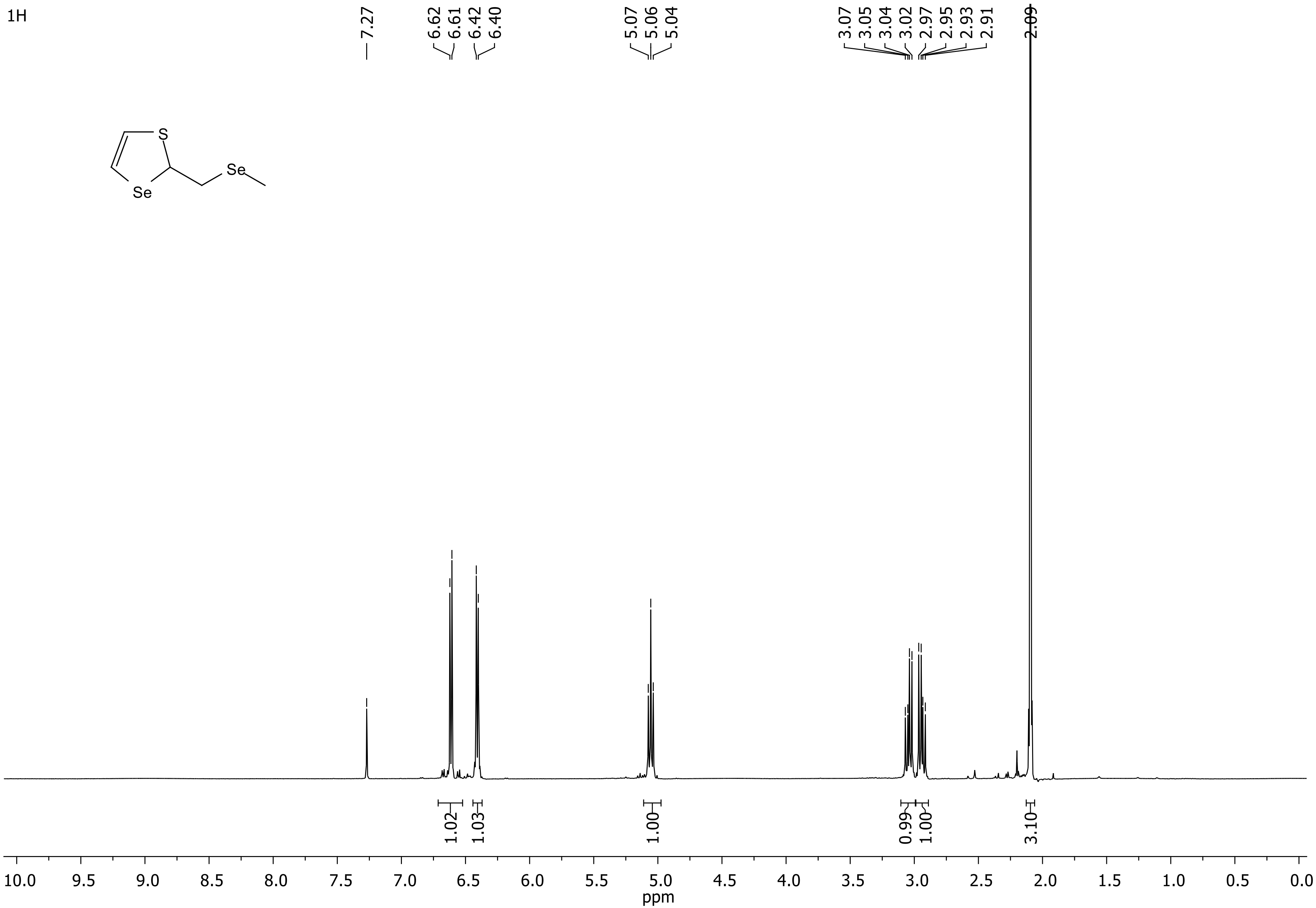

^{1}H NMR spectrum of methyl 1,3-thiaselenol-2-ylmethyl selenide (6a)

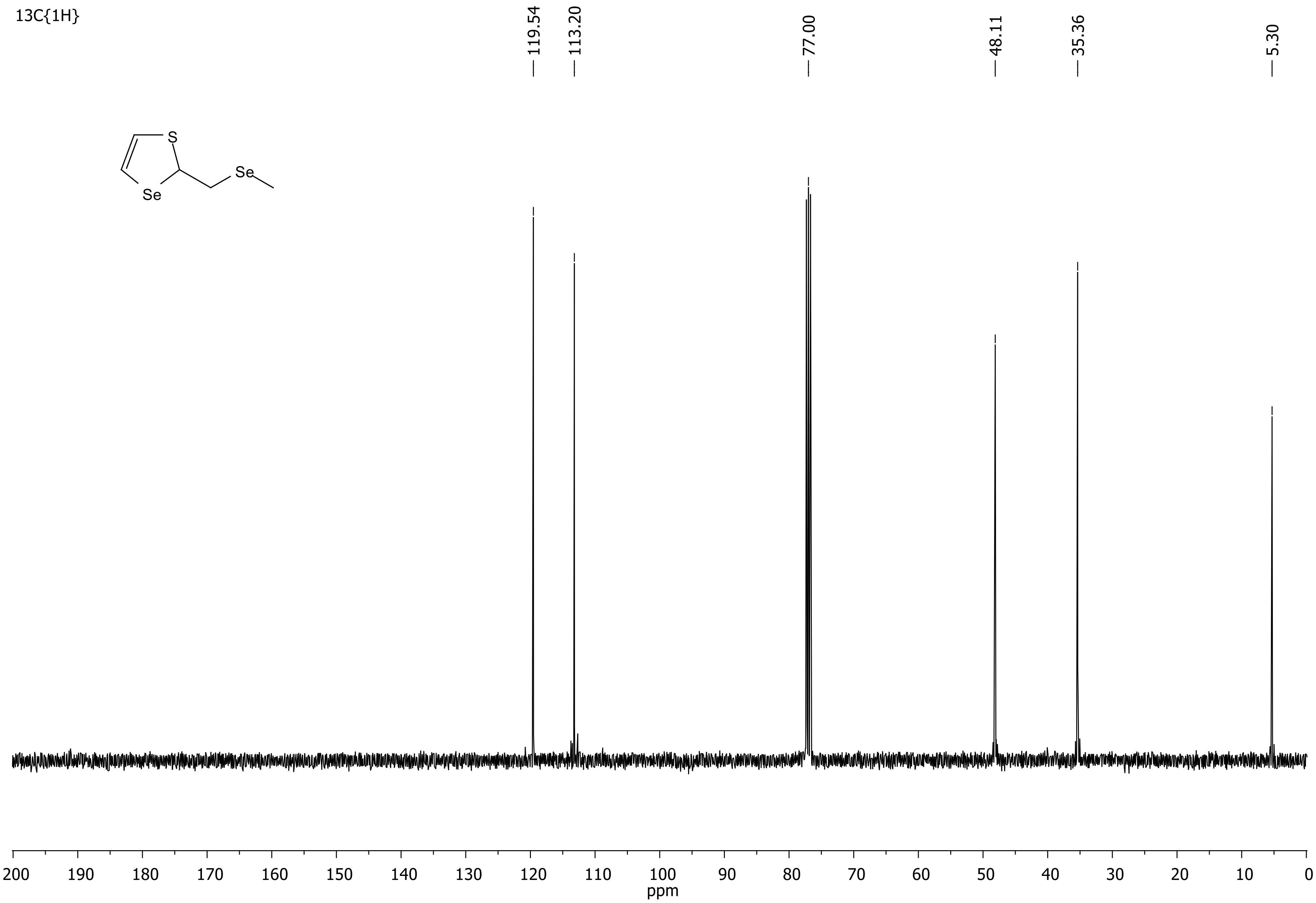

$^{13}C\{^{1}H\}$ NMR spectrum of methyl 1,3-thiaselenol-2-ylmethyl selenide (6a)

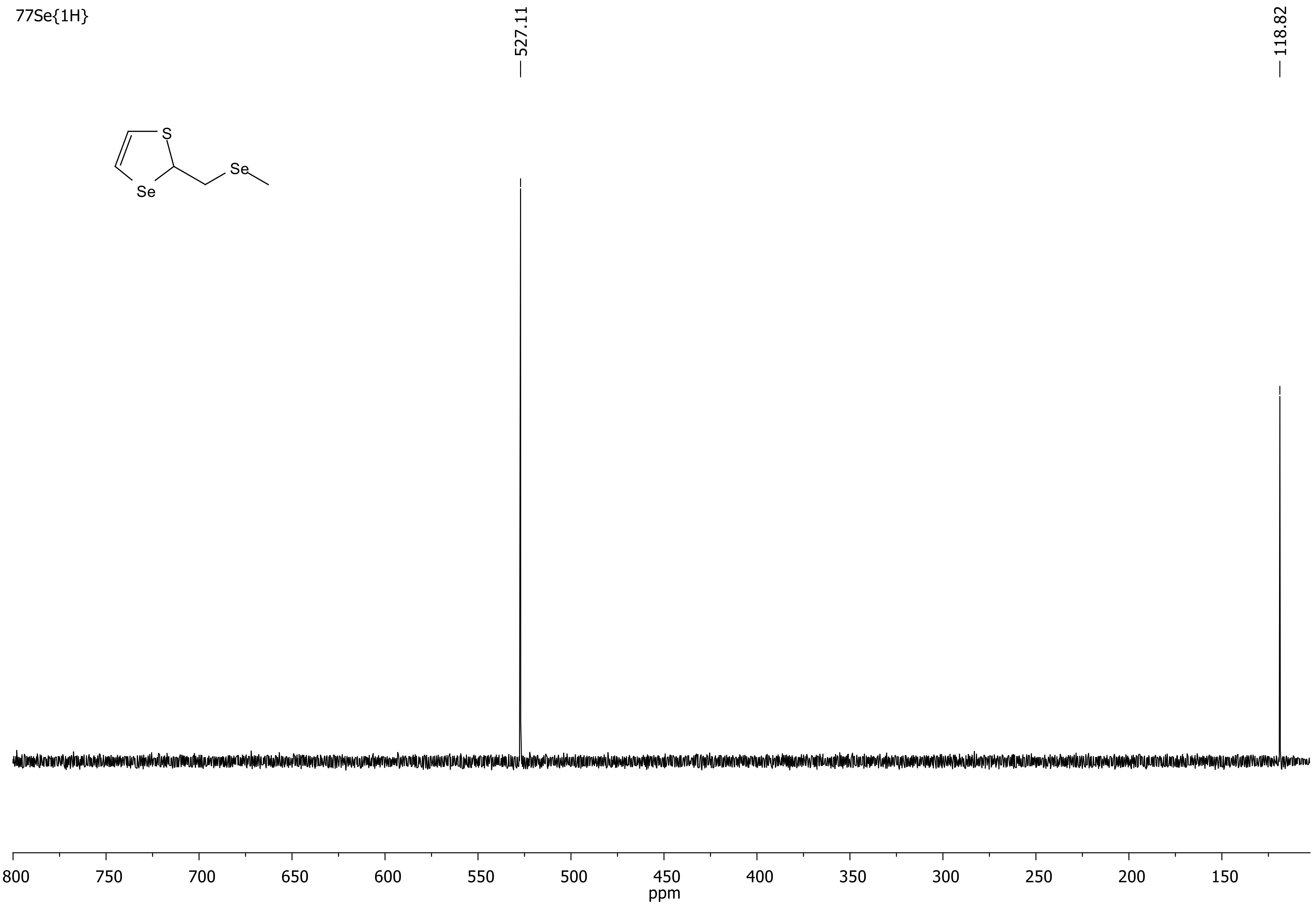

^{77}Se{^{1}H} NMR spectrum of methyl 1,3-thiaselenol-2-ylmethyl selenide (6a)

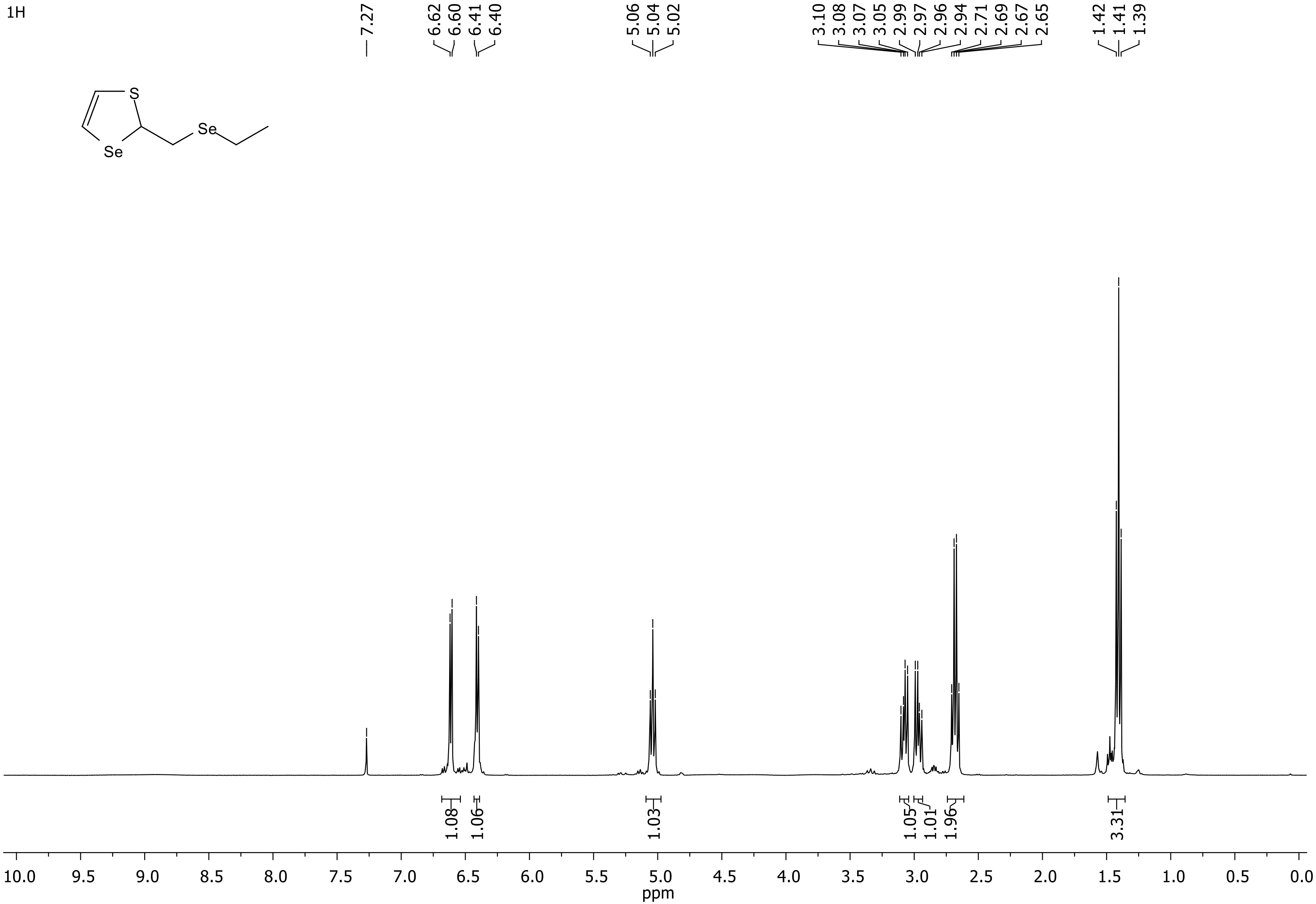

^{1}H NMR spectrum of ethyl 1,3-thiaselenol-2-ylmethyl selenide (6b)

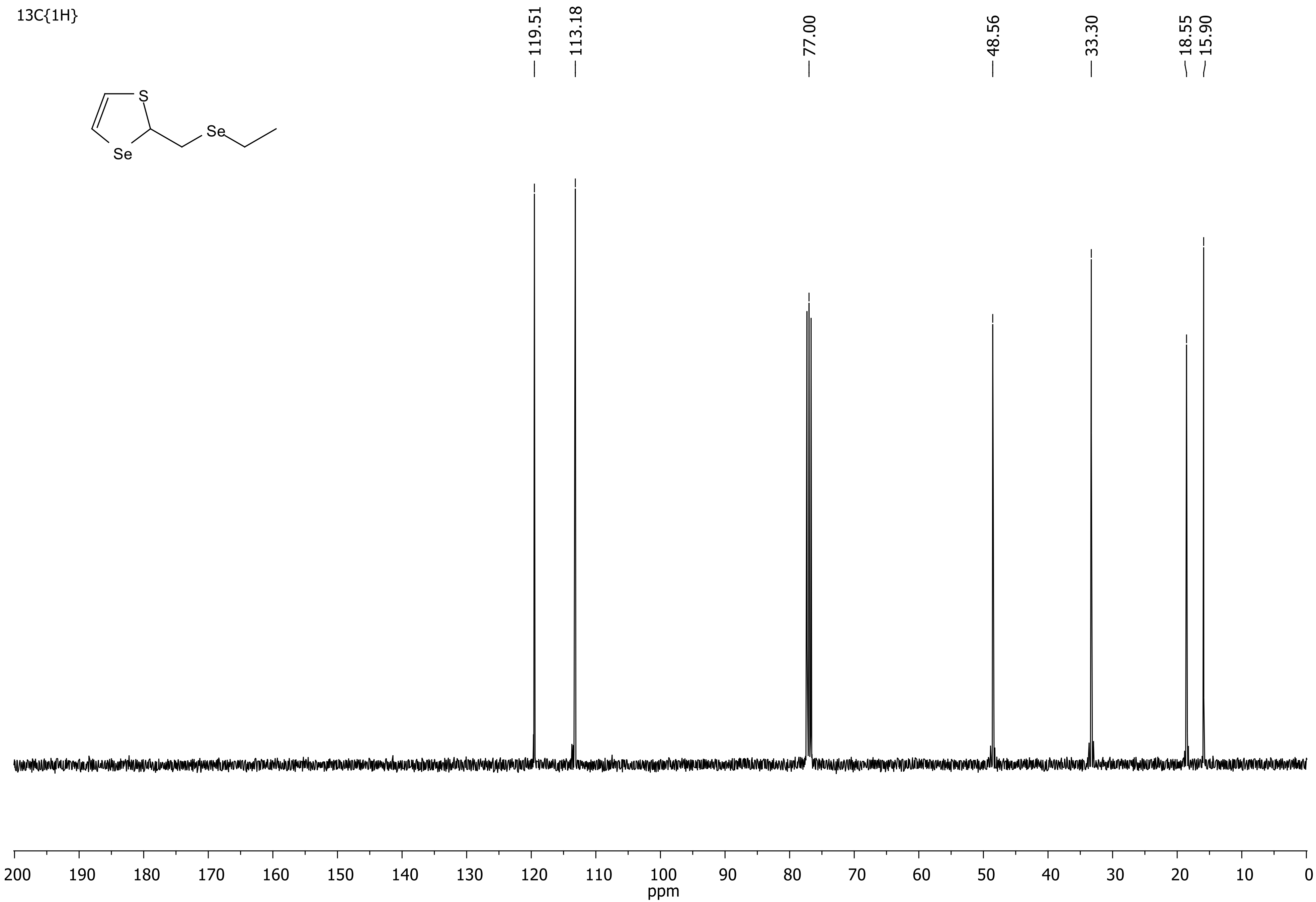

$^{13}C\{^1H\}$ NMR spectrum of ethyl 1,3-thiaselenol-2-ylmethyl selenide (6b)

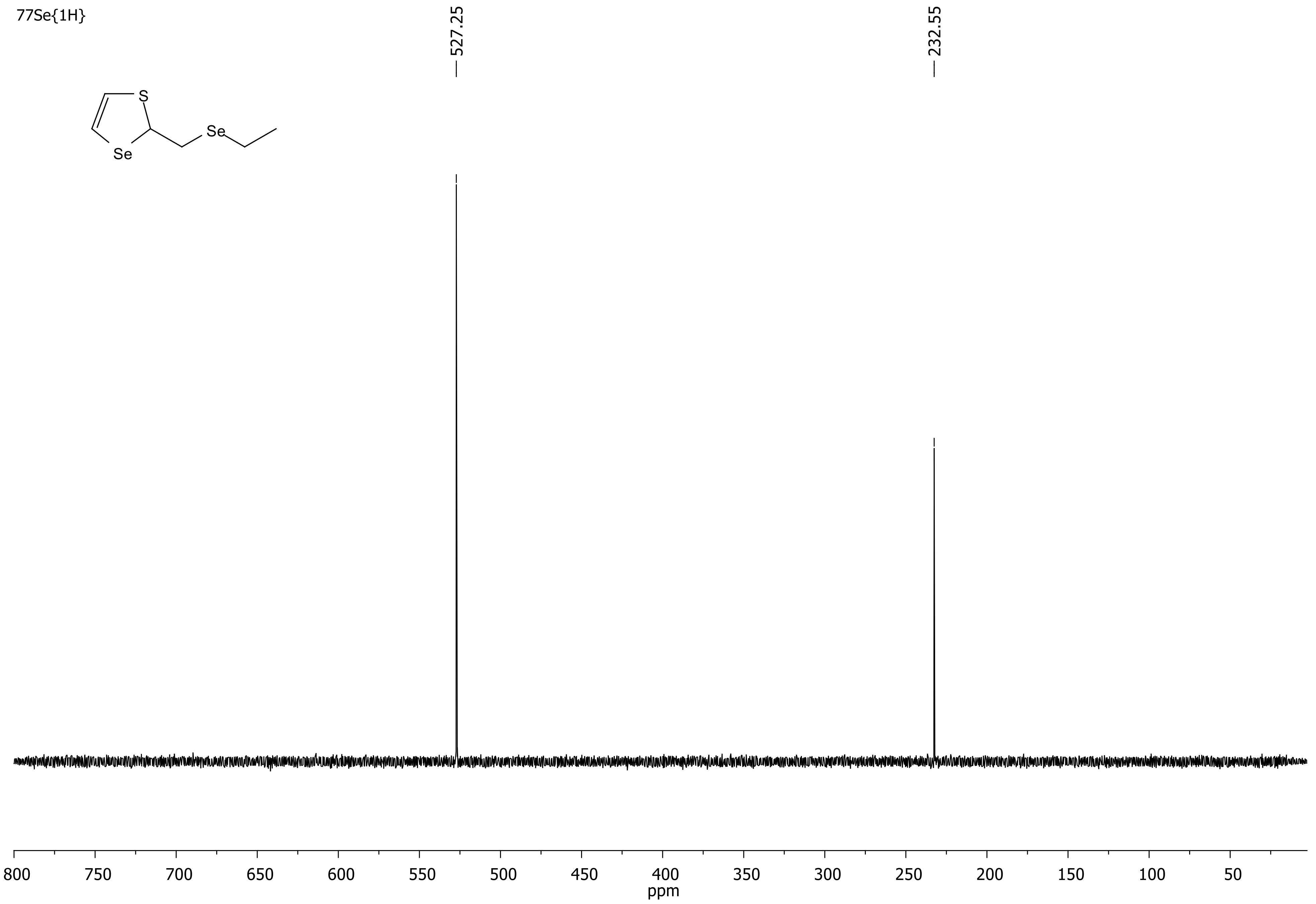

$^{77}Se\{^{1}H\}$ NMR spectrum of ethyl 1,3-thiaselenol-2-ylmethyl selenide (6b)

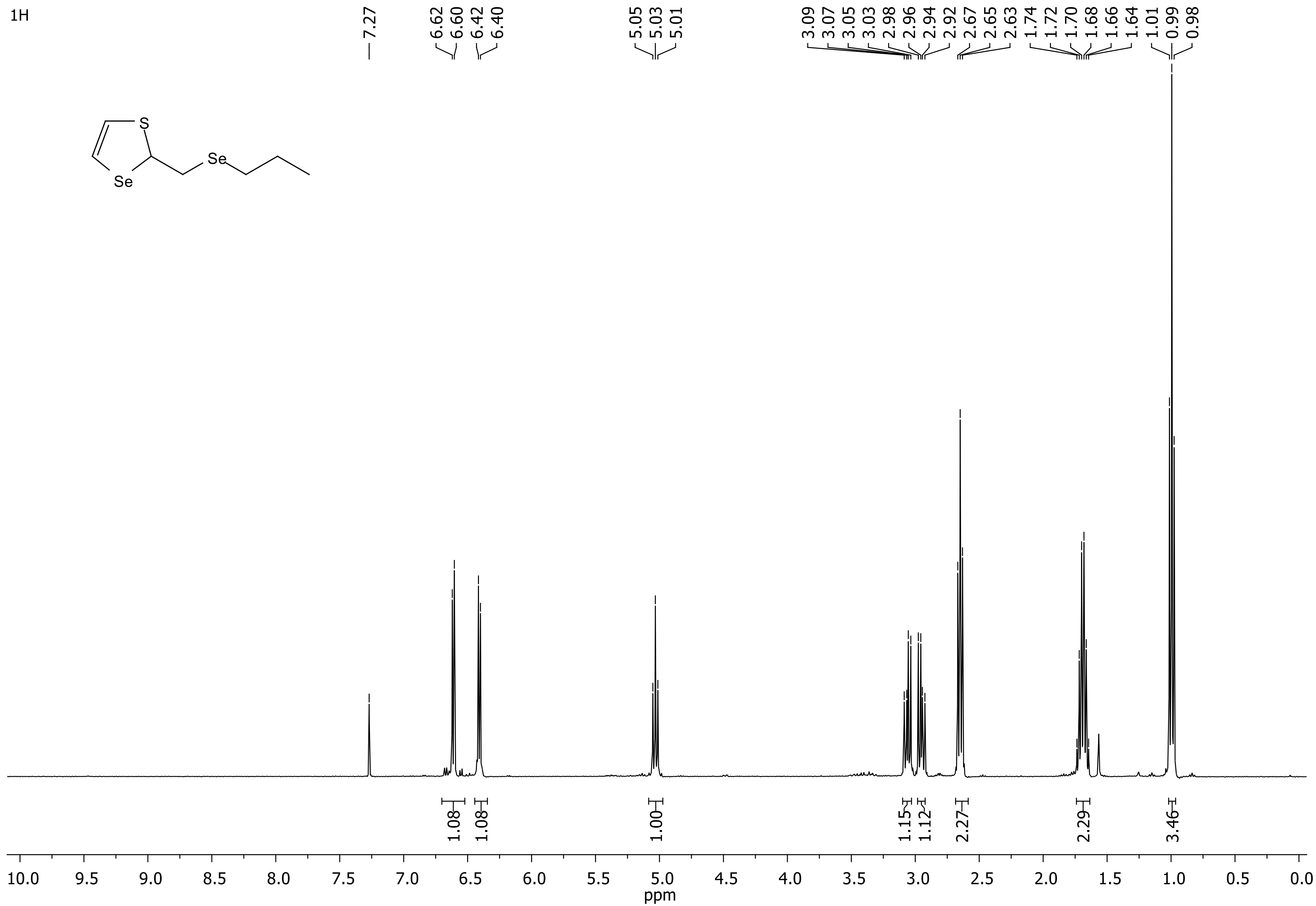

^{1}H NMR spectrum of propyl 1,3-thiaselenol-2-ylmethyl selenide (6c)

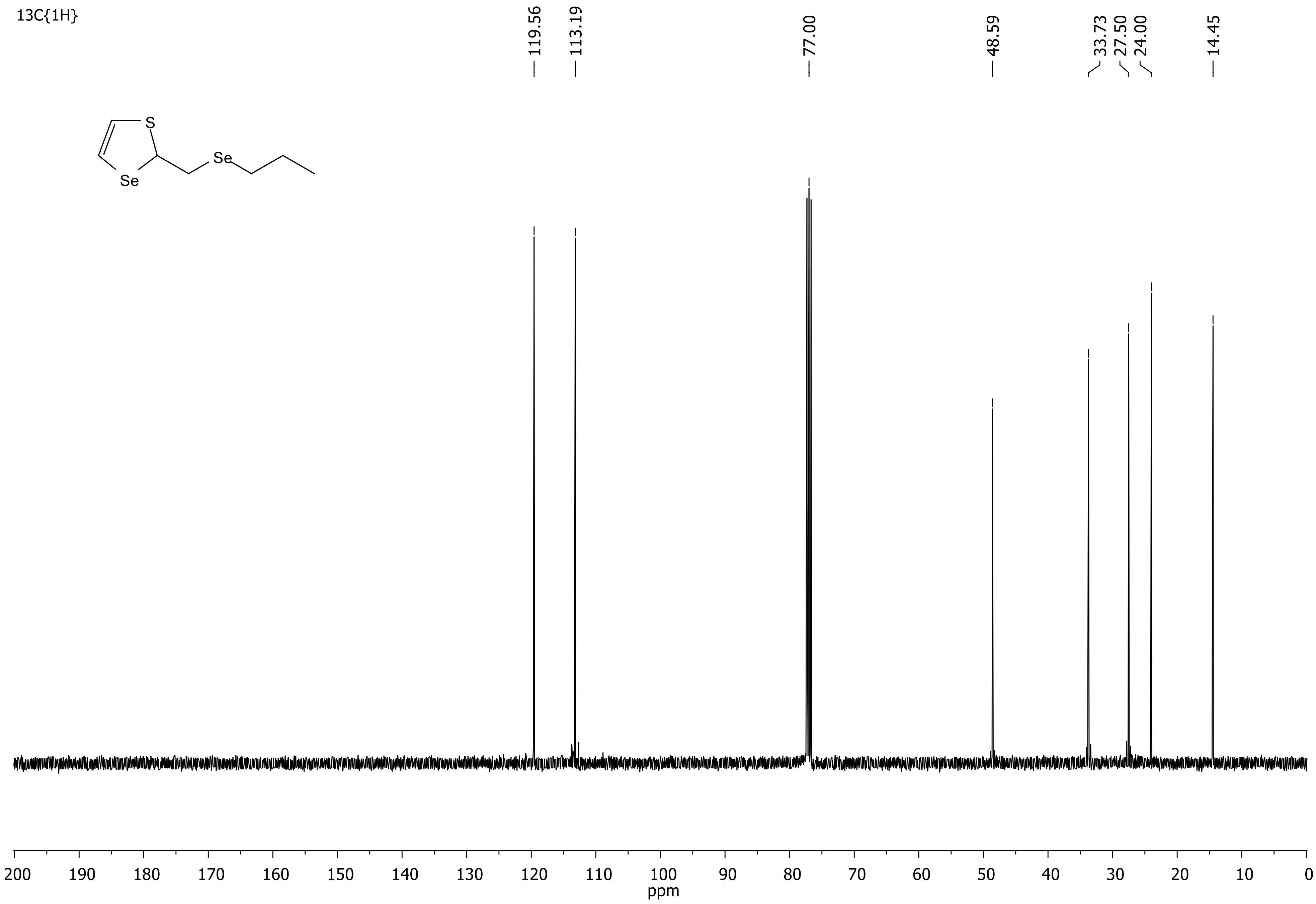

$^{13}C\{^{1}H\}$ NMR spectrum of propyl 1,3-thiaselenol-2-ylmethyl selenide (6c)

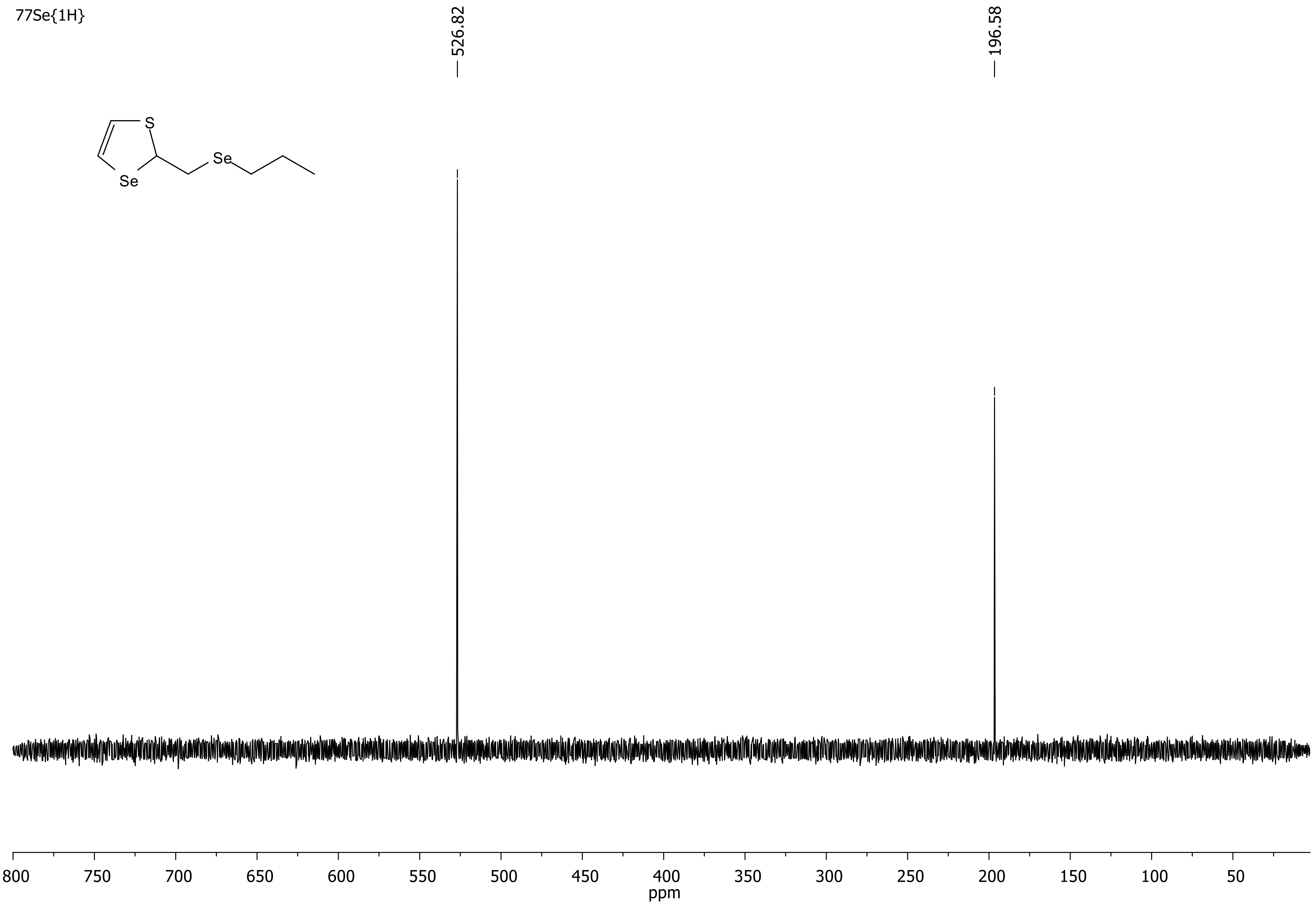

$^{77}Se\{^{1}H\}$ NMR spectrum of propyl 1,3-thiaselenol-2-ylmethyl selenide (6c)

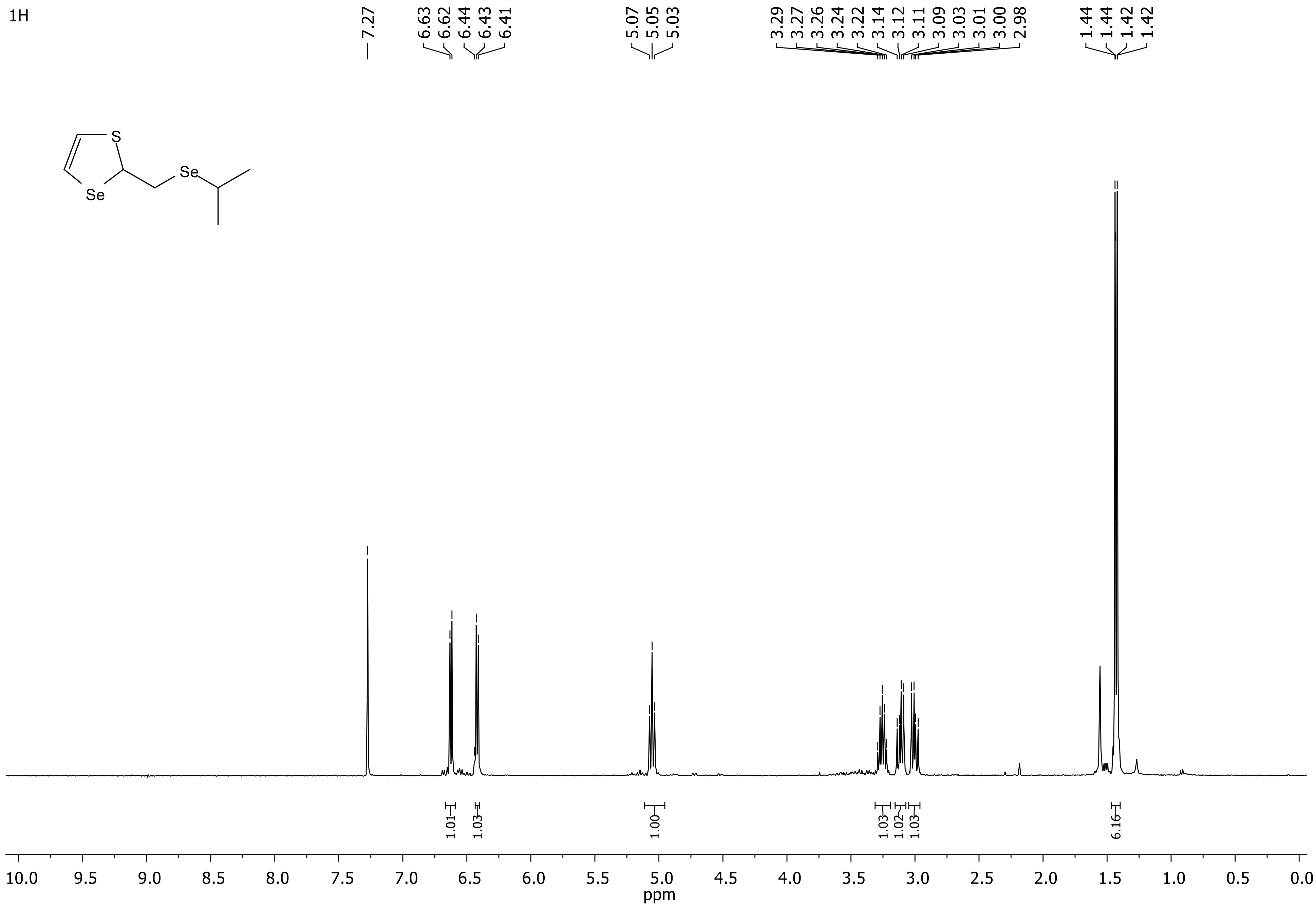

^{1}H NMR spectrum of isopropyl 1,3-thiaselenol-2-ylmethyl selenide (6d)

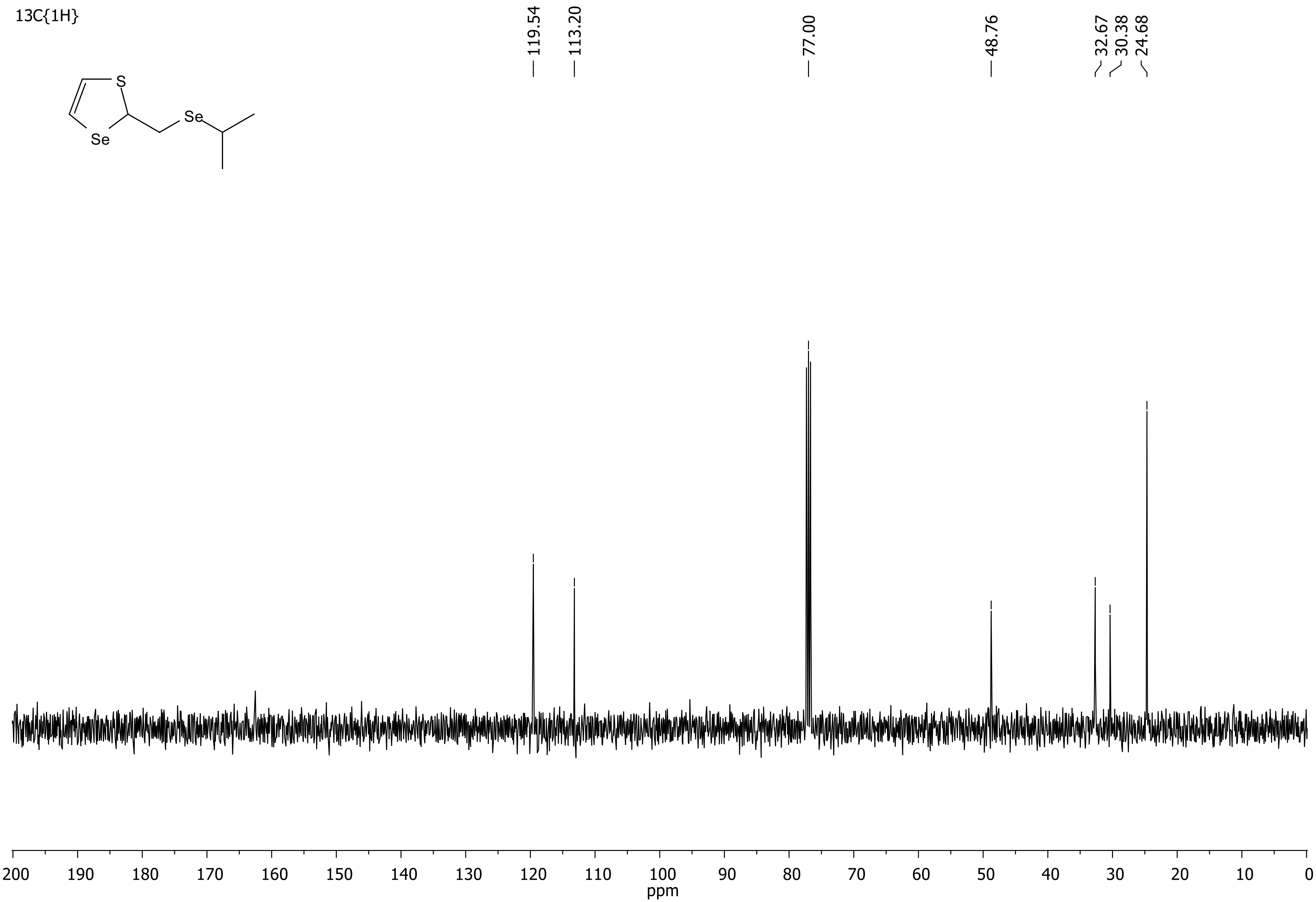

$^{13}C\{^1H\}$ NMR spectrum of isopropyl 1,3-thiaselenol-2-ylmethyl selenide (6d)

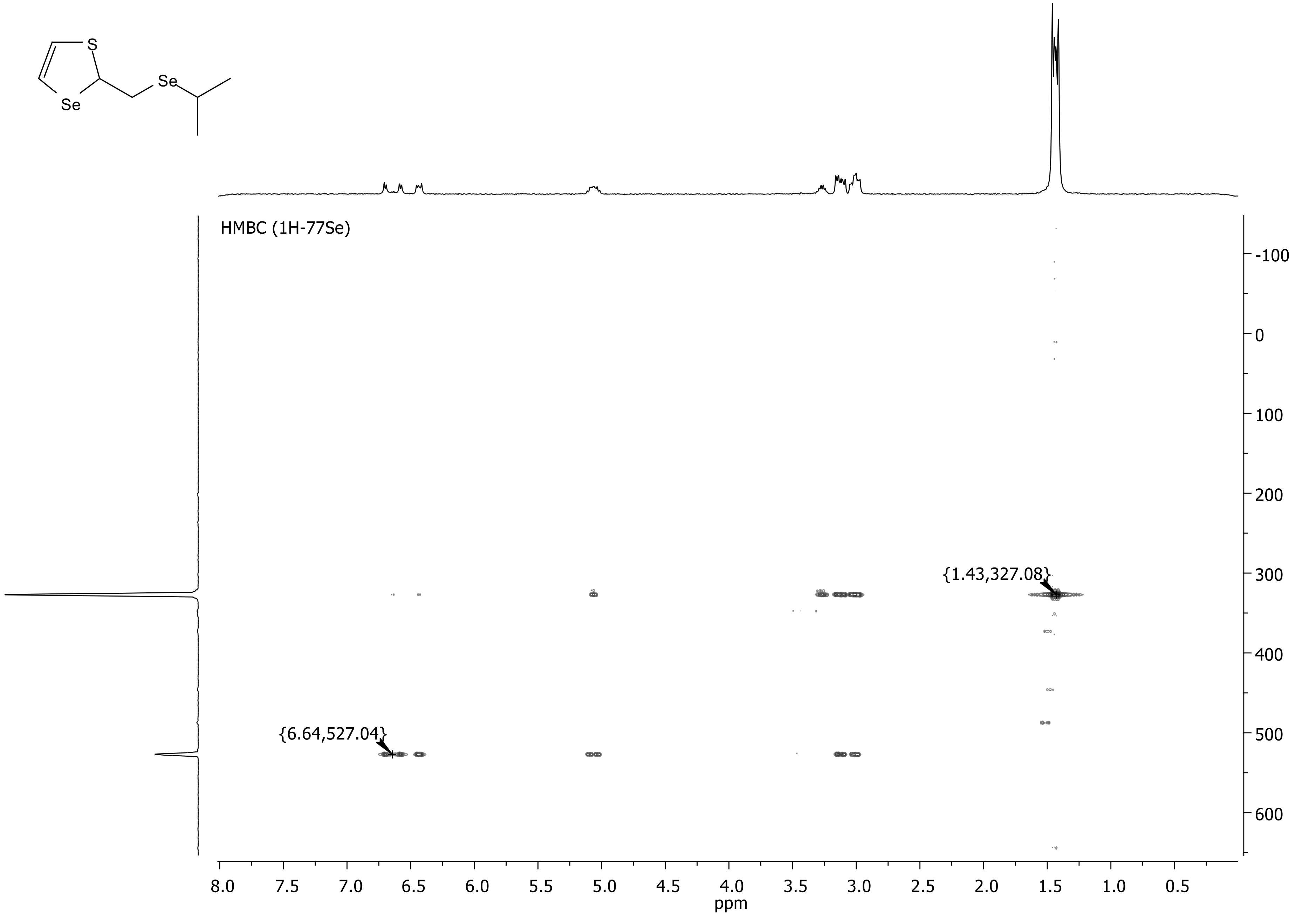

HMBC (^{1}H-^{77}Se) NMR spectrum of isopropyl 1,3-thiaselenol-2-ylmethyl selenide (6d)

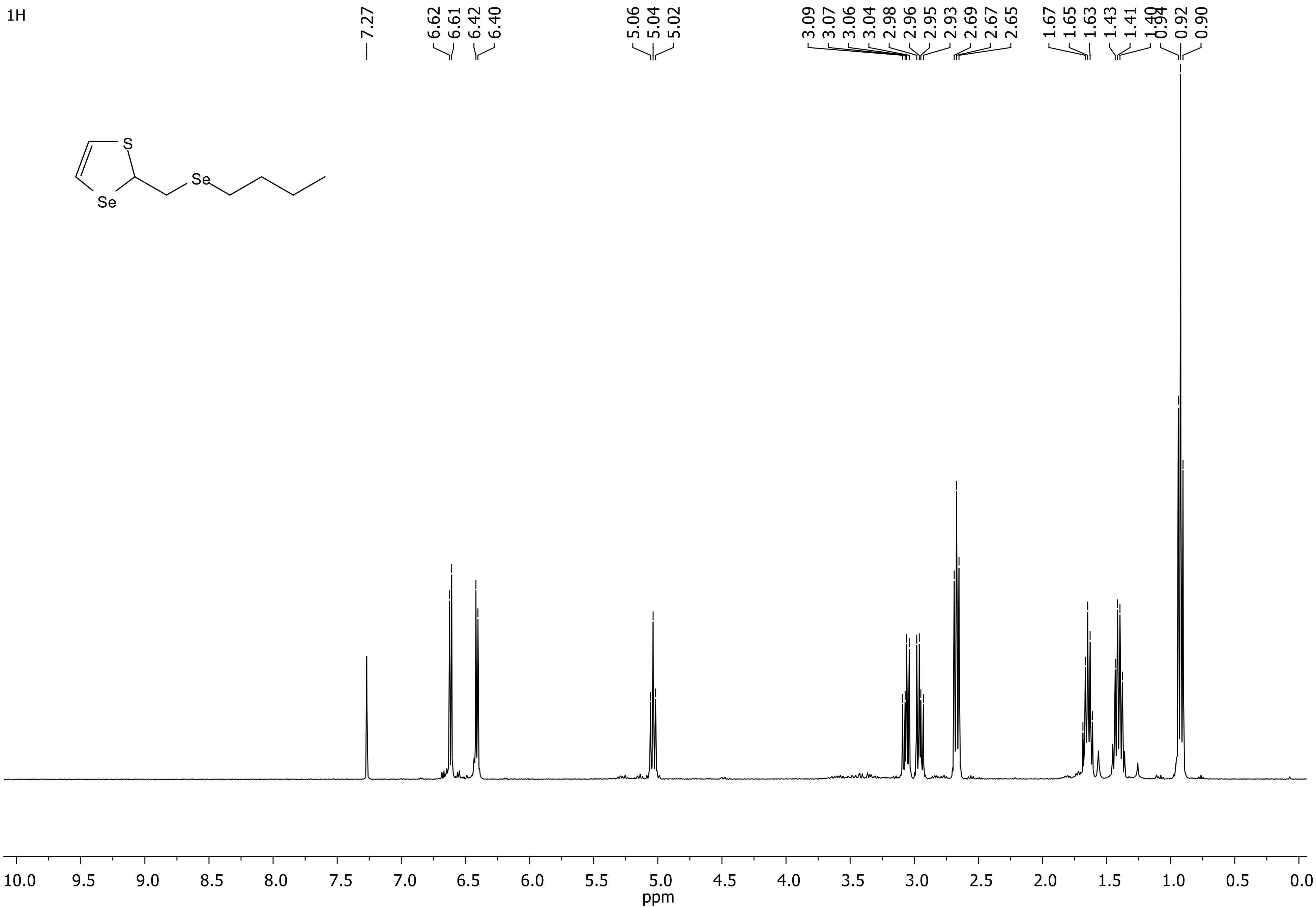

^{1}H NMR spectrum of butyl 1,3-thiaselenol-2-ylmethyl selenide (6e)

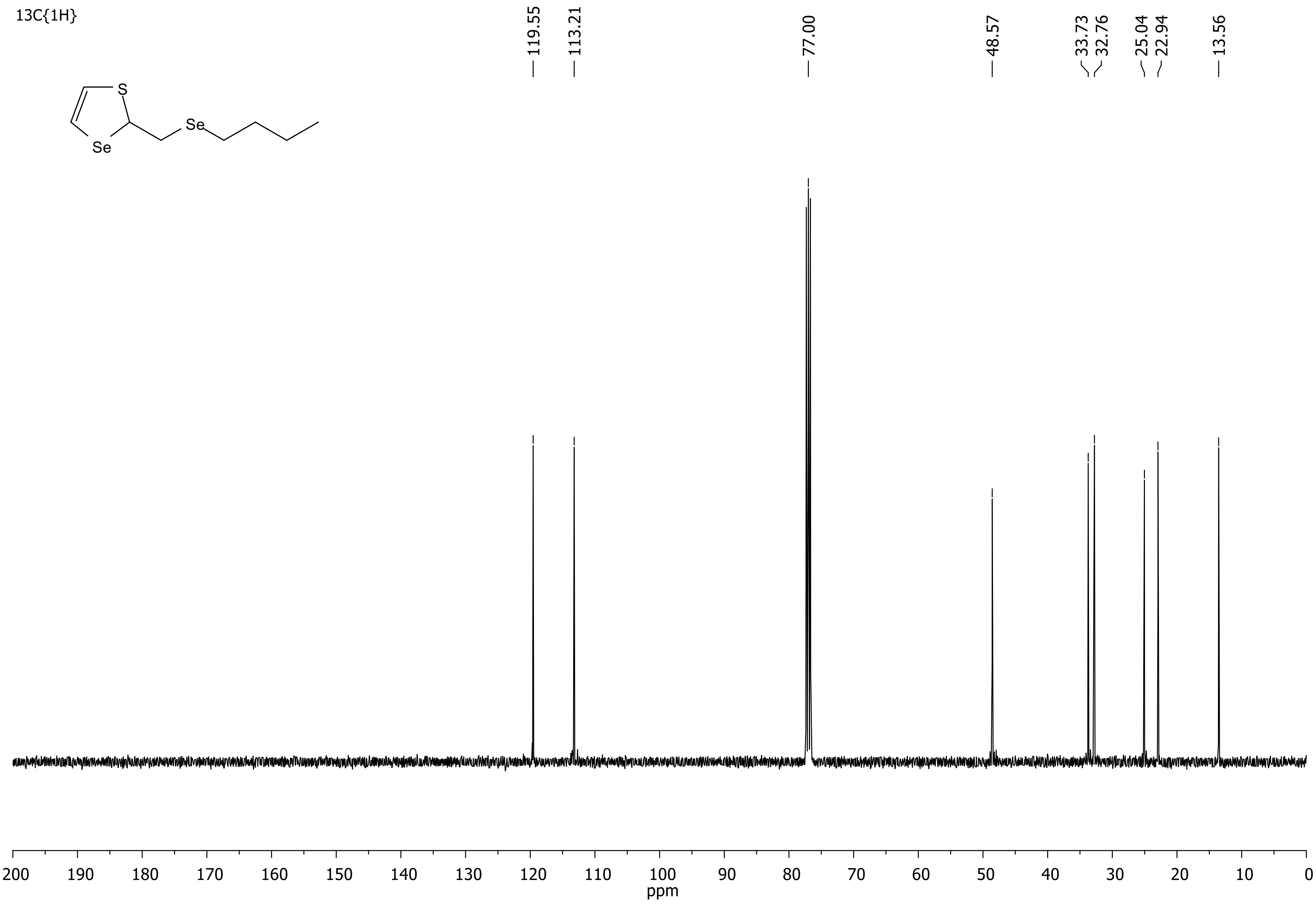

$^{13}C\{^1H\}$ NMR spectrum of butyl 1,3-thiaselenol-2-ylmethyl selenide (6e)

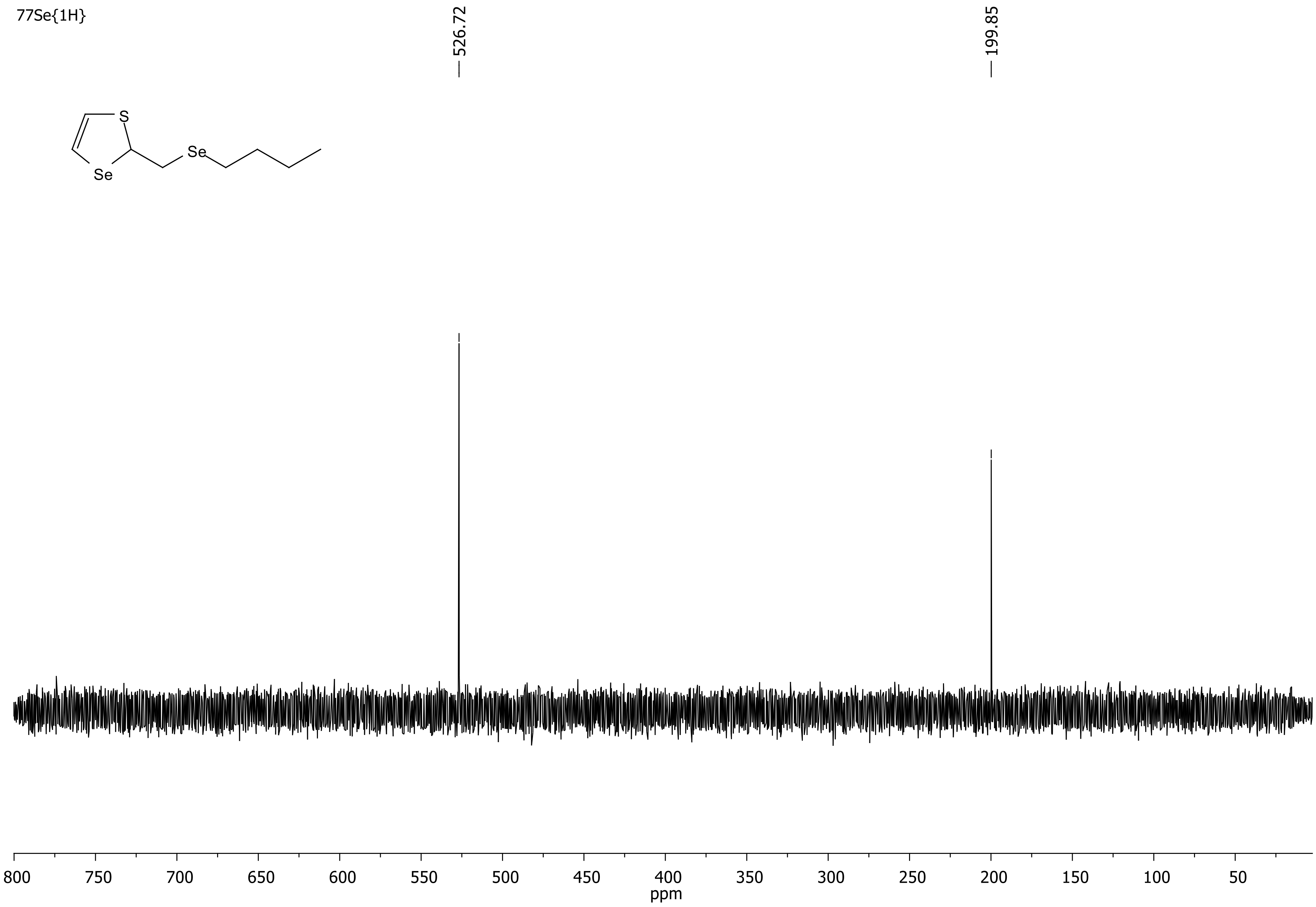

^{77}Se{^{1}H} NMR spectrum of butyl 1,3-thiaselenol-2-ylmethyl selenide (6e)

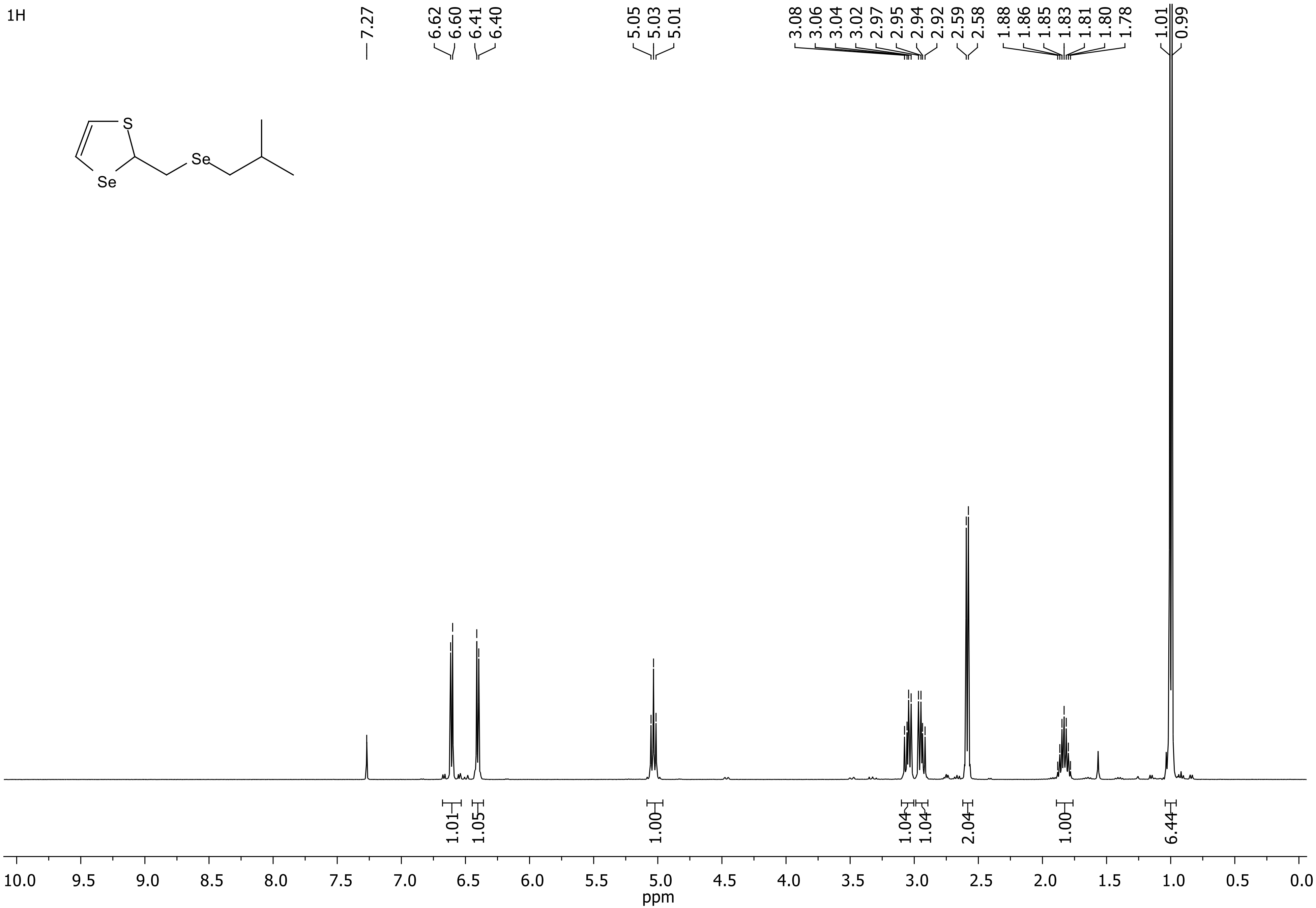

^{1}H NMR spectrum of isobutyl 1,3-thiaselenol-2-ylmethyl selenide (6f)

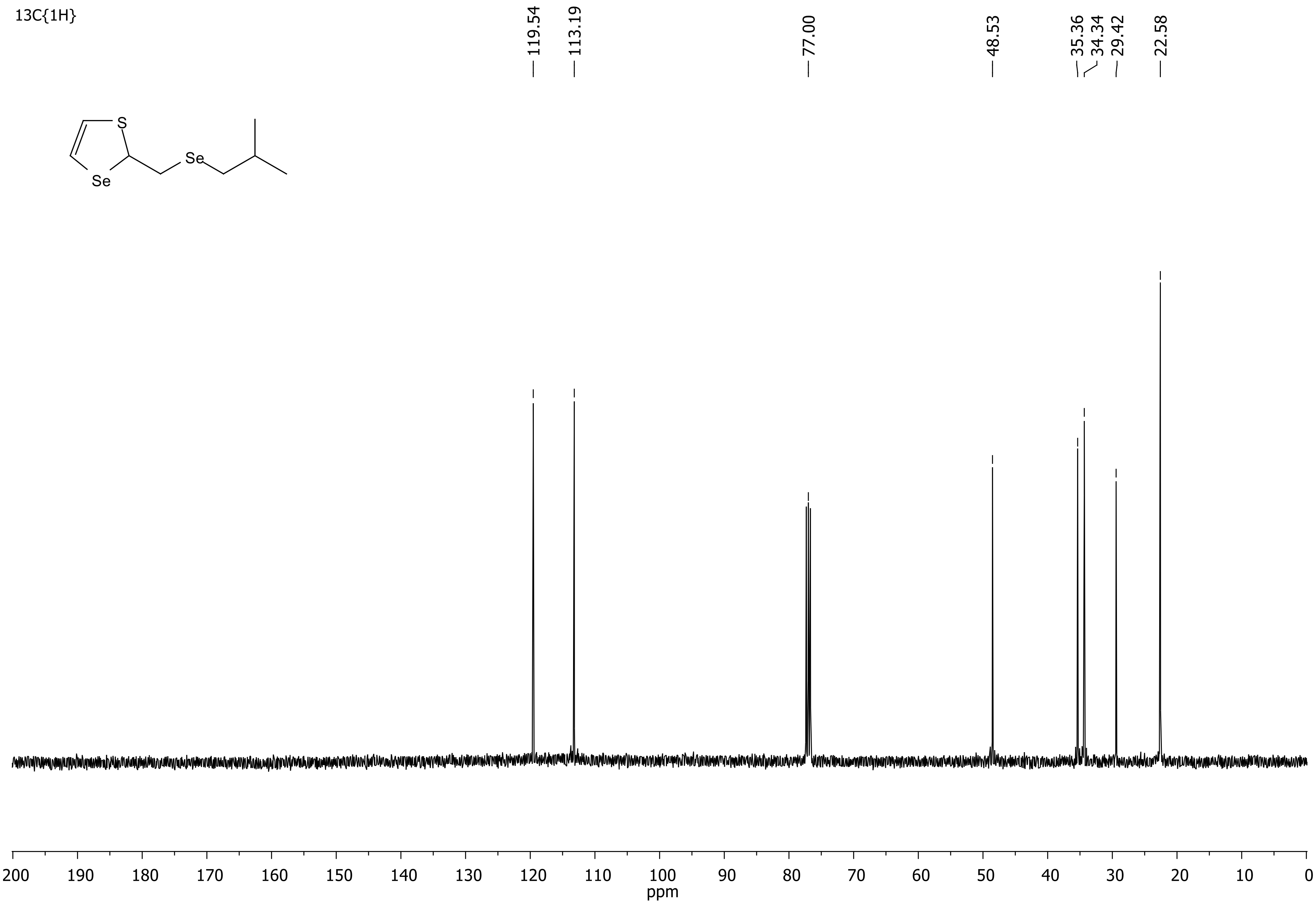

$^{13}C\{^{1}H\}$ NMR spectrum of isobutyl 1,3-thiaselenol-2-ylmethyl selenide (6f)

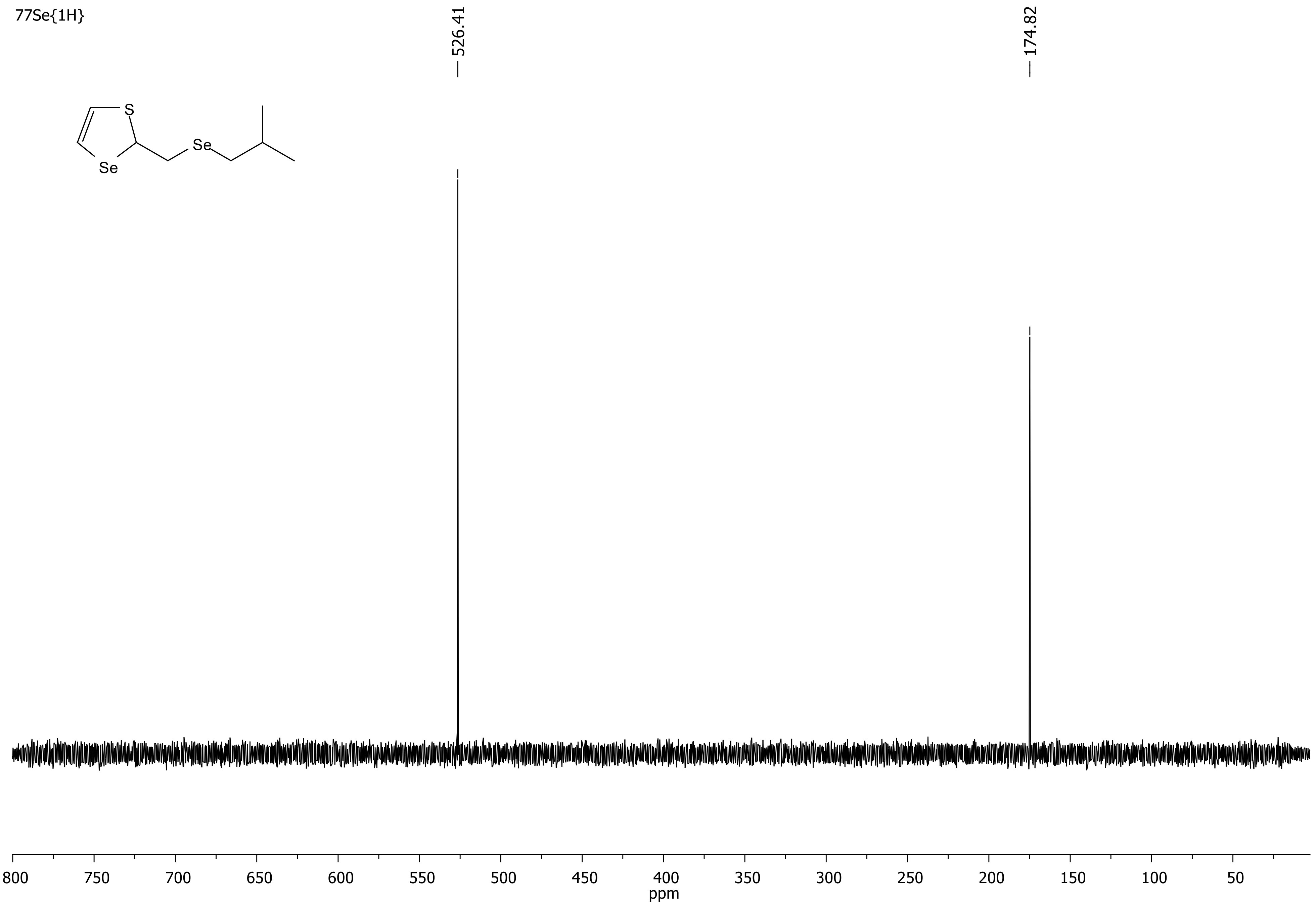

^{77}Se{^{1}H} NMR spectrum of isobutyl 1,3-thiaselenol-2-ylmethyl selenide (6f)

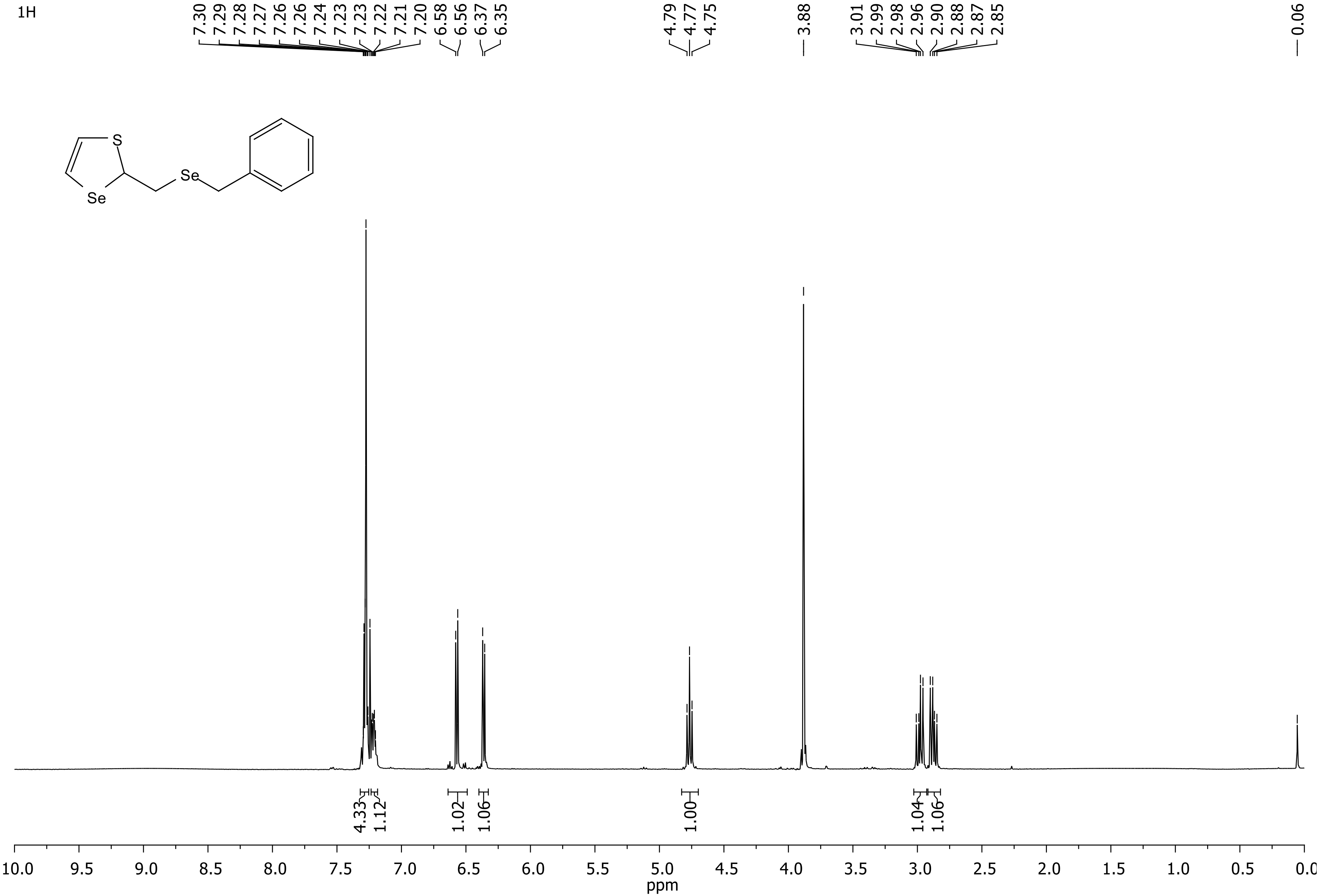

^{1}H NMR spectrum of benzyl 1,3-thiaselenol-2-ylmethyl selenide (6g)

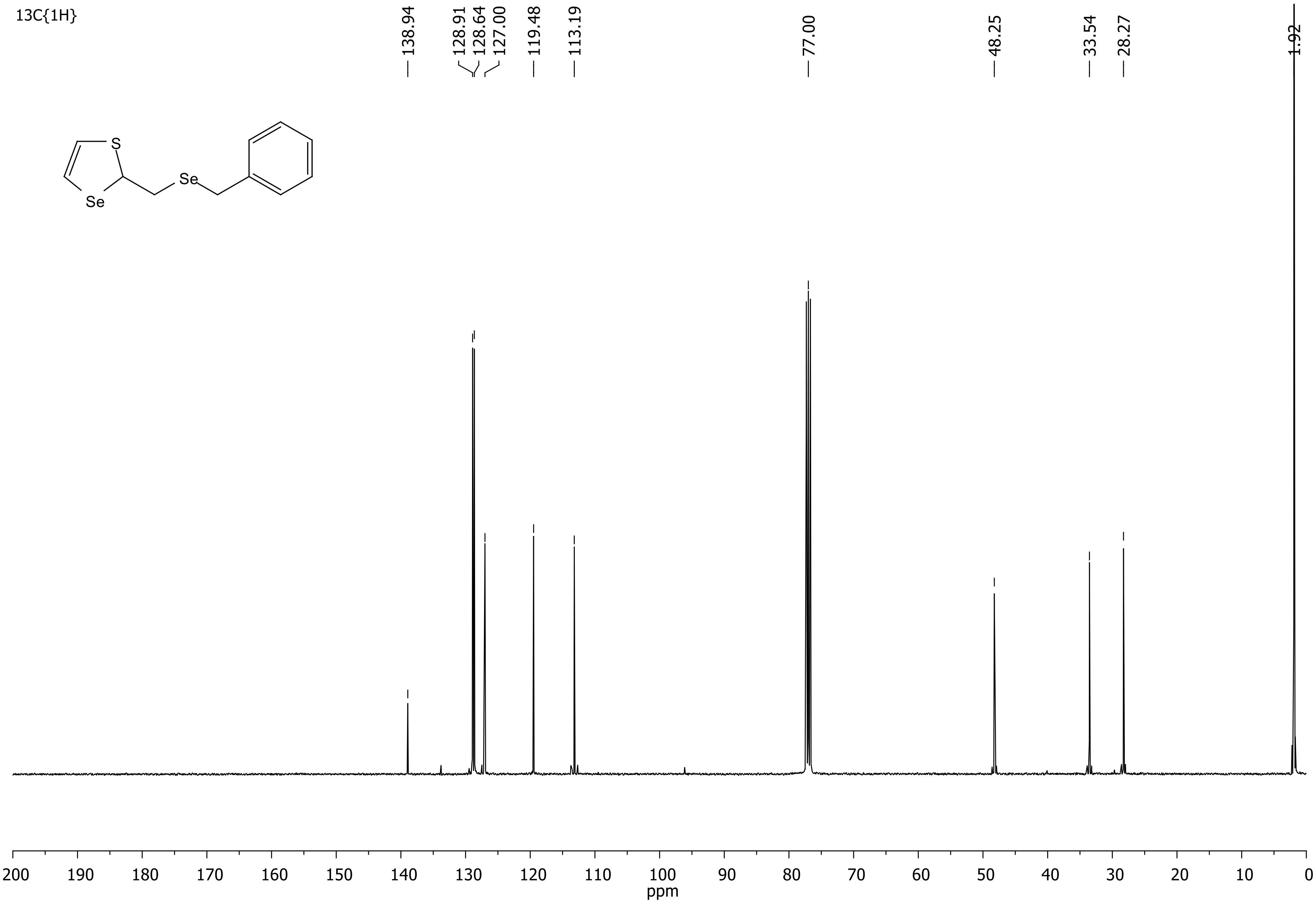

$^{13}C\{^1H\}$ NMR spectrum of benzyl 1,3-thiaselenol-2-ylmethyl selenide (6g)

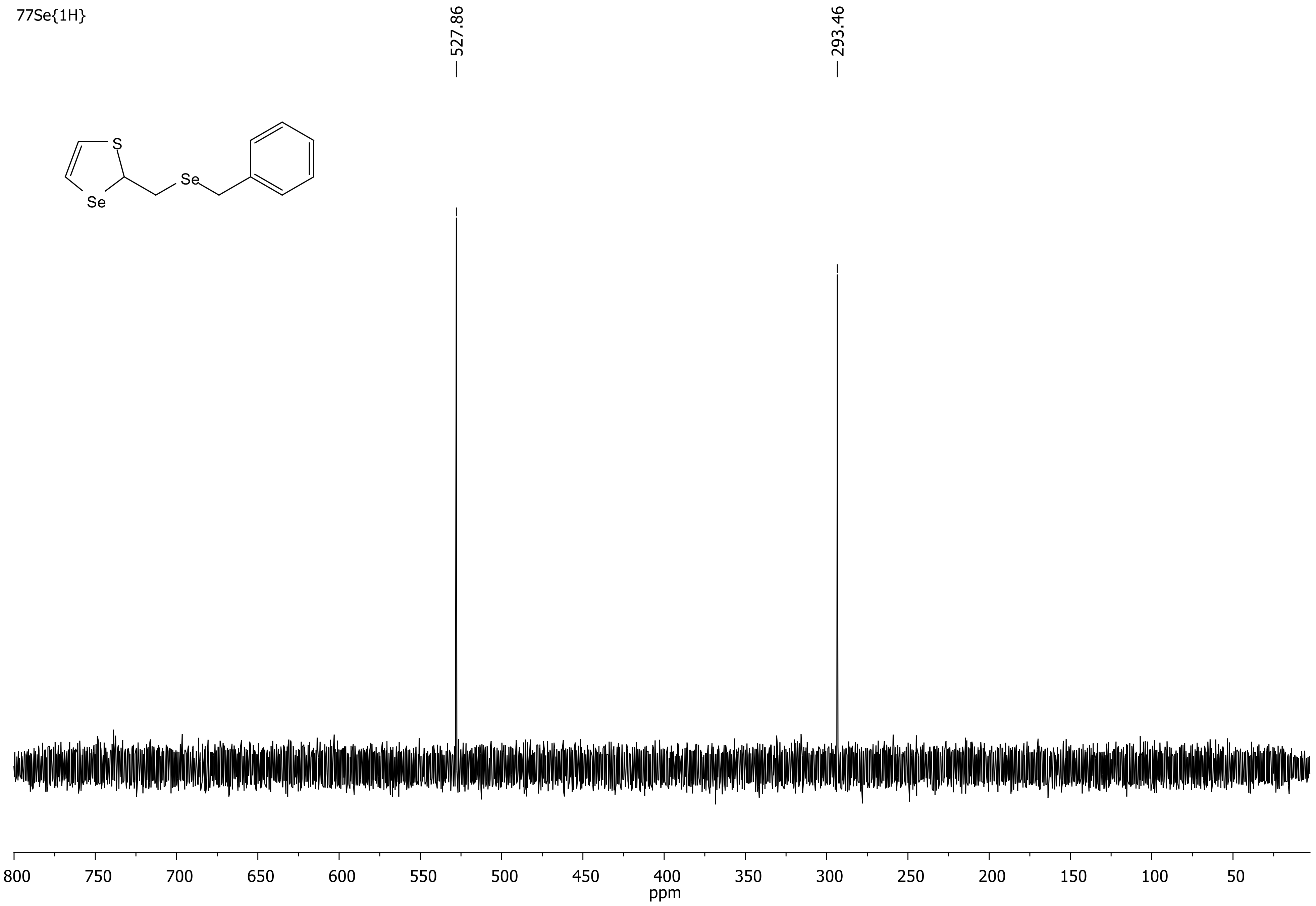

^{77}Se{^{1}H} NMR spectrum of benzyl 1,3-thiaselenol-2-ylmethyl selenide (6g)

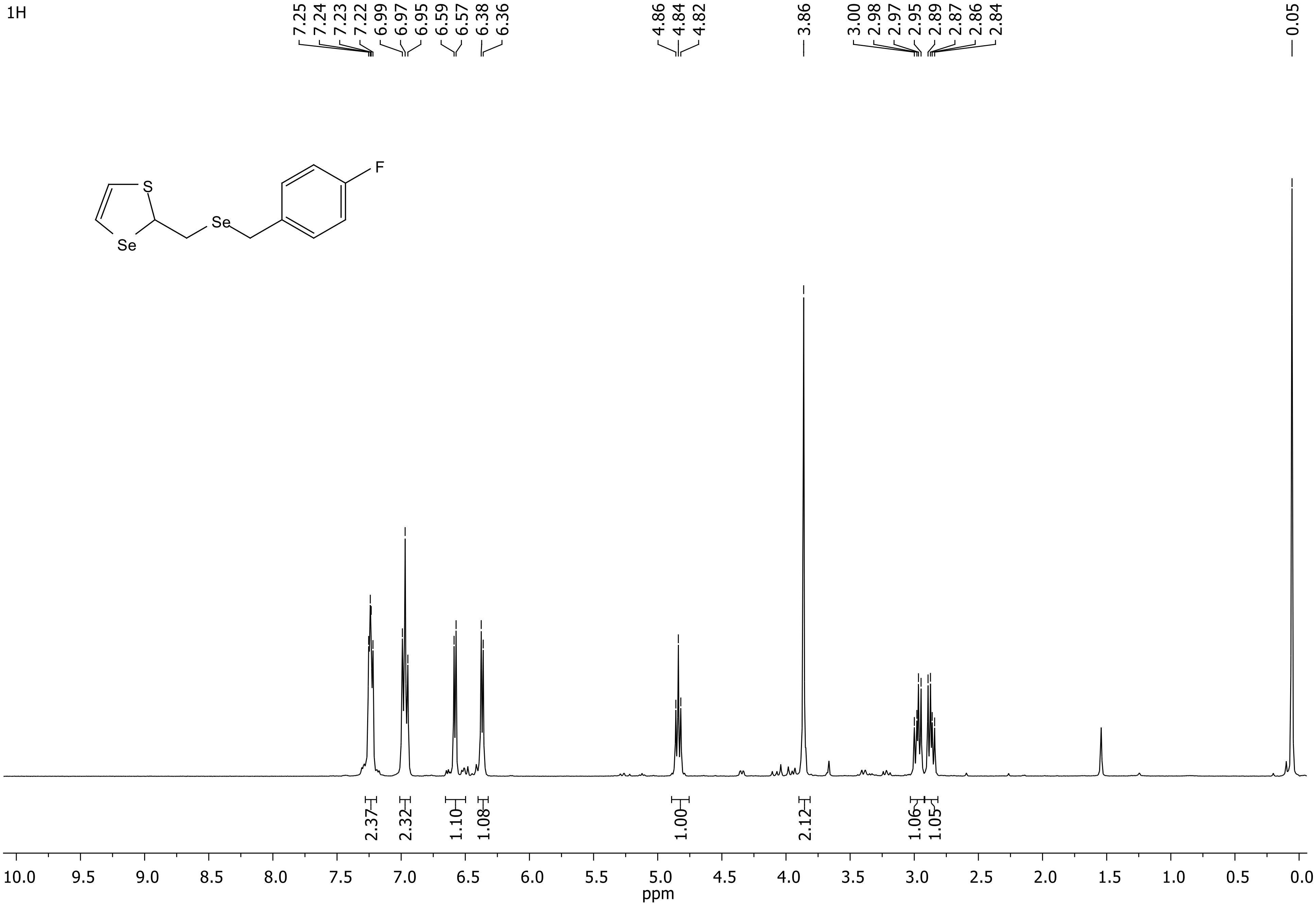

^{1}H NMR spectrum of 4-fluorobenzyl 1,3-thiaselenol-2-ylmethyl selenide (6h)

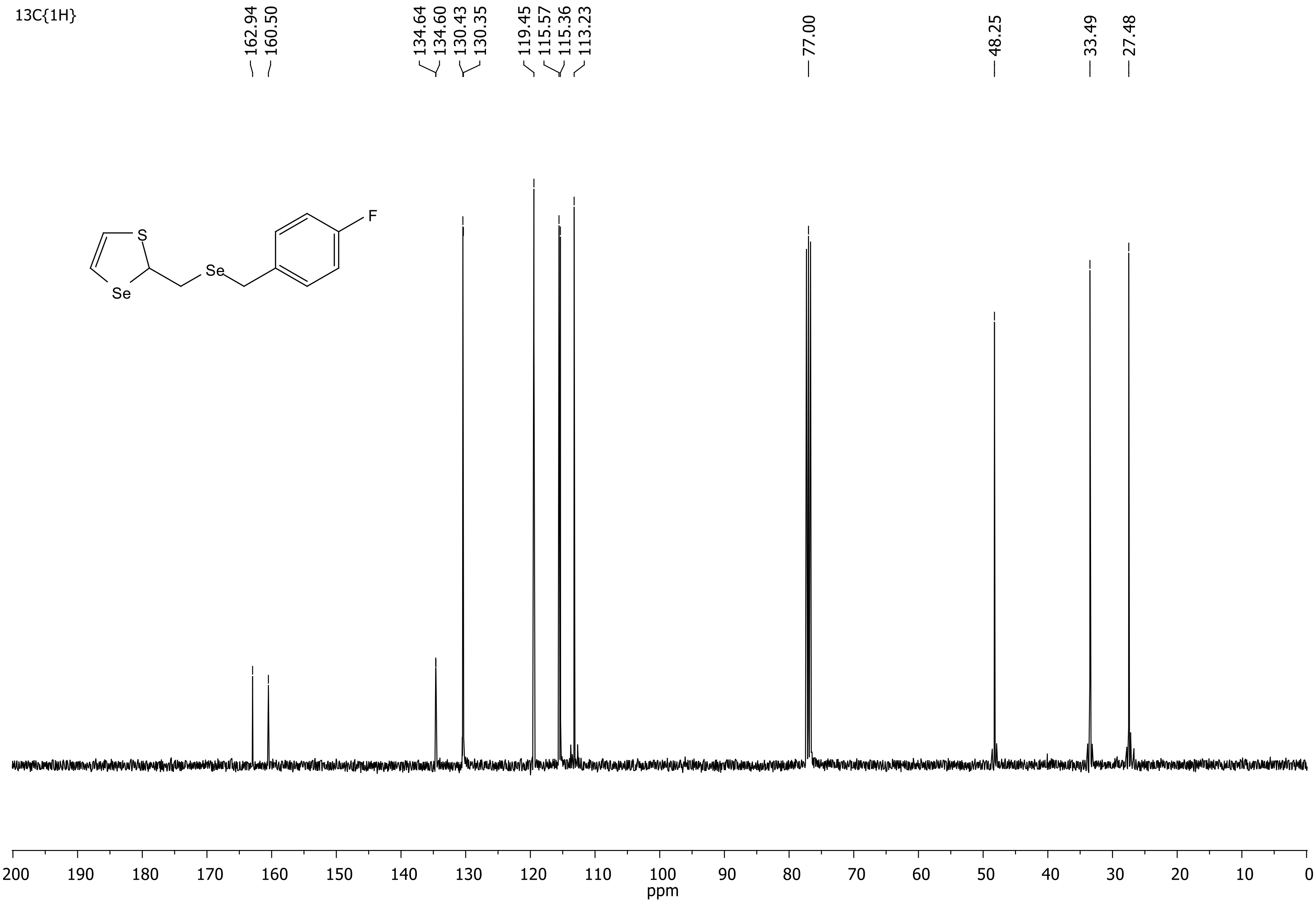

$^{13}C\{^1H\}$ NMR spectrum of 4-fluorobenzyl 1,3-thiaselenol-2-ylmethyl selenide (6h)

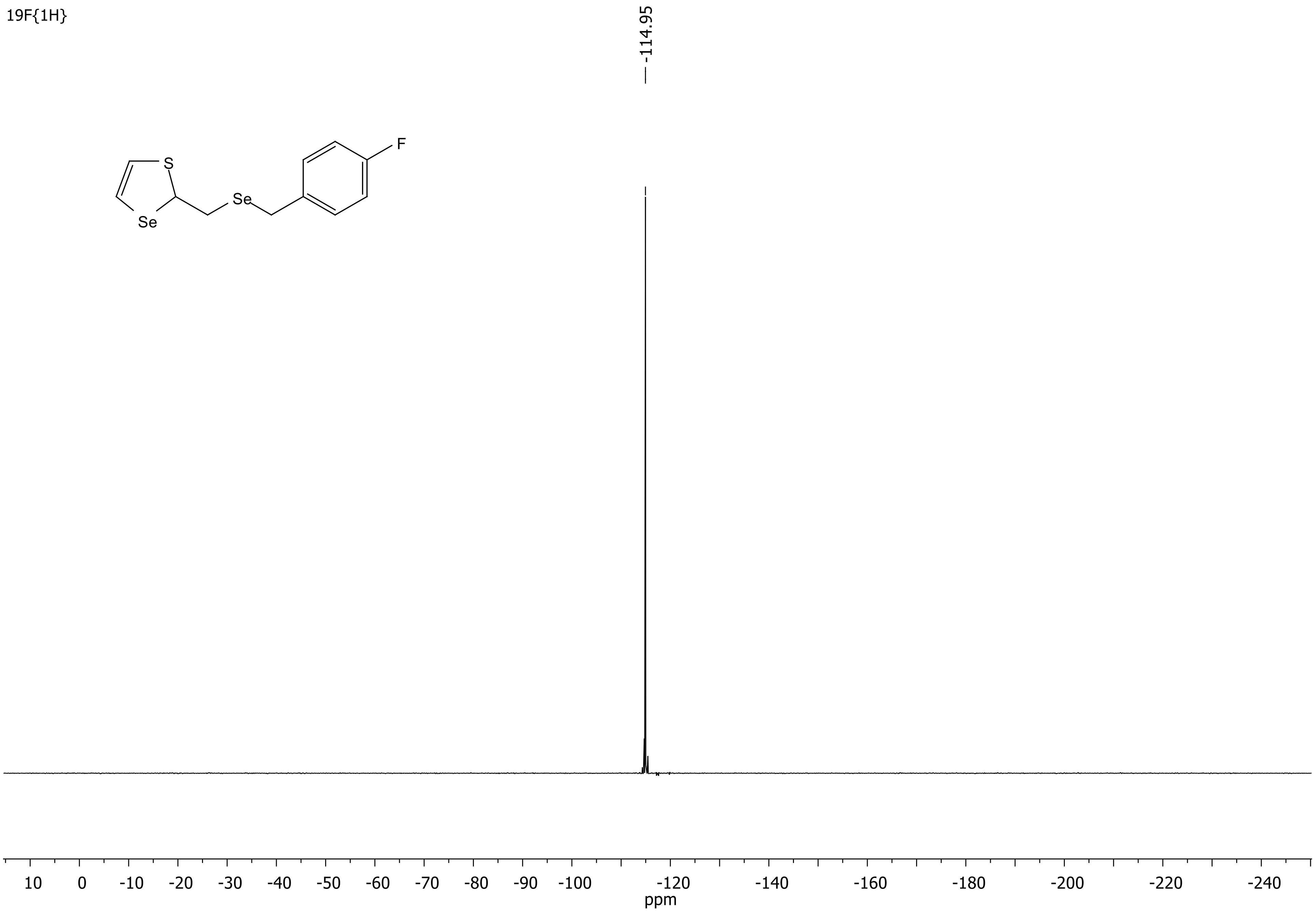

$^{19}F\{^1H\}$ NMR spectrum of 4-fluorobenzyl 1,3-thiaselenol-2-ylmethyl selenide (6h)

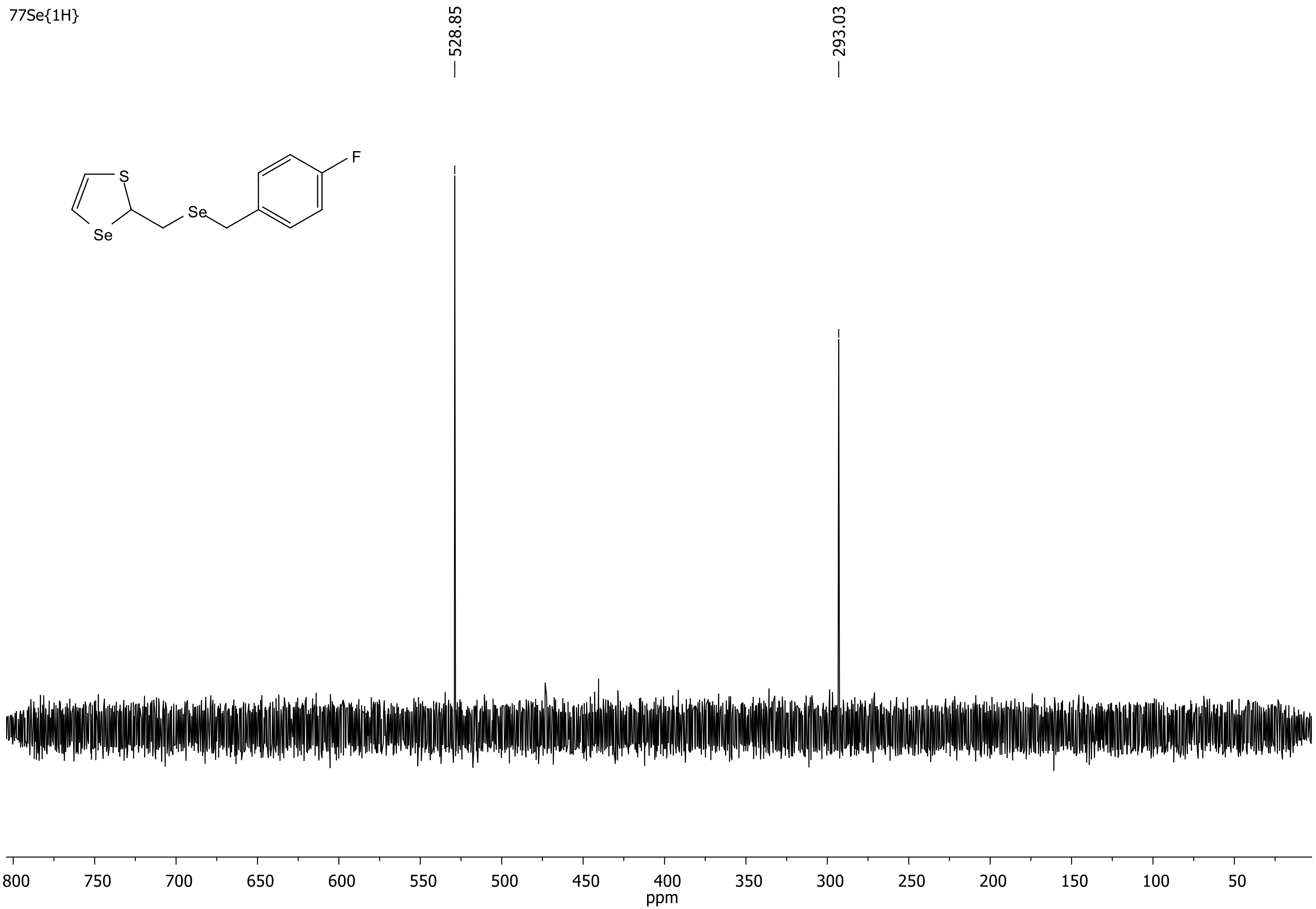

$^{77}Se\{^{1}H\}$ NMR spectrum of 4-fluorobenzyl 1,3-thiaselenol-2-ylmethyl selenide (6h)

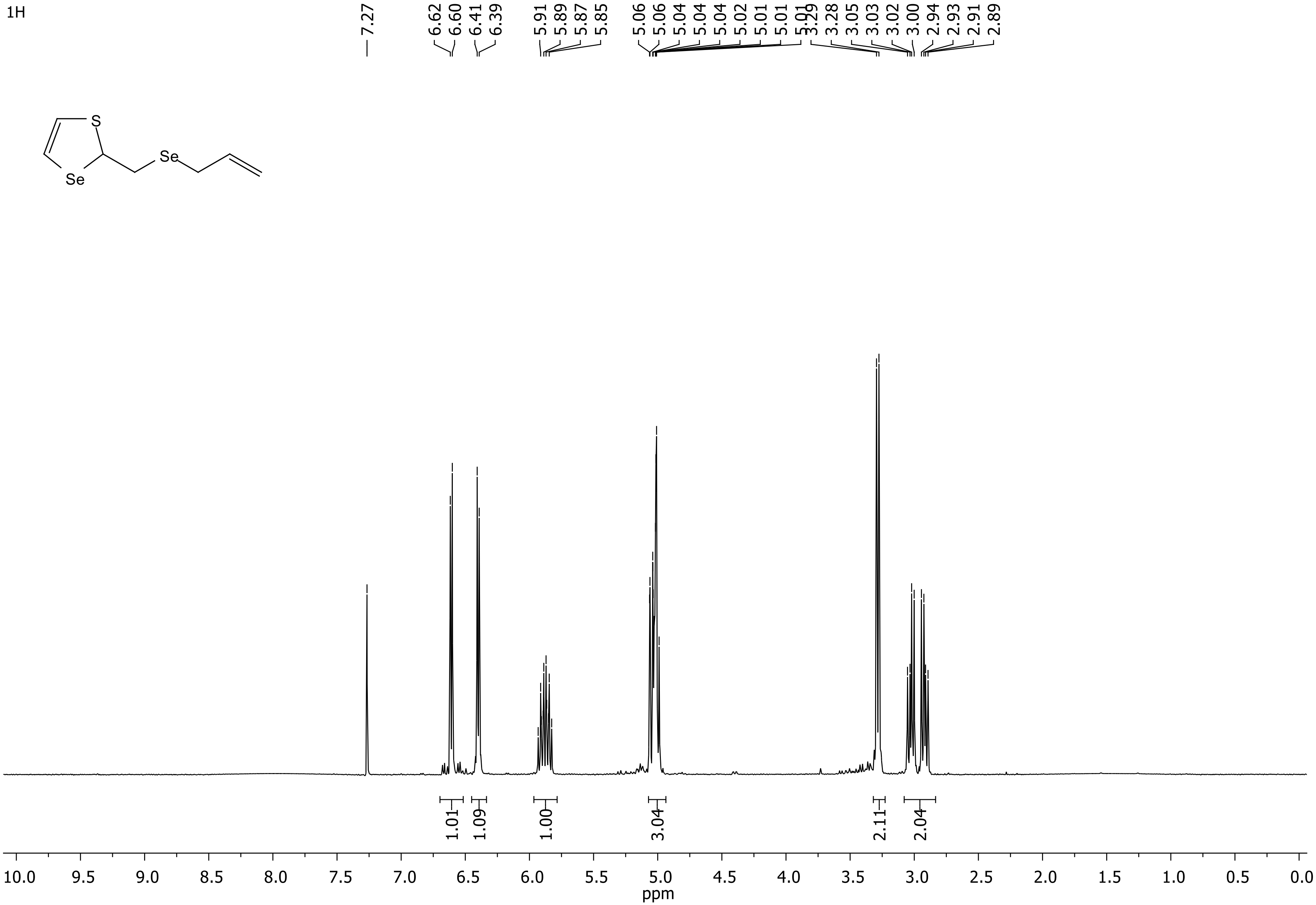

^{1}H NMR spectrum of allyl 1,3-thiaselenol-2-ylmethyl selenide (6i)

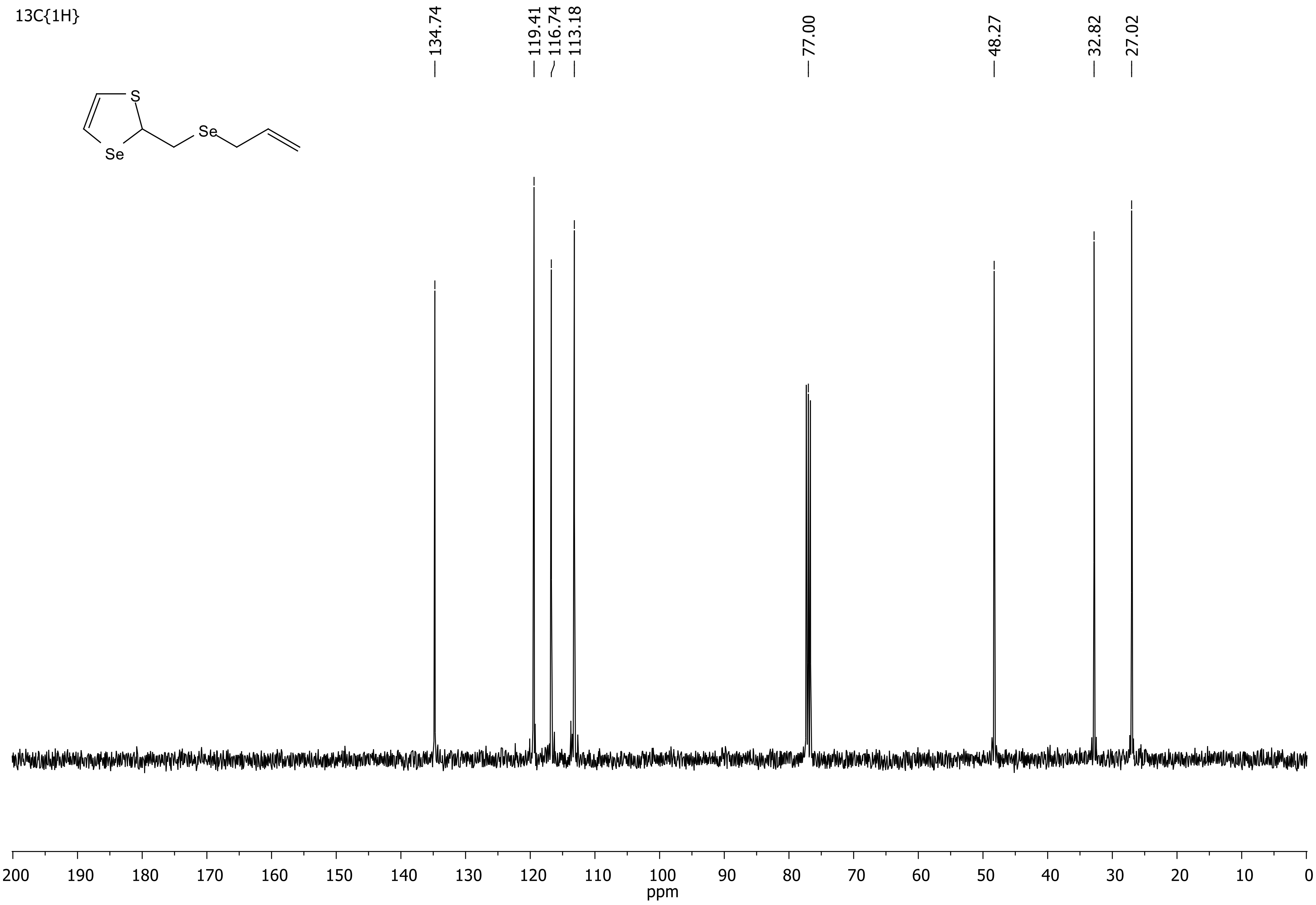

$^{13}C\{^1H\}$ NMR spectrum of allyl 1,3-thiaselenol-2-ylmethyl selenide (6i)

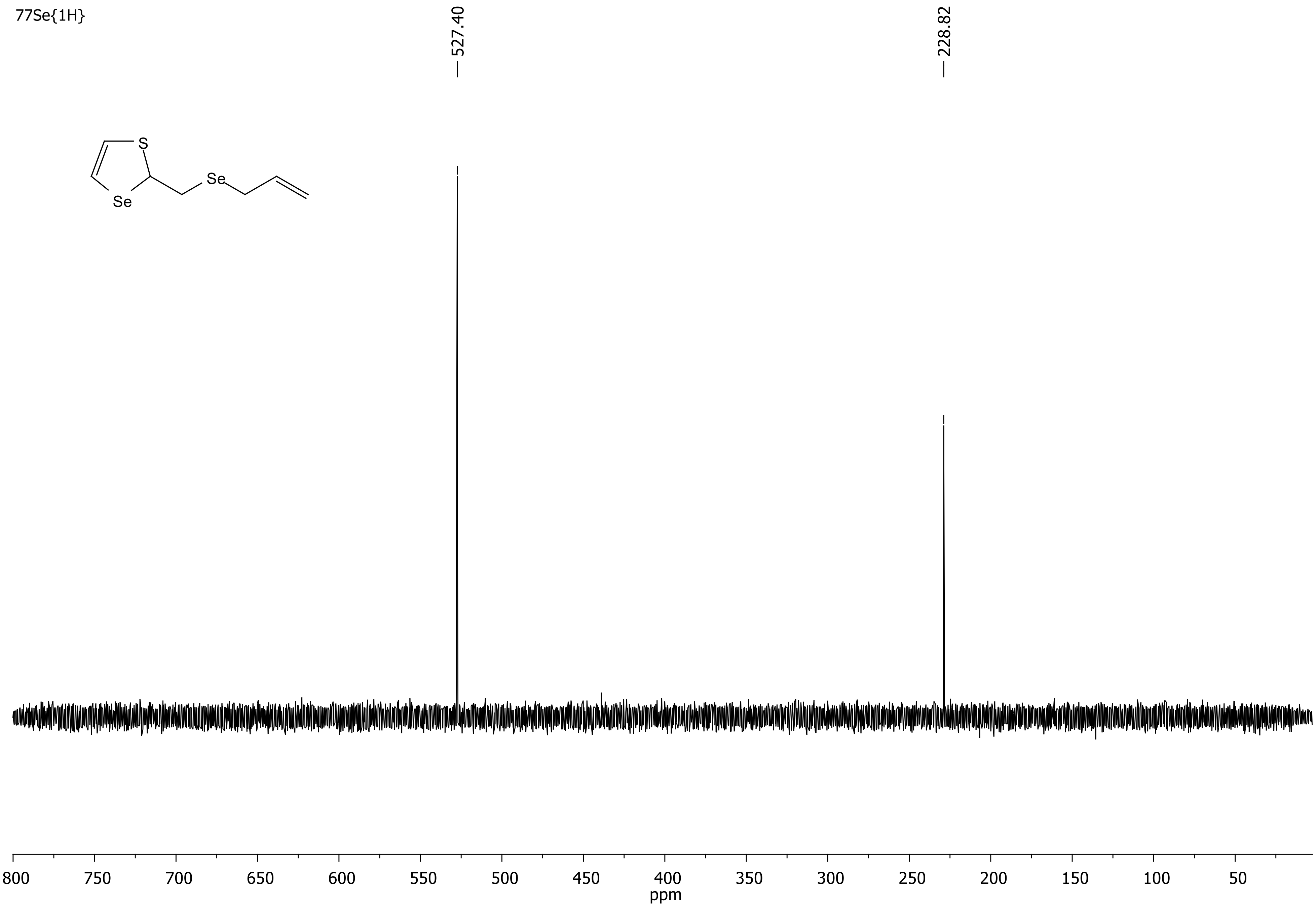

$^{77}Se\{^1H\}$ NMR spectrum of allyl 1,3-thiaselenol-2-ylmethyl selenide (6i)

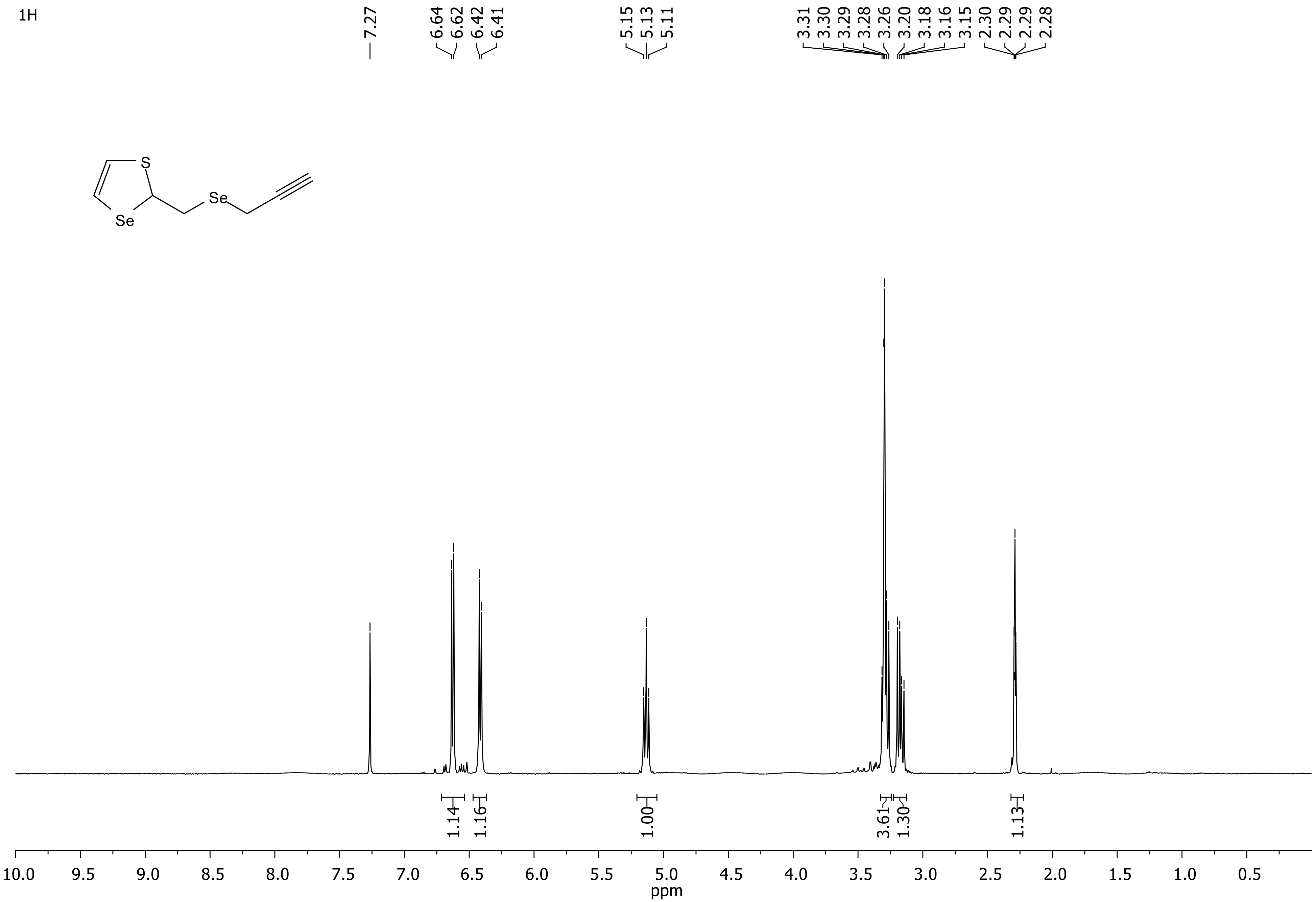

^{1}H NMR spectrum of 2-propynyl 1,3-thiaselenol-2-ylmethyl selenide (6j)

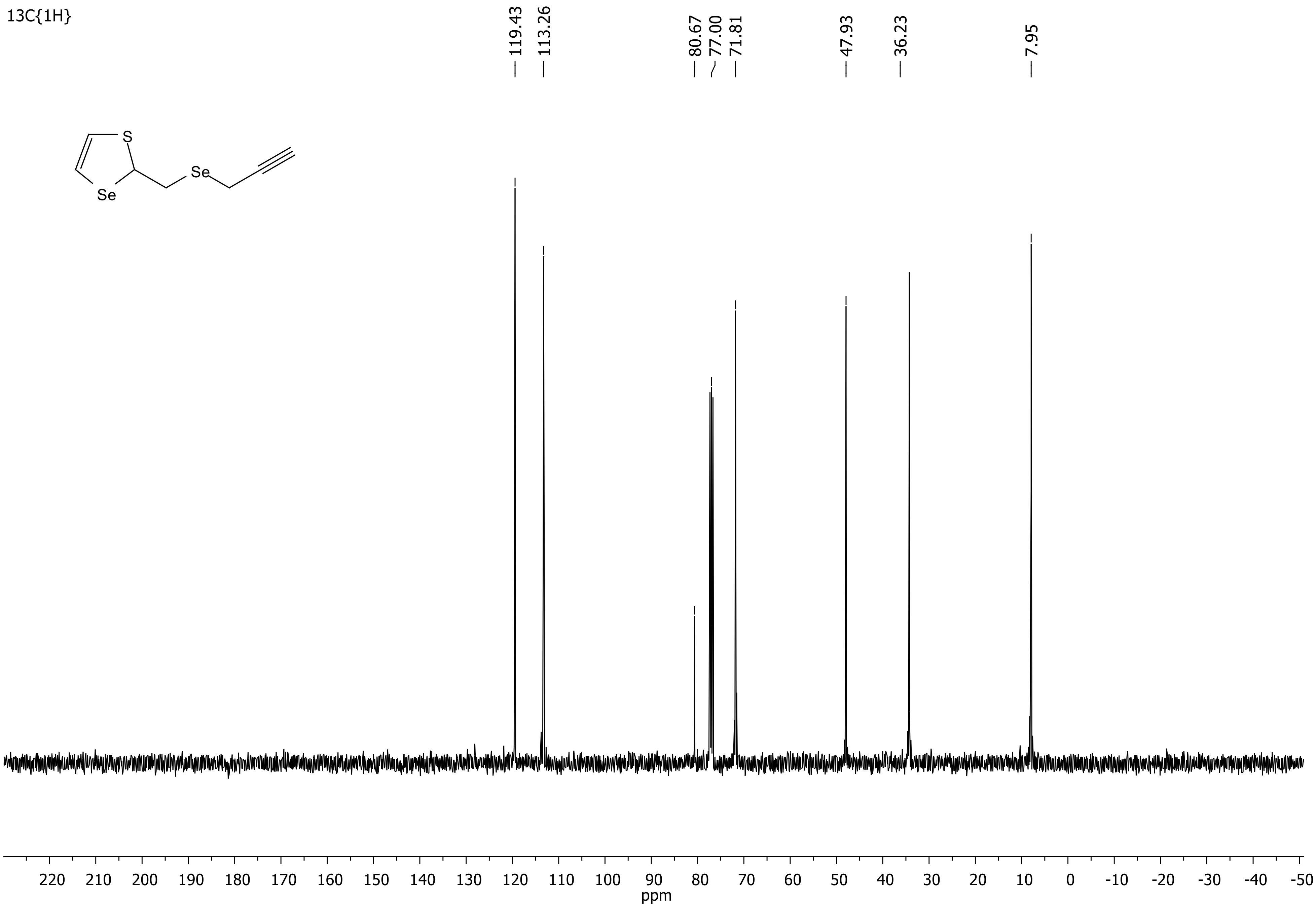

$^{13}C\{^{1}H\}$ NMR spectrum of 2-propynyl 1,3-thiaselenol-2-ylmethyl selenide (6j)

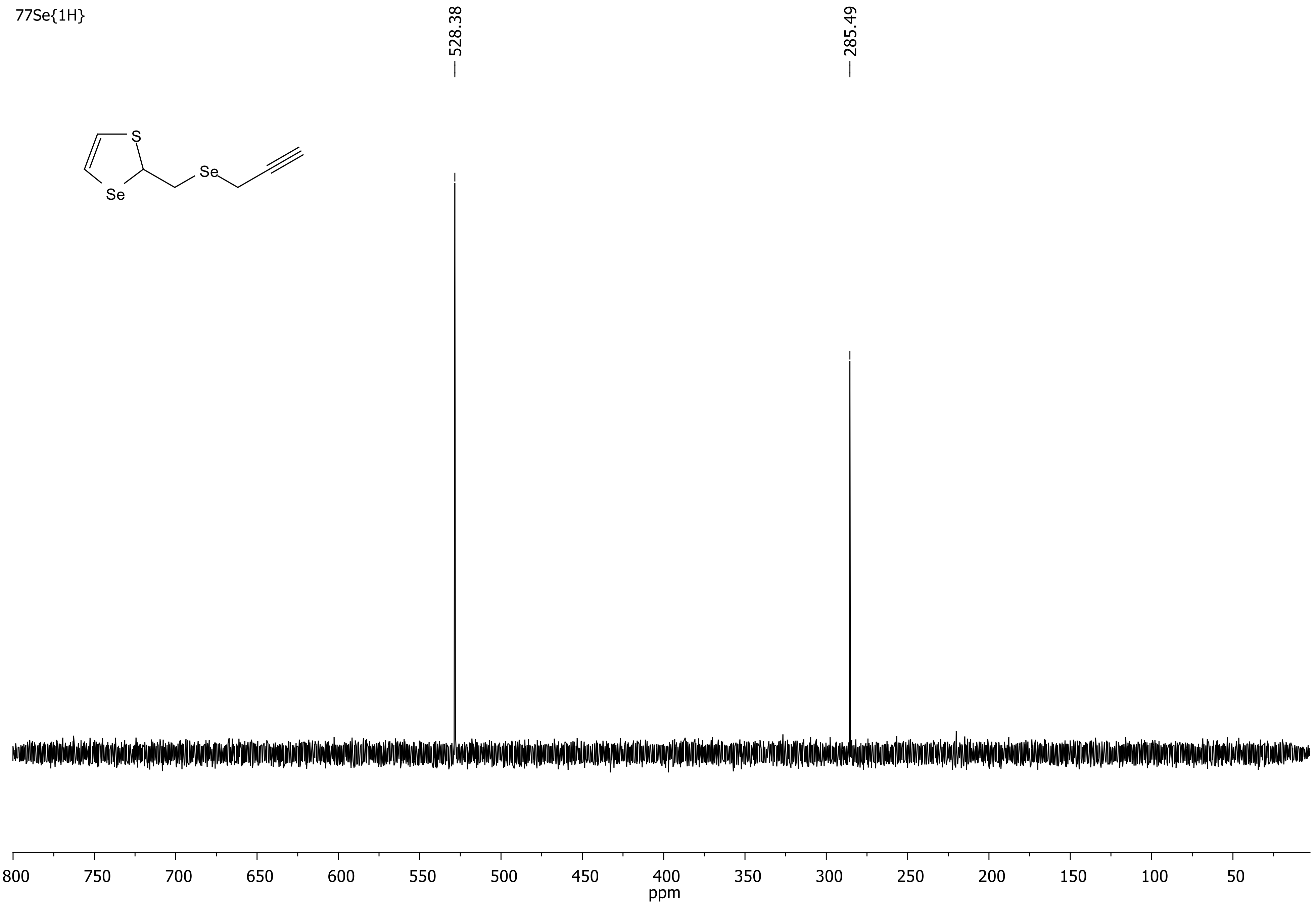

$^{77}Se\{^{1}H\}$ NMR spectrum of 2-propynyl 1,3-thiaselenol-2-ylmethyl selenide (6j)

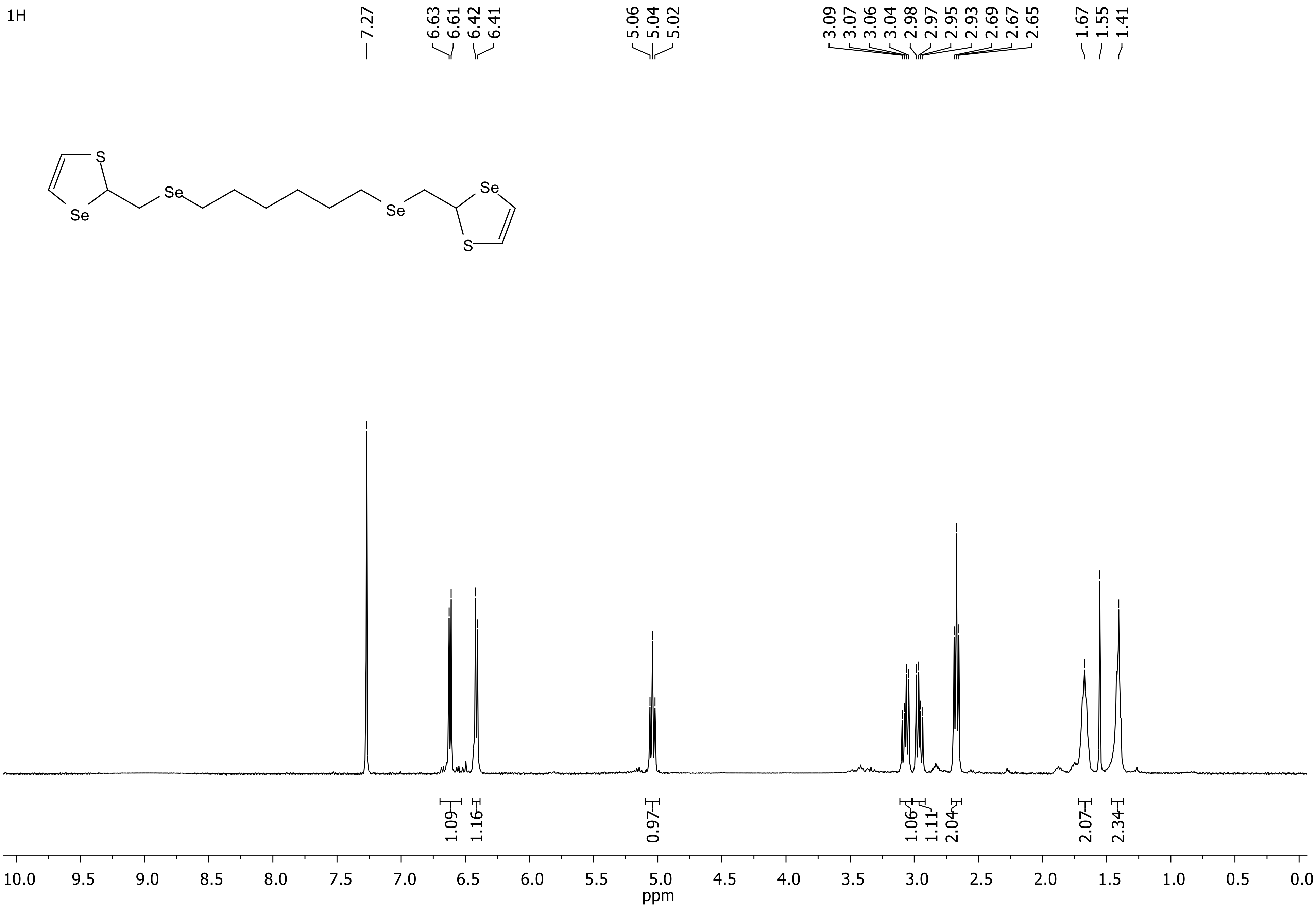

^{1}H NMR spectrum of 1,3-thiaselenol-2-ylmethyl 6-[(1,3-thiaselenol-2-ylmethyl)selanyl]hexyl selenide (6k)

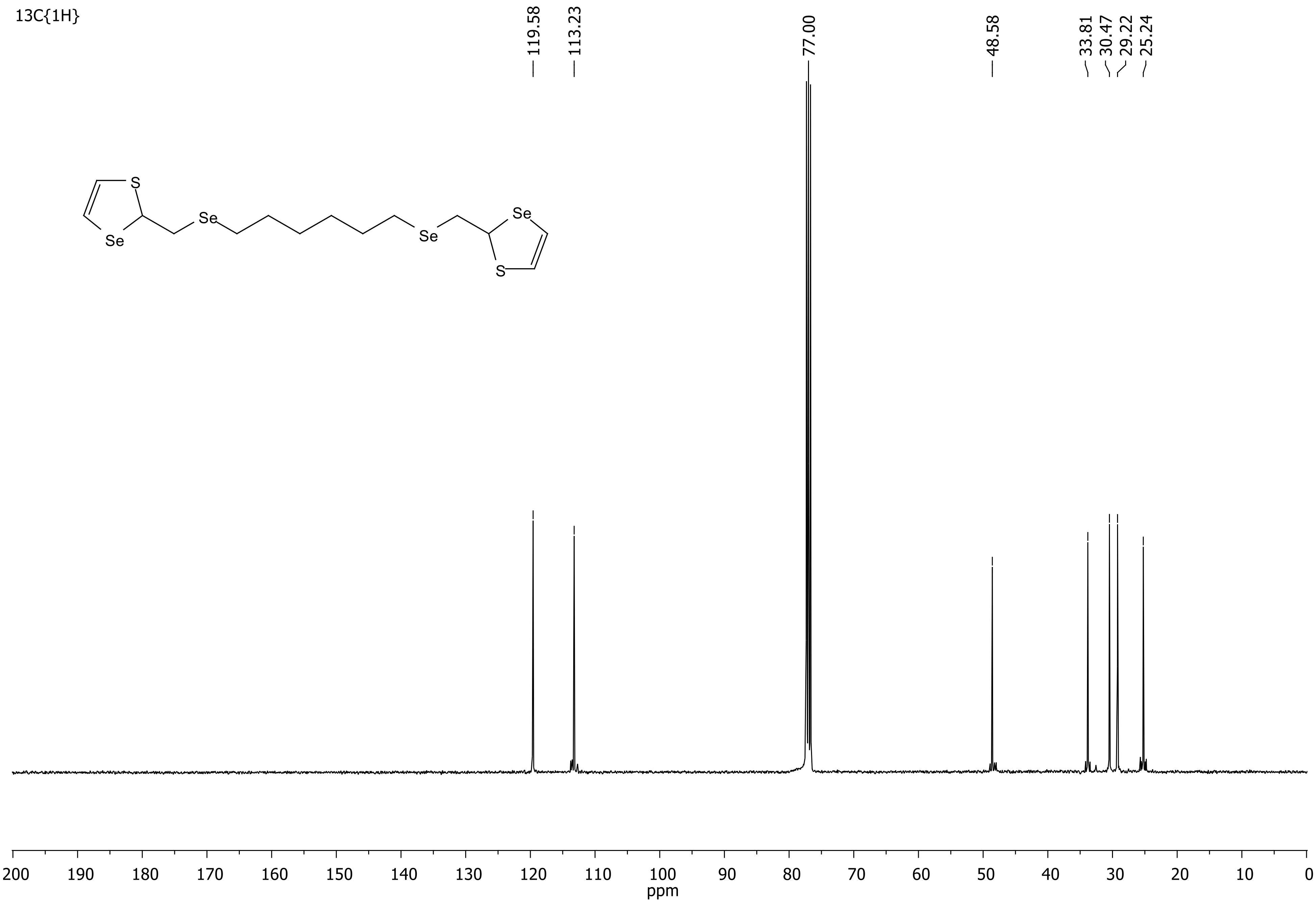

$^{13}C\{^1H\}$ NMR spectrum of 1,3-thiaselenol-2-ylmethyl 6-[(1,3-thiaselenol-2-ylmethyl)selanyl]hexyl selenide (6k)

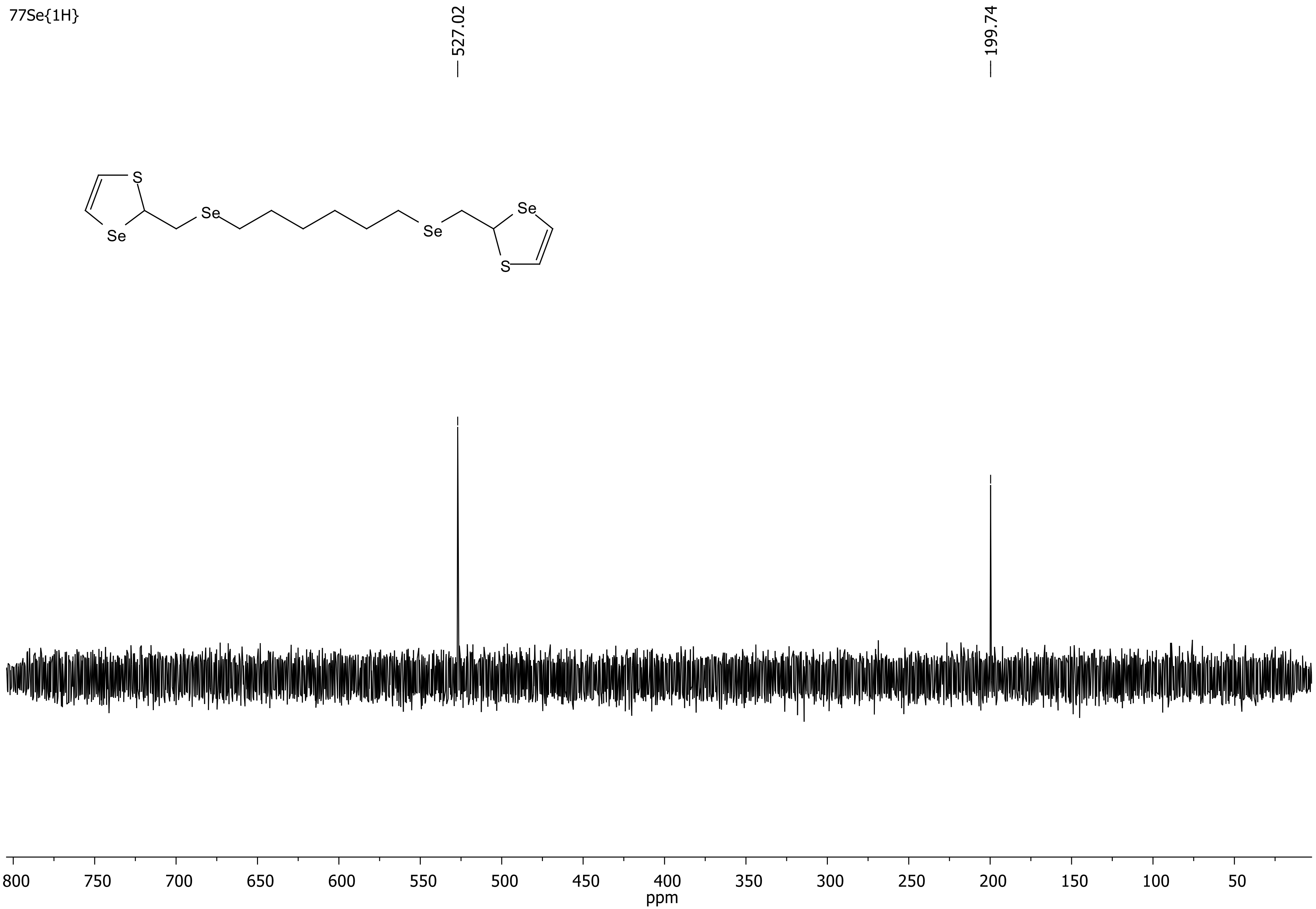

$^{77}Se\{^{1}H\}$ NMR spectrum of 1,3-thiaselenol-2-ylmethyl 6-[(1,3-thiaselenol-2-ylmethyl)selanyl]hexyl selenide (6k)

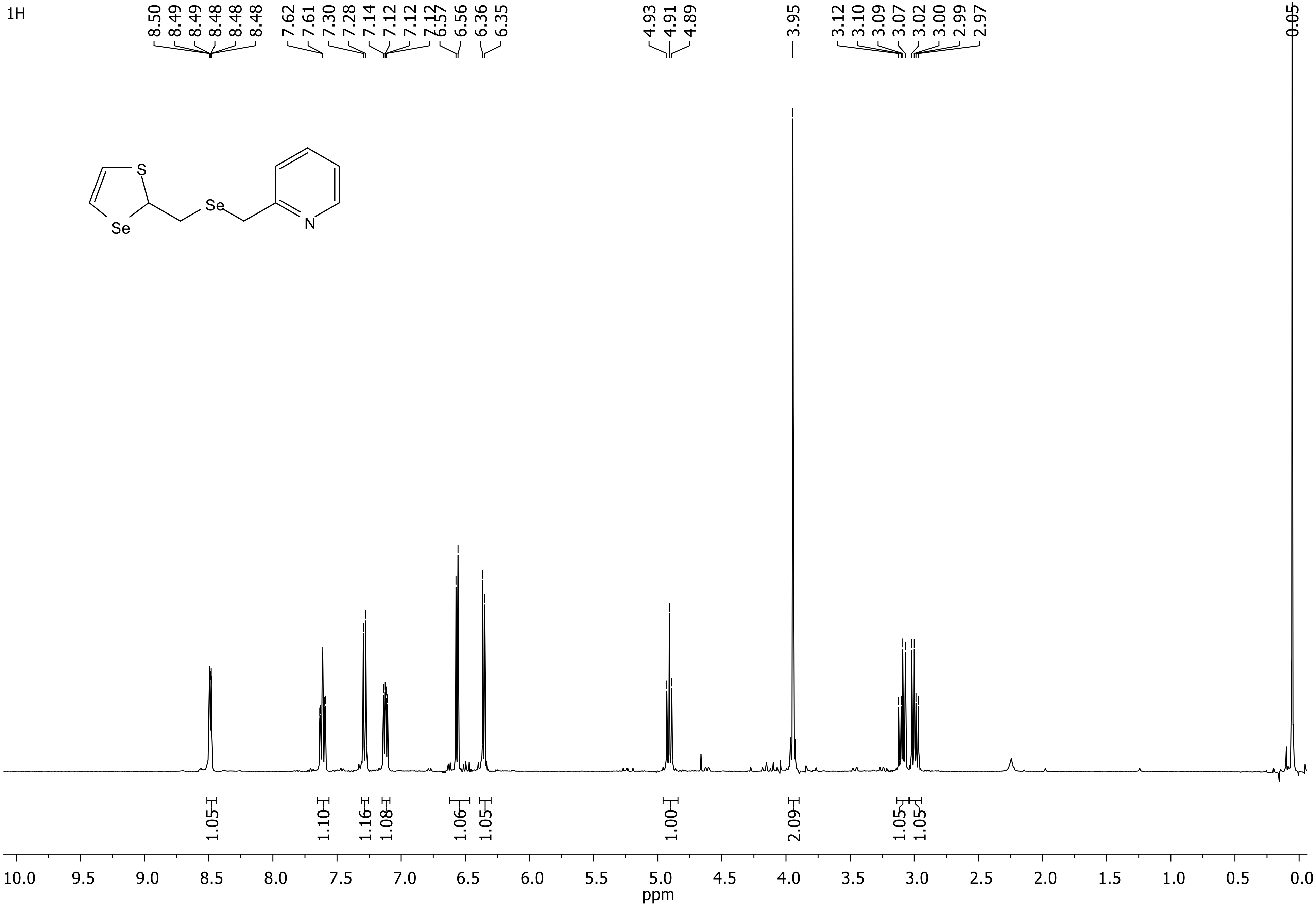

^{1}H NMR spectrum of 2-pyridinylmethyl 1,3-thiaselenol-2-ylmethyl selenide (6l)

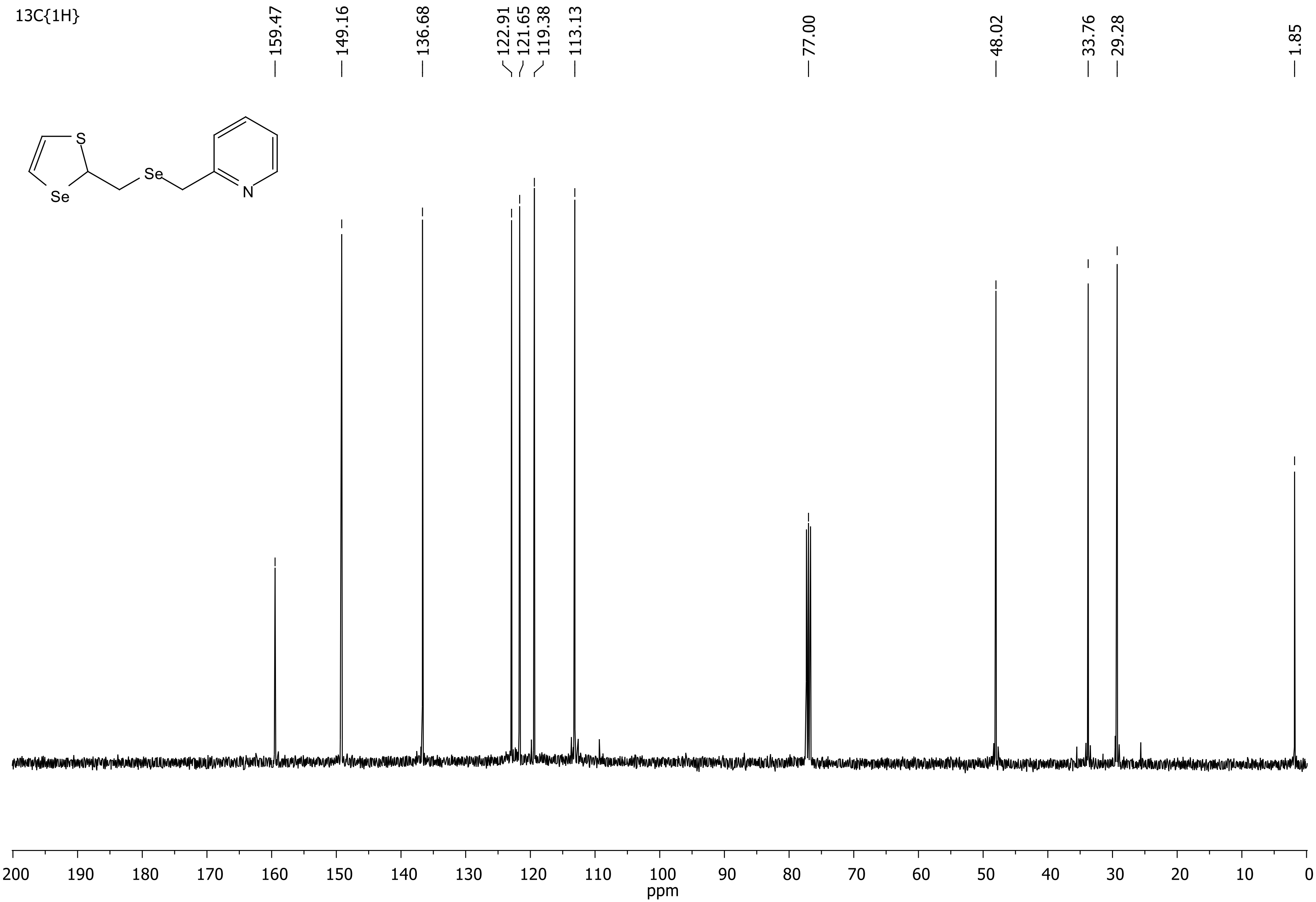

$^{13}C\{^{1}H\}$ NMR spectrum of 2-pyridinylmethyl 1,3-thiaselenol-2-ylmethyl selenide (6l)

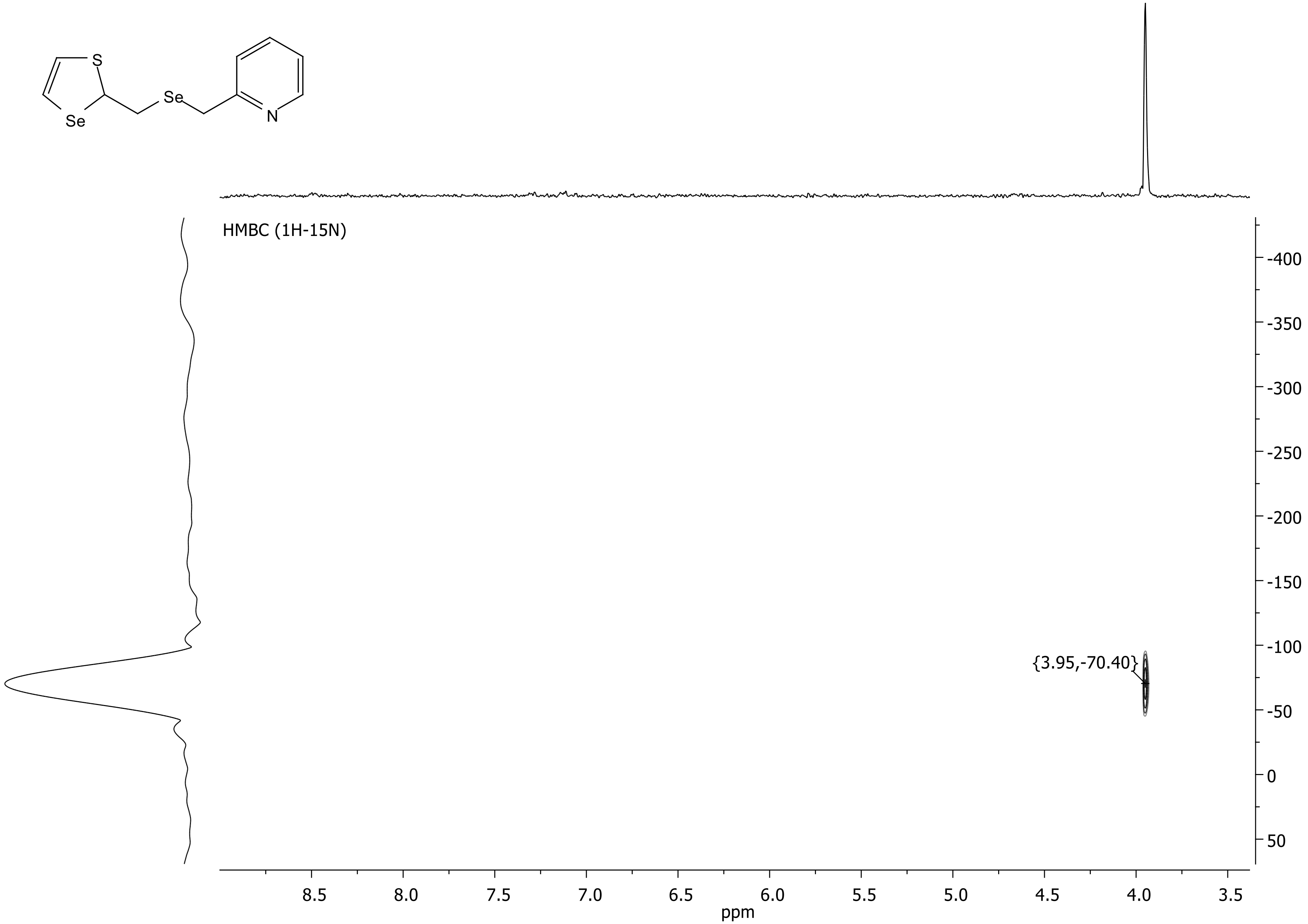

HMBC (^{1}H-^{15}N) NMR spectrum of 2-pyridinylmethyl 1,3-thiaselenol-2-ylmethyl selenide (6l)

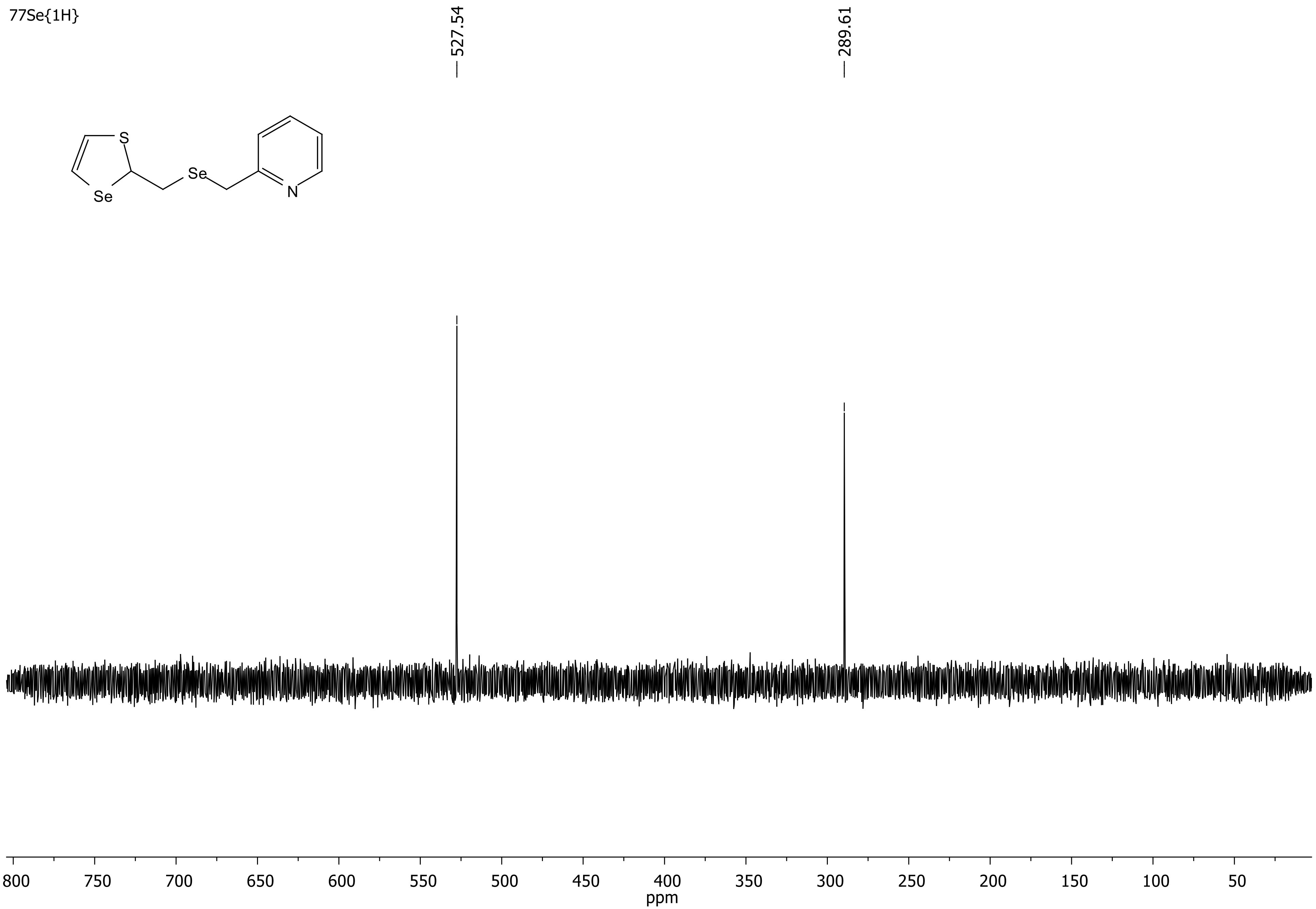

^{77}Se{^{1}H} NMR spectrum of 2-pyridinylmethyl 1,3-thiaselenol-2-ylmethyl selenide (6l)

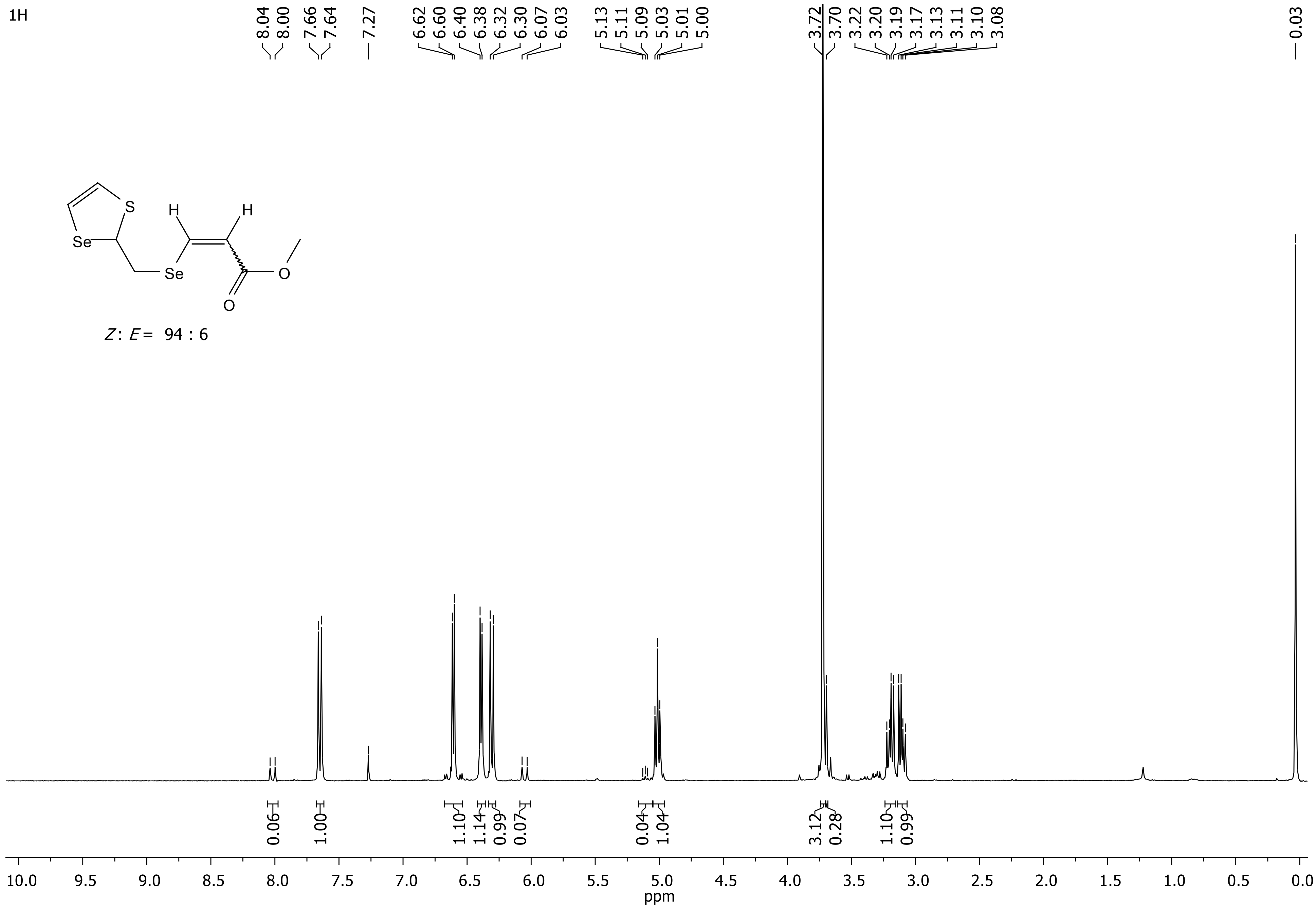

^{1}H NMR spectrum of methyl (*Z*)-, (*E*)-3-[(1,3-thiaselenol-2-ylmethyl)selanyl]-2-propenoate (7a)

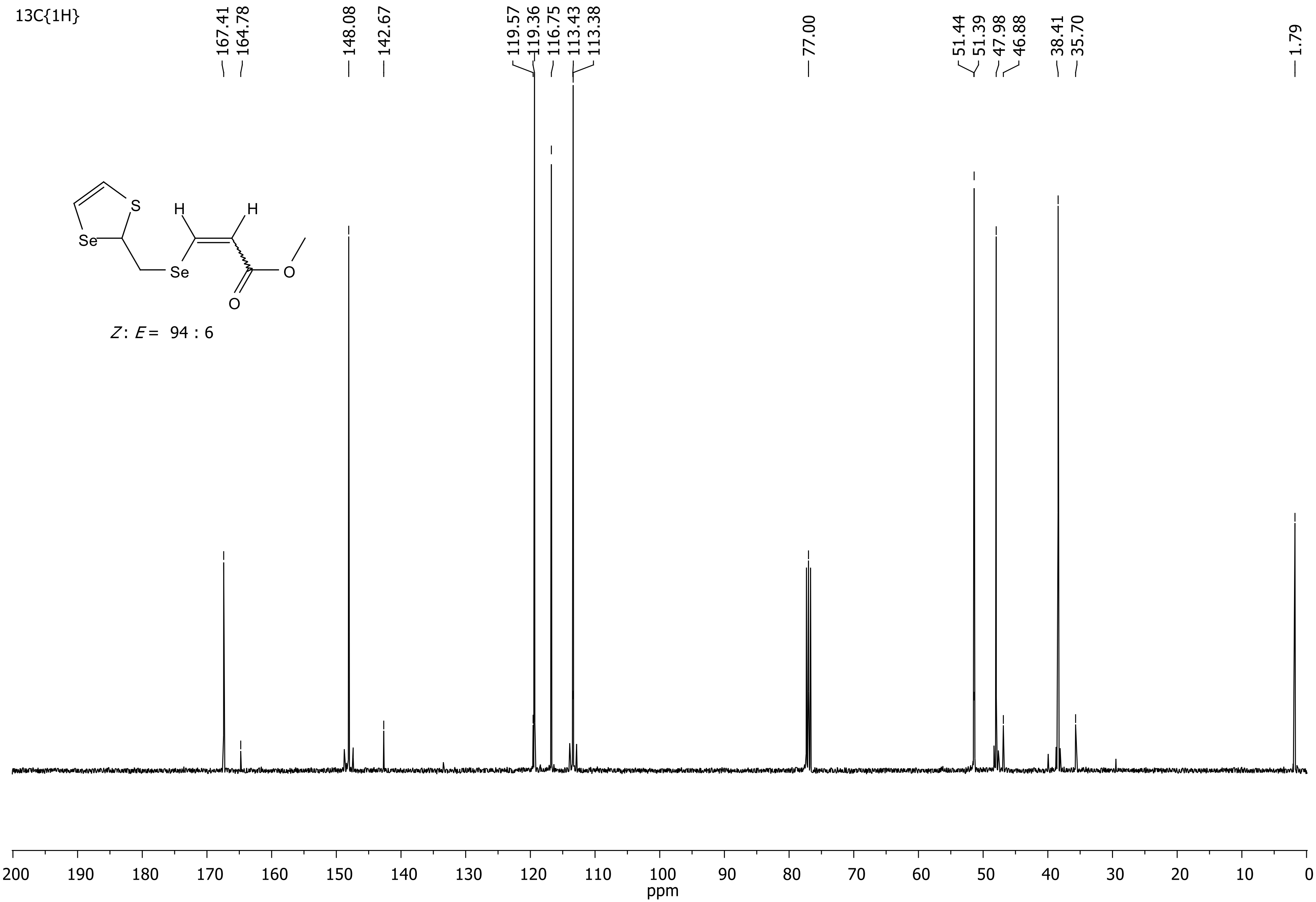

$^{13}C\{^{1}H\}$ NMR spectrum of methyl (*Z*)-, (*E*)-3-[(1,3-thiaselenol-2-ylmethyl)selanyl]-2-propenoate (7a)

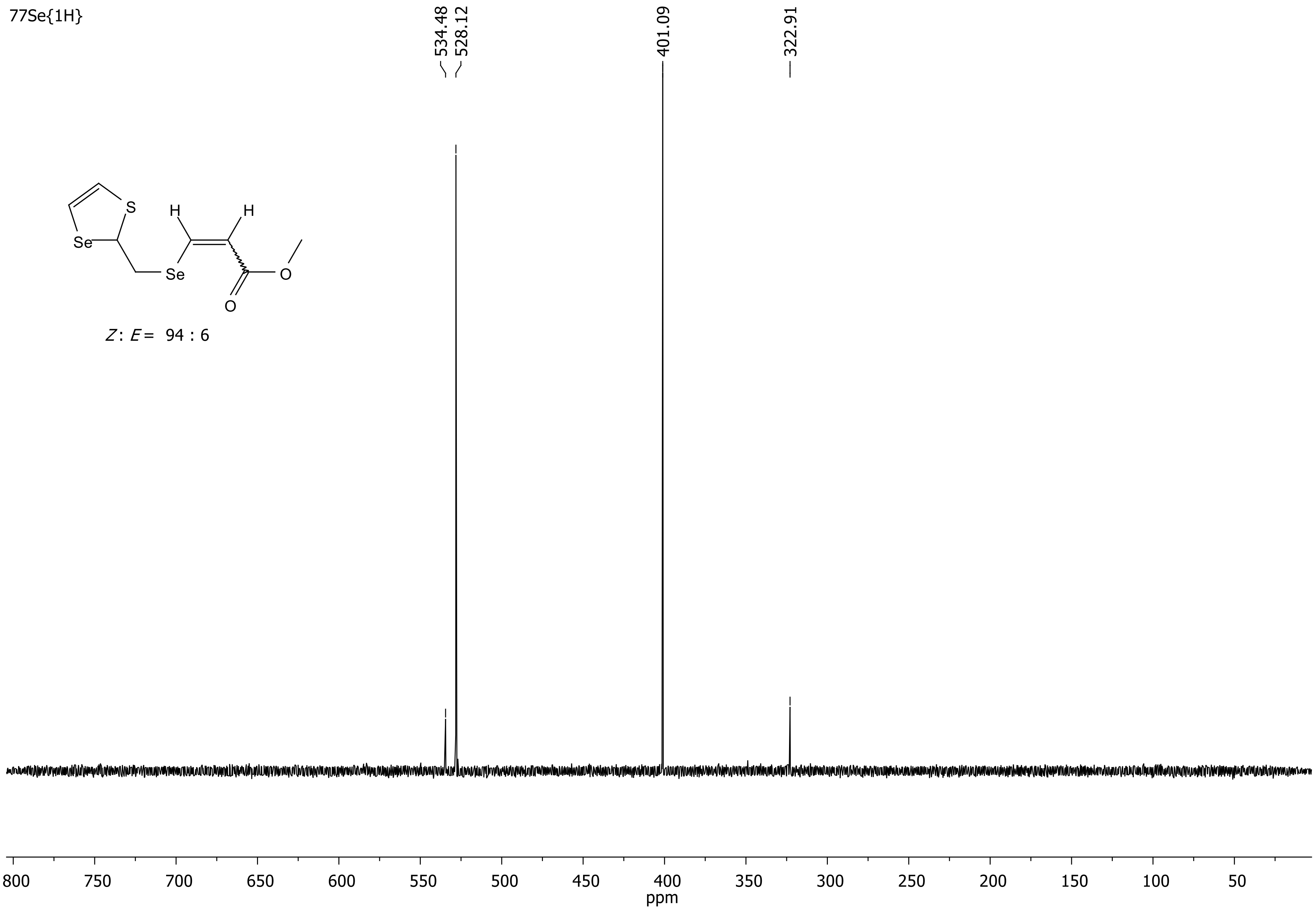

$^{77}Se\{^{1}H\}$ NMR spectrum of methyl (*Z*)-, (*E*)-3-[(1,3-thiaselenol-2-ylmethyl)selanyl]-2-propenoate (7a)

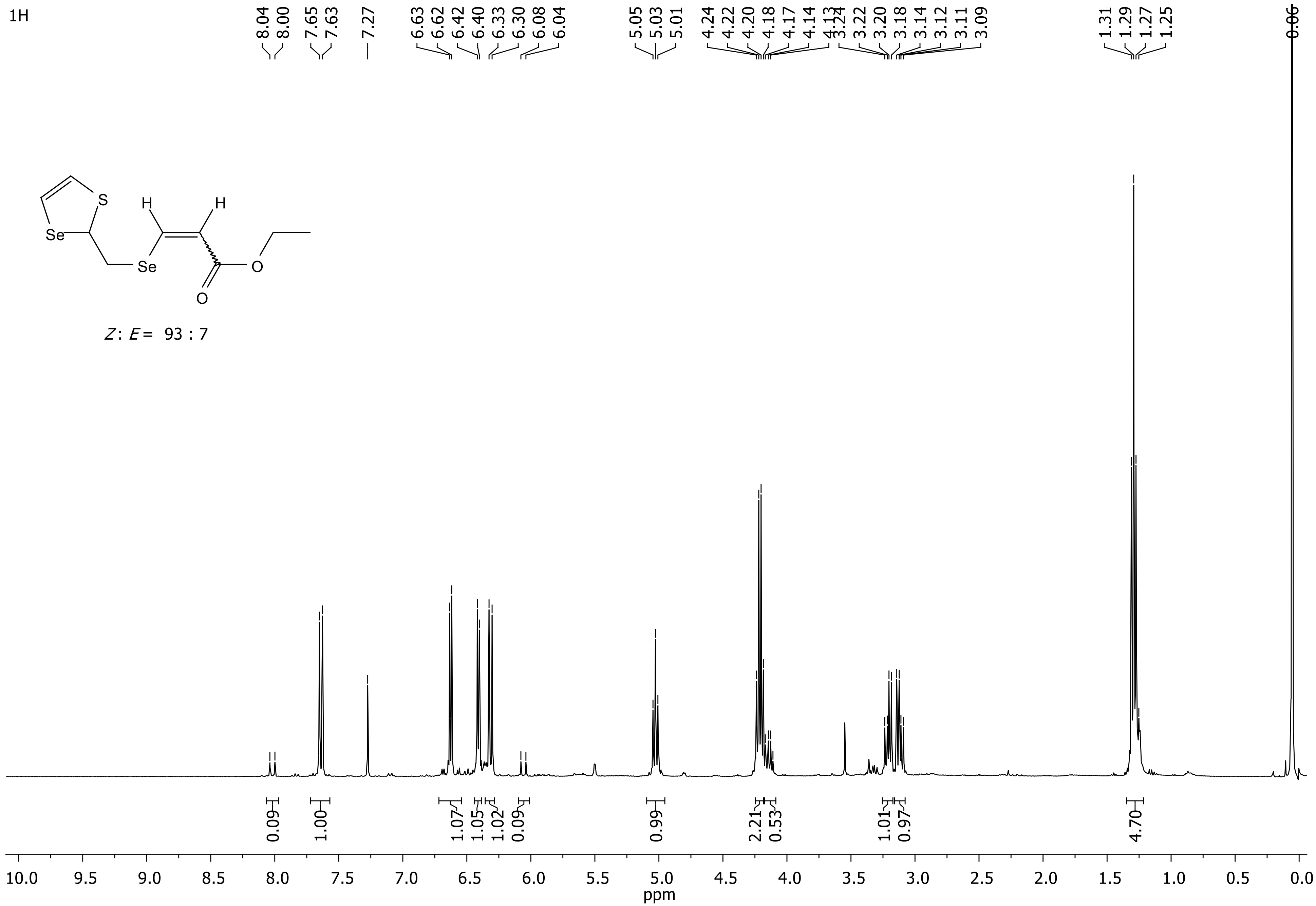

^{1}H NMR spectrum of ethyl (*Z*)-, (*E*)-3-[(1,3-thiaselenol-2-ylmethyl)selanyl]-2-propenoate (7b)

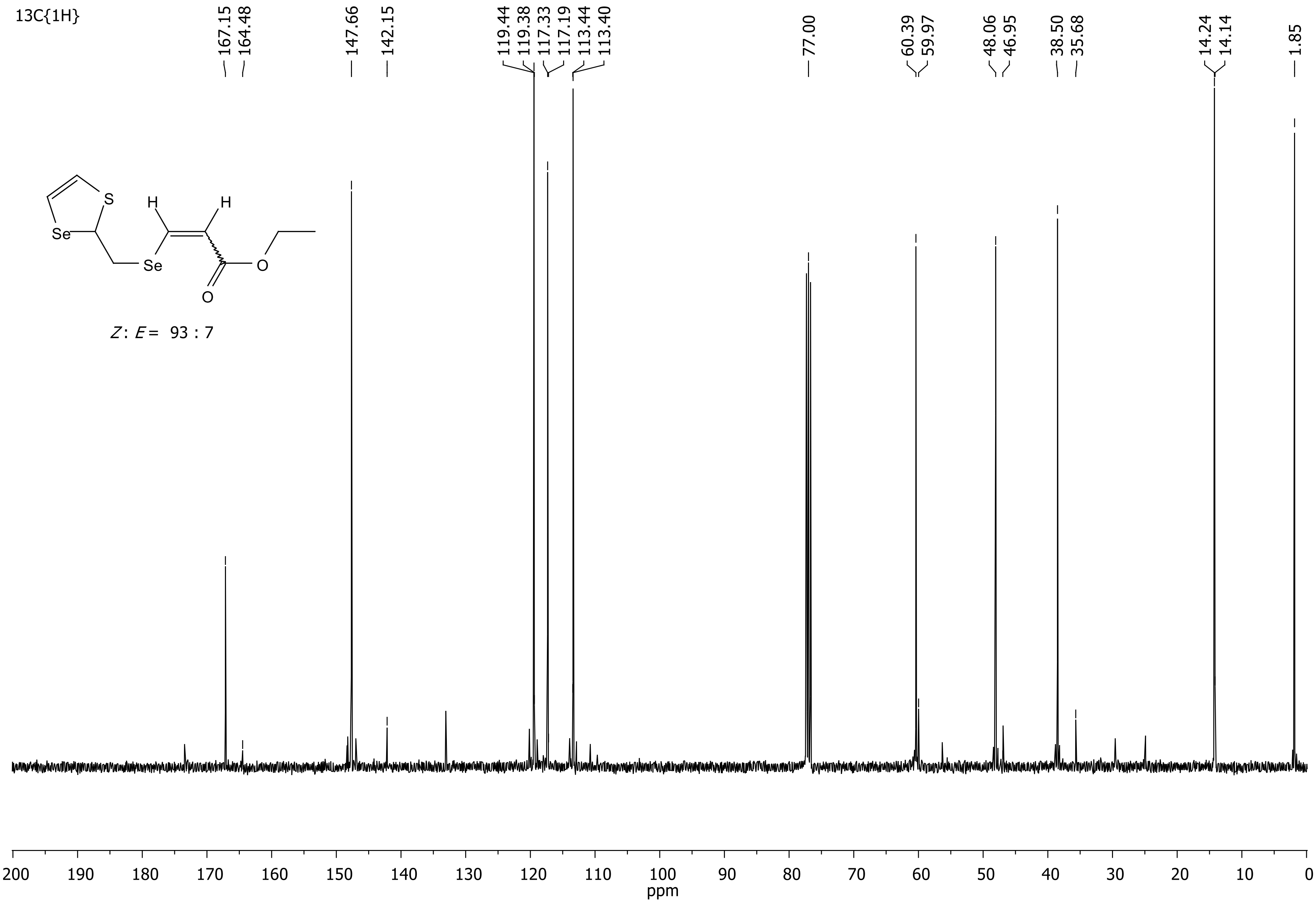

$^{13}C\{^1H\}$ NMR spectrum of ethyl (*Z*)-, (*E*)-3-[(1,3-thiaselenol-2-ylmethyl)selanyl]-2-propenoate (7b)

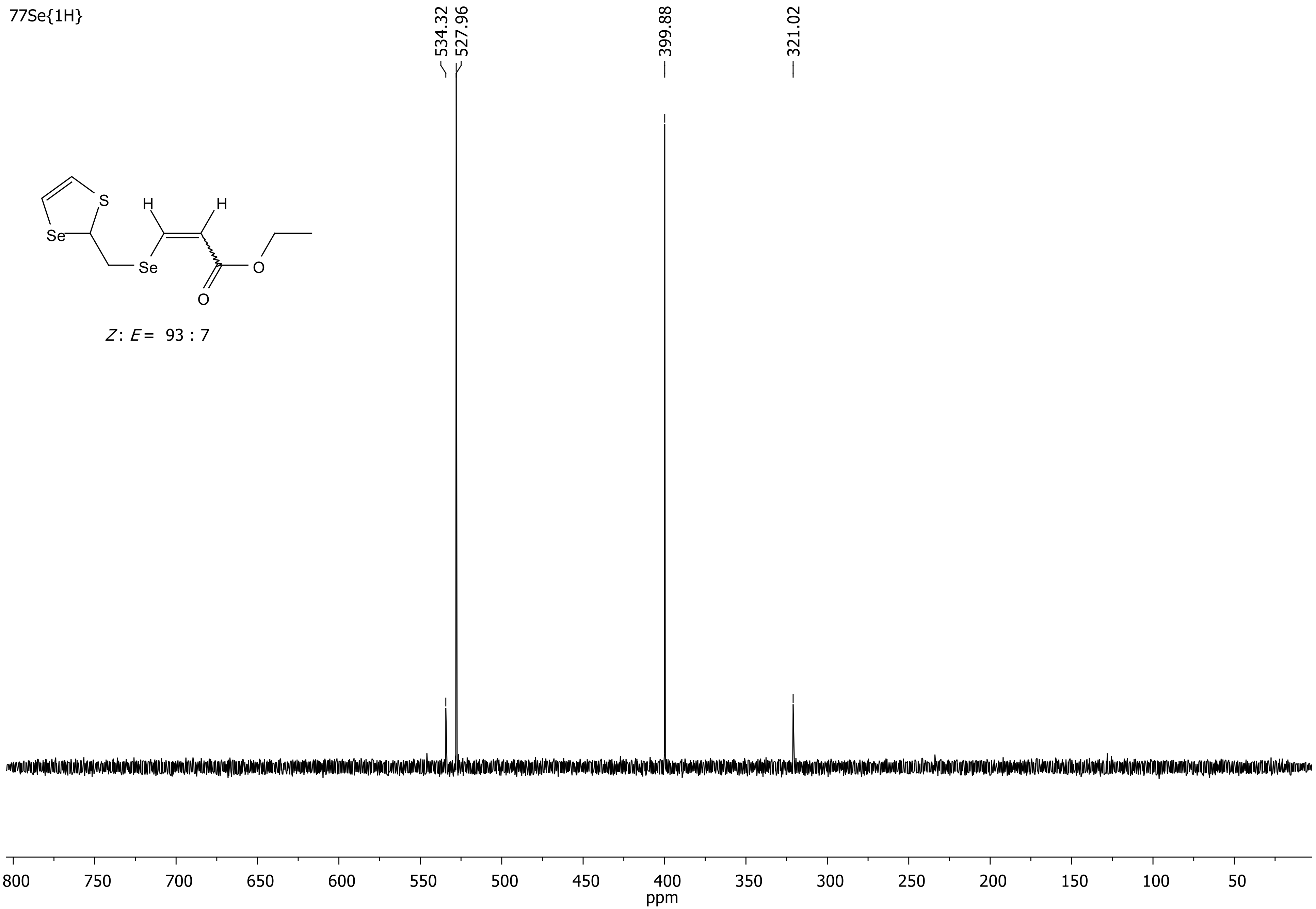

$^{77}Se\{^{1}H\}$ NMR spectrum of ethyl (*Z*)-, (*E*)-3-[(1,3-thiaselenol-2-ylmethyl)selanyl]-2-propenoate (7b)

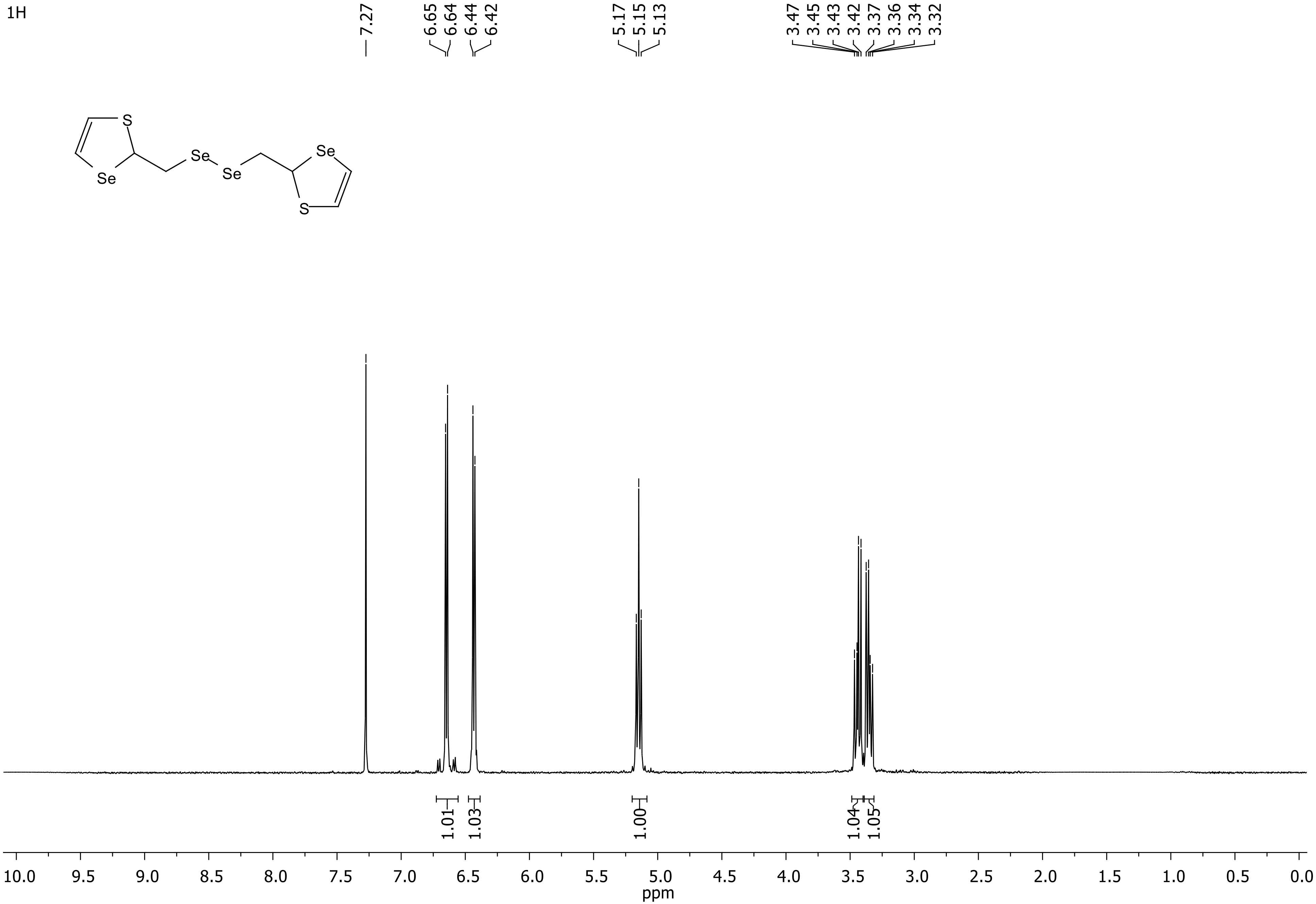

^{1}H NMR spectrum of 2-[2-(1,3-thiaselenol-2-ylmethyl)diselanyl]methyl-1,3-thiaselenole (8)

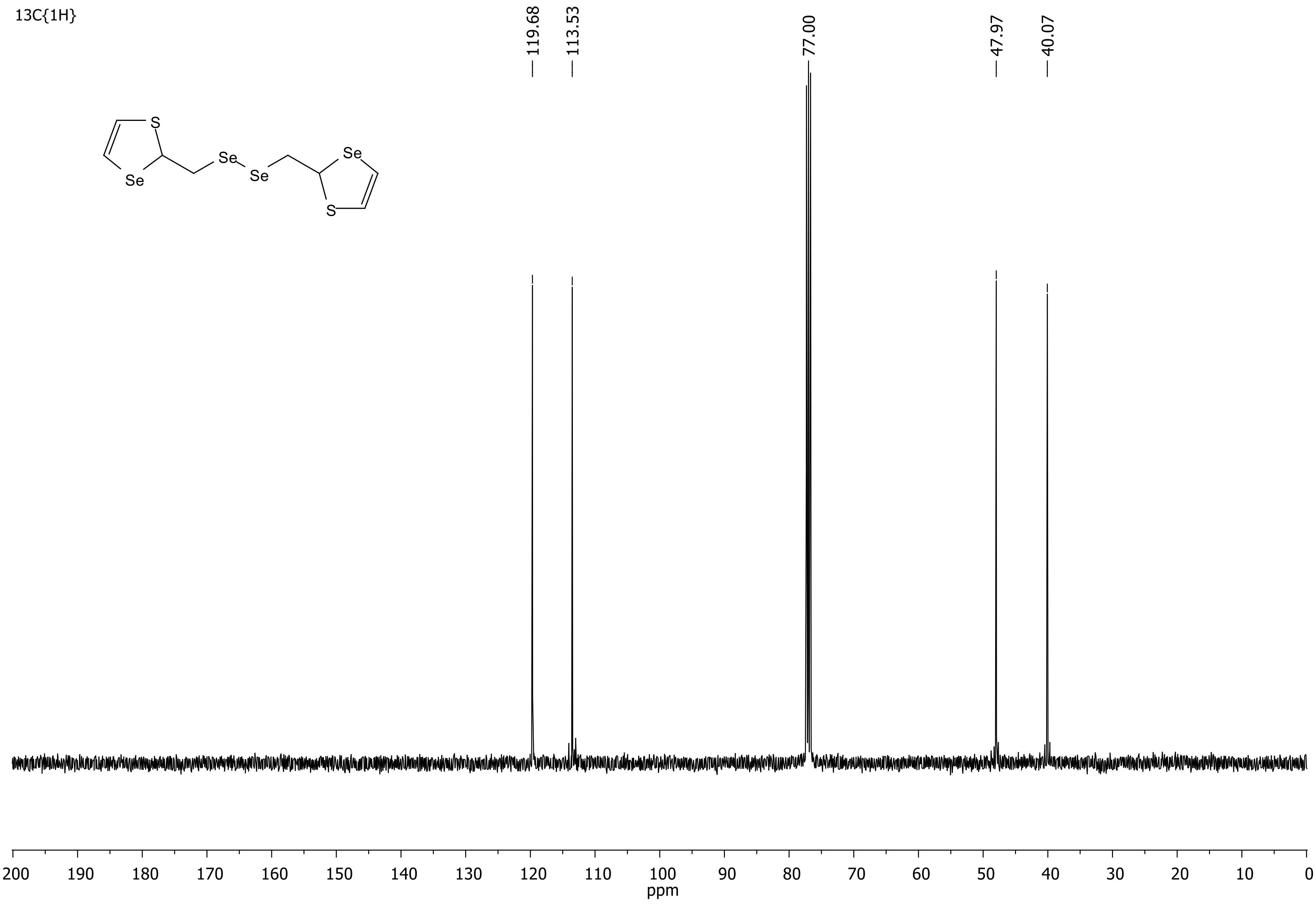

$^{13}C\{^{1}H\}$ NMR spectrum of 2-[2-(1,3-thiaselenol-2-ylmethyl)diselanyl]methyl-1,3-thiaselenole (8)

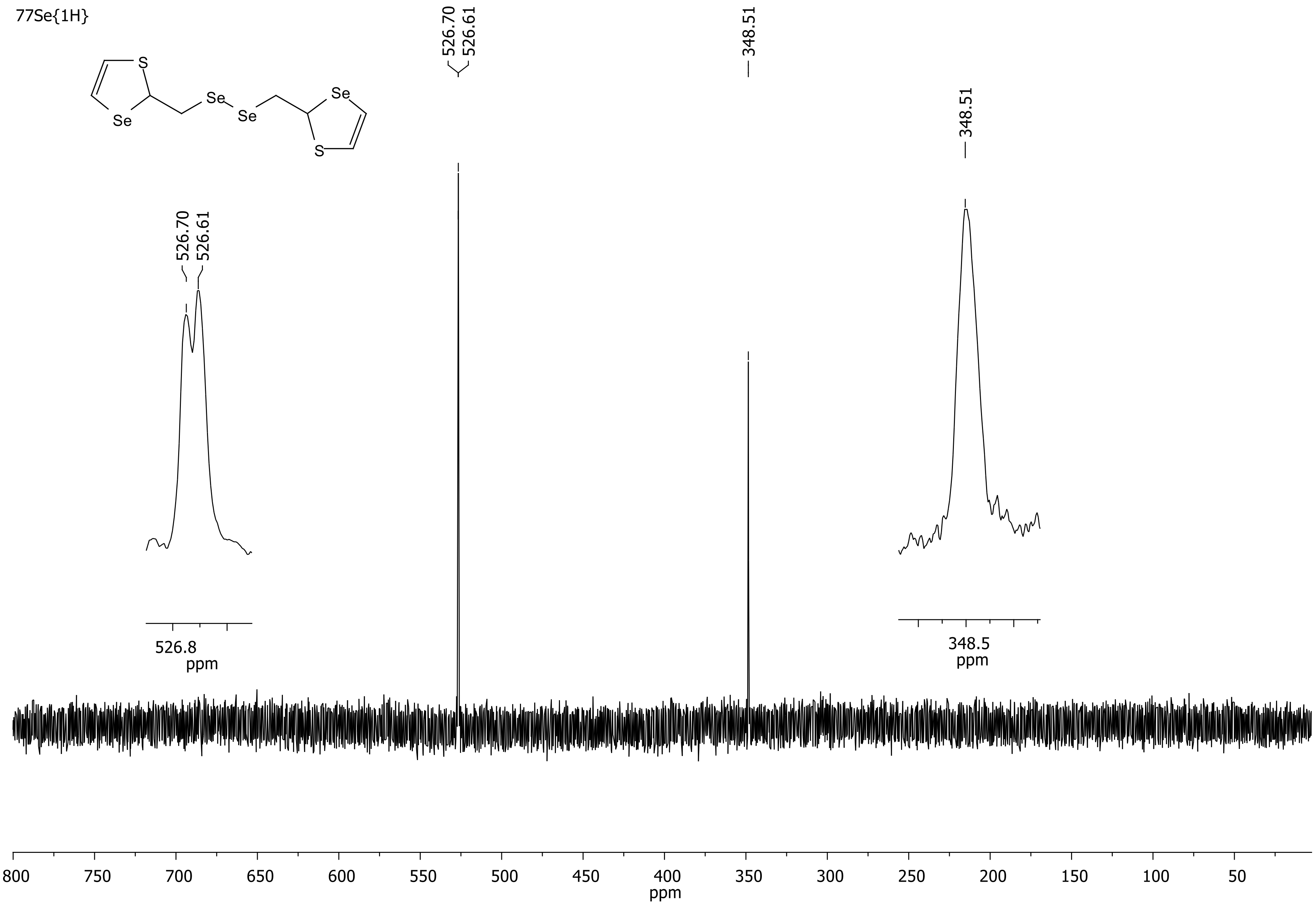

^{77}Se{^{1}H} NMR spectrum of 2-[2-(1,3-thiaselenol-2-ylmethyl)diselanyl]methyl-1,3-thiaselenole (8)
